# Main group catalysis for H_2_ purification based on liquid organic hydrogen carriers

**DOI:** 10.1126/sciadv.ade0189

**Published:** 2022-10-26

**Authors:** Taiki Hashimoto, Takahiro Asada, Sensuke Ogoshi, Yoichi Hoshimoto

**Affiliations:** Department of Applied Chemistry, Faculty of Engineering, Osaka University, Suita, Osaka 565-0871, Japan.

## Abstract

Molecular hydrogen (H_2_) is one of the most important energy carriers. In the midterm future, a huge amount of H_2_ will be produced from a variety of hydrocarbon sources through conversion and removal of contaminants such as CO and CO_2_. However, bypassing these purification processes is desirable, given their energy consumption and environmental impact, which ultimately increases the cost of H_2_. Here, we demonstrate a strategy to separate H_2_ from a gaseous mixture of H_2_/CO/CO_2_/CH_4_ that can include an excess of CO and CO_2_ relative to H_2_ and simultaneously store it in N-heterocyclic compounds that act as liquid organic hydrogen carriers (LOHCs), which can be applied to produce H_2_ by subsequent dehydrogenation. Our results demonstrate that LOHCs can potentially be used for H_2_ purification from CO- and CO_2_-rich crude H_2_ in addition to their well-established use in H_2_ storage.

## Teaser

A H_2_ purification system has been achieved based on LOHCs, which goes beyond the well-established use in H_2_ storage.

## INTRODUCTION

**Fig. 1.**
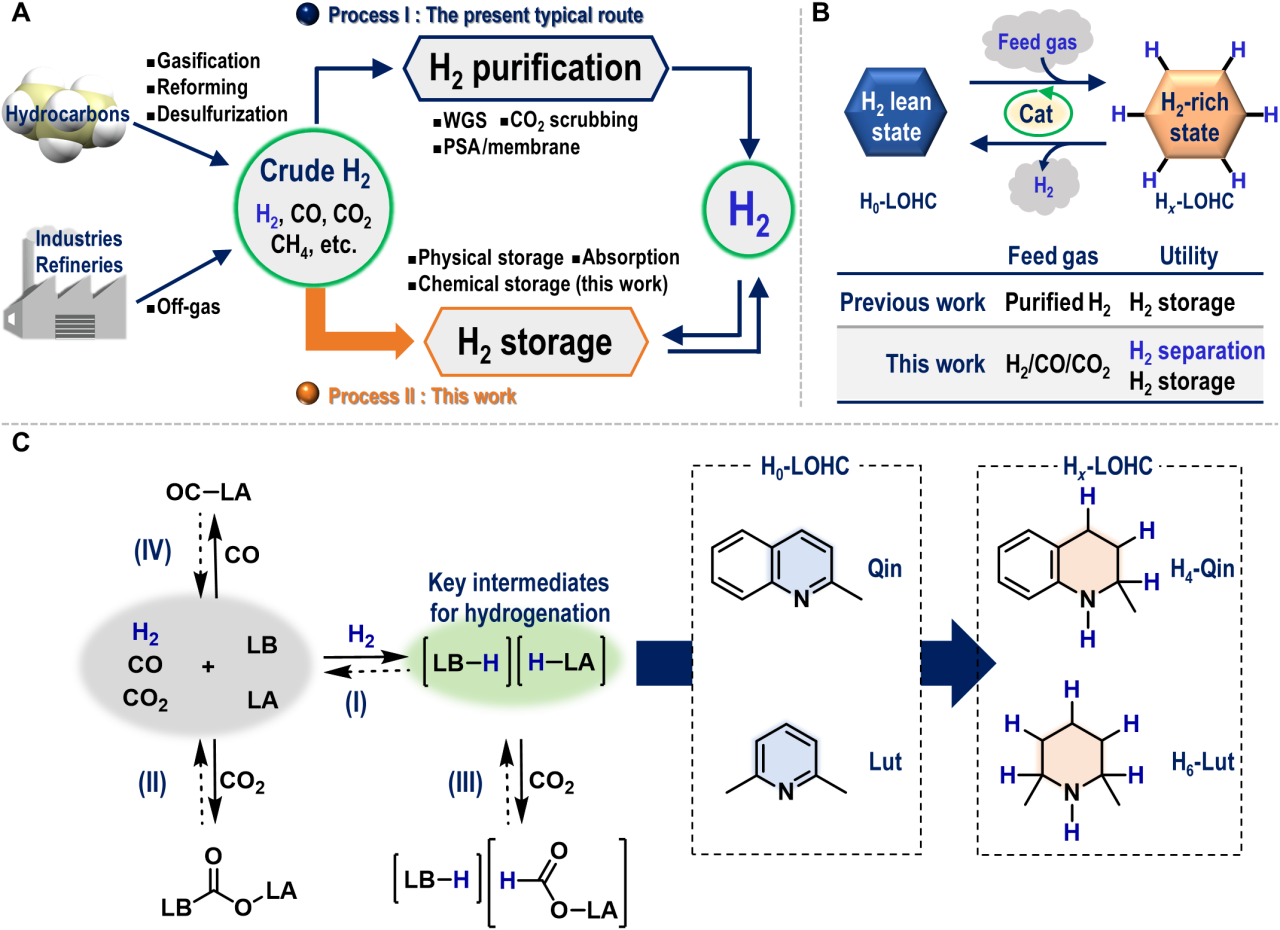
Research background and concept of this study. (**A**) Simplified schemes of representative contemporary routes of H_2_ purification followed by H_2_ storage (process I) and a conceptually novel route involving the simultaneous separation and storage of H_2_ from crude H_2_ (process II); WGS, water-gas shift; PSA, pressure swing adsorption. (**B**) Schematic illustration of the general concept behind LOHCs and the critical differences between well-established methods and this work. (**C**) Potential reactions among H_2_, CO, CO_2_, LA (Lewis acid/acidic part), and/or LB (Lewis base/basic part). Dashed arrows represent backward reactions that do not always occur under the same conditions as the corresponding forward reaction.

Molecular hydrogen (H_2_) is an essential reductant that has been widely used in, e.g., petroleum refineries, the industrial production of ammonia and methanol, and the chemical industry. Moreover, H_2_ is one of the most promising energy carriers of the future, given its high stability and thus transportability, its high gravimetric energy density, and the low environmental impact of its combustion product compared to those of hydrocarbon-based energy sources (*1*–*4*). These features make H_2_ an attractive candidate for the construction of a greener and sustainable economy, which is commonly referred to as the “hydrogen economy” (*3*). Thus, it can be expected that a huge amount of H_2_, on a magnitude of more than 10^12^ standard cubic feet per year, will be produced from a wide range of hydrocarbon and renewable resources (*1*, *2*). In this context, H_2_ production combined with CO_2_ capture and storage from hydrocarbon resources such as petroleum, coal, natural gas, and biomass represents a pragmatic choice for the midterm future due to the limited supply of renewable energy (*2*), while the electrolysis of water using electricity obtained from renewable resources seems to be an alternative option in the long-term future (*5*). The predominant contemporary route to H_2_ production includes the intensive purification of crude H_2_, which is a gaseous mixture of H_2_, CO, CO_2_, and other components that is produced by gasification, reforming, and/or water-gas shift (WGS) (process I in Fig. 1A). Purification processes such as pressure swing adsorption (PSA), membrane separation, and cryogenic separation critically determine the purity of the H_2_, which is sometimes required to exceed 99.99% for fuel cells, and influence the total energy consumption of the H_2_ production process, making it cost inefficient. Notable advances have been made to improve the efficiency, H_2_ recovery rate, and reproducibility of H_2_ purification processes (*4*). Nevertheless, an approach that could fundamentally solve all these challenging issues remains to be found (*1*). Thus, although H_2_ can currently be stored after or during the process I in Fig. 1A (*6*), we envisaged a solution where H_2_ could be stored in its carrier directly from crude H_2_, which often includes more CO than H_2_, without the requirement for any of the aforementioned shift and purification processes (process II in Fig. 1A) (*7*). Moreover, the recovery of H_2_ after our proposed path ultimately leads to the production of highly pure H_2_.

To this end, we focused on the use of liquid organic hydrogen carriers (LOHCs), which have been widely used for H_2_ storage and transportation (*8*–*12*). H_2_ storage systems with LOHCs are based on a reaction sequence in which a H_2_ lean state (H_0_-LOHC) is hydrogenated to produce a H_2_-rich state (H*_x_*-LOHC), followed by a subsequent dehydrogenation of H*_x_*-LOHC after storage/transport that regenerates H_2_ and H_0_-LOHC (Fig. 1B). The use of LOHCs has been extensively researched, as the technical, environmental, and economic advantages of H_2_ storage using LOHCs are widely accepted (*8*). Furthermore, the pool of potential candidates for H_0_-LOHCs has recently been expanded from the well-studied aromatic hydrocarbons to include heteroaromatics (*11*–*13*), cyclic dipeptides (*14*), amides (*15*, *16*), cyclic ureas (*17*), and oligoesters (*10*), some of which provide hydrogenated products (H*_x_*-LOHC) with H_2_ storage capacity [H_2_ weight % (wt %)] values that exceed the practical guidelines proposed by the European Union (5.0 wt %) and the U.S. government (5.5 wt %) (*8*). However, hitherto reported H_2_ storage systems using LOHCs have predominantly been based on the use of transition metal catalysts. This presents a critical issue for H_2_ separation in the presence of CO and CO_2_, both of which can severely inhibit transition metal–catalyzed hydrogenation reactions (*6*, *9*). We have successfully showed a strategy for H_2_ separation from multicomponent gas mixtures such as H_2_/CO/CO_2_ via the main group–catalyzed hydrogenation of organic molecules in 2017 (*7*). Note here that Breakman-Danheux *et al*. (*18*) in 1996 and Jorschick *et al*. (*19*) in 2019 have independently reported transition metal–based heterogeneous compounds that have been applied to the hydrogenation of hydrocarbon-based LOHCs using contaminated H_2_ including CO, CO_2_, and/or gaseous hydrocarbons, which led to a marked suppression of the catalytic activity by CO despite the great excess of H_2_ present.

Against this background, we have focused on main group catalysis (*20*, *21*) including the use of frustrated Lewis pairs (FLPs) that are composed of Lewis bases (LBs) and triaryl boranes as the Lewis acids (LAs) (*22*–*24*). Triaryl boranes of the type **B*^n^*** such as B(C_6_F_5_)_3_ (**B**^**1**^) have been reported to catalyze the hydrogenation of N-heteroaromatic compounds such as 2-methylquinoline (**Qin**) under diluted conditions (*25*–*27*). FLPs are well known to mediate the heterolytic cleavage of the H─H bond to generate [LB─H][H─LA] species (Fig. 1C, I) (*23*). The subsequent proton/hydride transfer from [LB─H][H─LA] to N-heteroaromatic compounds facilitates the storage of H_2_ (*26*). CO_2_ fixation by FLPs has also been widely studied and found to proceed in either a reversible or irreversible manner (e.g., Fig. 1C, II) (*28*, *29*). The hydrogenation of CO_2_ has been reported in the presence of FLPs that are composed of **B**^**1**^ and nitrogen-based LBs (Fig. 1C, III) (*29*–*32*). CO can reversibly bind to the boron center (Fig. 1C, IV), which would kinetically affect the H_2_ cleavage step (*29*, *33*). Moreover, these gases contain a certain amount of H_2_O, which often triggers the decomposition of triaryl boranes to yield, e.g., [LB─H][HO─LA], although sophisticated strategies to minimize the influence of H_2_O have been reported (*34*, *35*). The reactions shown in Fig. 1C (II to VI) can seriously affect the progress of the targeted catalytic hydrogenation in the presence of CO, CO_2_, and H_2_O when the undesired paths are irreversible (or nearly irreversible). Therefore, a suitable triaryl borane that exhibits sufficient reactivity toward H_2_ in cooperation with LBs, yet simultaneously avoids the aforementioned irreversible deactivation paths, would be highly desirable. Note that Voicu *et al*. (*36*) successfully applied an FLP comprising **B**^**1**^ and P*^t^*Bu_3_ to the microfluidic separation of ethylene and ethane.

Here, we demonstrate the direct storage of H_2_ in N-heterocyclic compounds such as **H**_**4**_**-Qin** and 2,6-dimethylpiperidine (**H**_**6**_**-Lut**) under various mixed gas conditions including H_2_, CO, CO_2_, and CH_4_ via a shelf-stable **B*^n^***-catalyzed hydrogenation of **Qin** and 2,6-lutidine (**Lut**), respectively. Furthermore, the same **B*^n^*** also catalyzes the dehydrogenation from **H**_**4**_**-Qin** to produce H_2_ with concomitant generation of **Qin**. The molar compositions of H_2_/CO/CO_2_ used in this work (1/1/1, 1/5/1, and 1/1/5) were based on the molar composition of the typical crude H_2_ produced by hydrocarbon resources (H_2_/CO/CO_2_ = 1/1/0.2 to 1/2/0.5) and the typical PSA off-gas (H_2_/CO/CO_2_ = 1/0.1/2), albeit these compositions vary depending on the feedstock (*4*). In addition, these gases include up to 0.9 mmol of H_2_O (table S2), which should be considered under the applied conditions.

## RESULTS

**Fig. 2.**
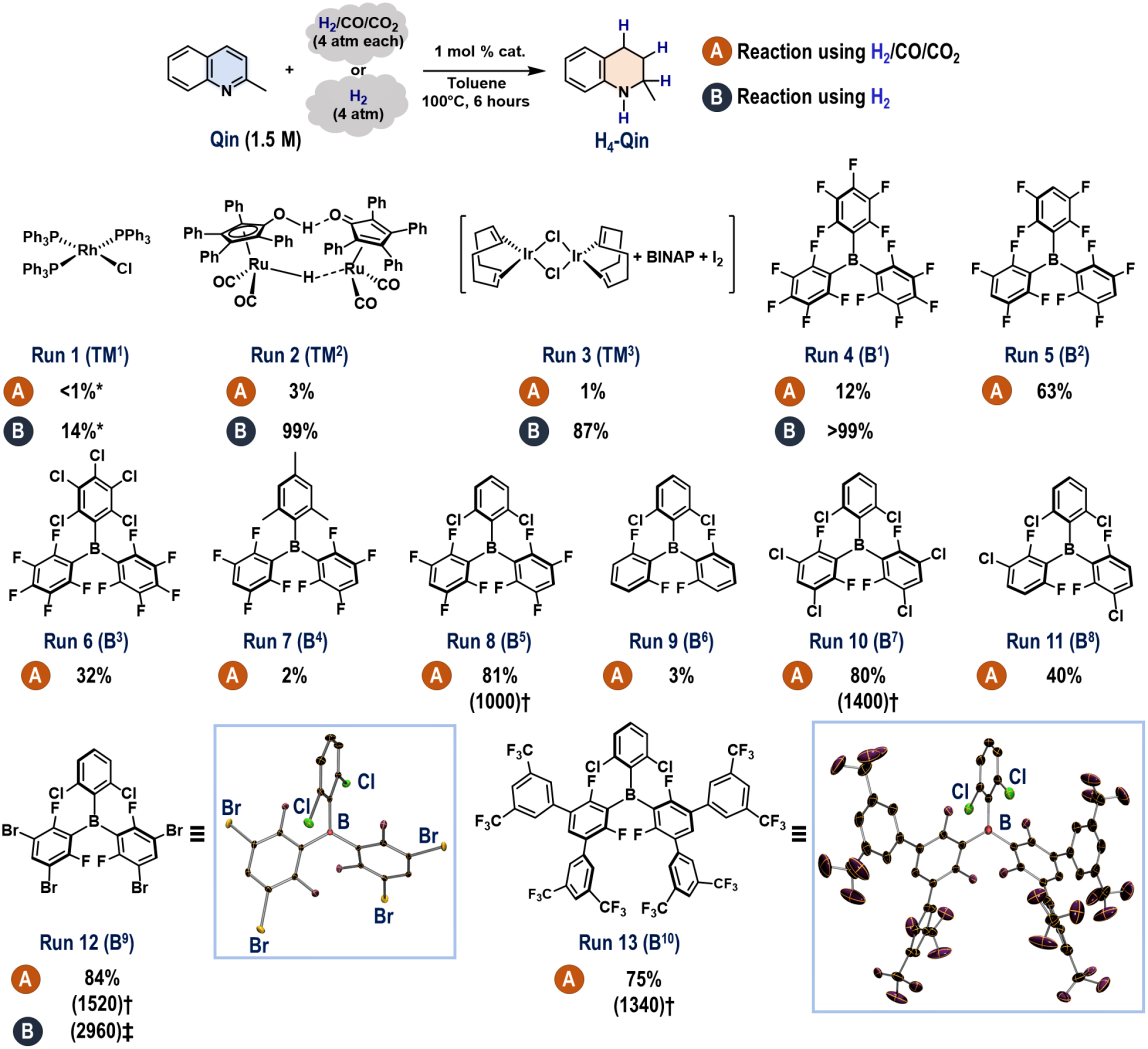
Optimization of the reaction conditions. General conditions for the catalytic hydrogenation of **Qin**: A mixture of **Qin** (2.5 mmol, 1.5 M in toluene) and **B**^**n**^ (1 mol %) was treated with H_2_/CO/CO_2_ (4 atm each; conditions A) or H_2_ (4 atm; conditions B) at 100°C. Yields of **H**_**4**_**-Qin** were determined by GC analysis. The molecular structures of **B**^**9**^ and **B**^**10**^ were determined by single-crystal x-ray diffraction analysis and are shown with thermal ellipsoids at 30% probability (H atoms are omitted for clarity). BINAP, 2,2′-bis(diphenylphosphino)-1,1′-binaphthyl. *denotes 10 mol % catalyst. †denotes catalyst turnover number (TON) after a period of 48 hours under solvent-free conditions using 0.1 mol % **B*^n^*** and H_2_/CO/CO_2_ (30 atm each). ‡denotes catalyst TON after a period of 48 hours under solvent-free conditions using 0.05 mol % **B**^**9**^ and H_2_ (85 atm).

The interconversion between **Qin** and **H**_**4**_**-Qin** was used as a model LOHC system to separate H_2_ under these mixed gas conditions (Fig. 2). As expected, well-established transition metal complexes based on Rh (**TM**^**1**^), Ru (**TM**^**2**^), or Ir (**TM**^**3**^) (*37*) did not catalyze the hydrogenation of **Qin** (1.5 M in toluene) using a gaseous mixture of H_2_/CO/CO_2_ (4 atm each; runs 1 to 3), whereas **H**_**4**_**-Qin** was efficiently yielded when H_2_ (99.95% purity, 4 atm) was used in the cases of **TM**^**2**^ and **TM**^**3**^ (*38*). A higher yield of **H**_**4**_**-Qin** (12%) was observed when 1 mole percent (mol %) **B**^**1**^ was subjected to these mixed gas conditions (run 4A), although the deleterious influence of the contaminants (CO, CO_2_, and/or H_2_O) was again observed compared to the result obtained using pure H_2_ (run 4B). Encouraged by this result, we explored the triaryl boranes **B**^**2**^ to **B**^**6**^, which have been developed by Stephan *et al*. (*39*), Ashley *et al*. (*40*), and Soós *et al*. (*26*, *41*, *42*) (runs 5 to 9). The decreasing Lewis acidity exhibited by the boron centers when the C_6_F_5_ group in **B**^**1**^ were replaced with three para–H-C_6_F_4_ groups (**B**^**2**^) or with two para–H-C_6_F_4_ groups and a 2,6-Cl_2_-C_6_H_3_ group (**B**^**5**^) was found to be critical, and **H**_**4**_**-Qin** was afforded in 63 and 81% yield when **B**^**2**^ and **B**^**5**^ were used, respectively (runs 5 and 8). We thus carried out additional structural modifications via the substitution of the meta-F atoms with respect to the boron atom in **B**^**5**^ with Cl atoms (**B**^**7**^), H and Cl atoms (**B**^**8**^), Br atoms (**B**^**9**^), and (CF_3_)_2_C_6_H_3_ groups (**B**^**10**^) (runs 10 to 13). **B**^**9**^ showed the best result, affording **H**_**4**_**-Qin** in 84% yield even in the presence of CO and CO_2_ (run 12). Moreover, a significant enhancement in the hydrogenation of **Qin** was observed when the reactions were conducted using **B**^**5**^, **B**^**7**^, **B**^**9**^, and **B**^**10**^ in the absence of solvent (fig. S17); **B**^**9**^ exhibited a catalyst turnover number (TON) of 1520 at 100°C in the presence of H_2_/CO/CO_2_ (30 atm each), which is far higher than the TONs obtained using **B**^**5**^ (1000), **B**^**7**^ (1400), or **B**^**10**^ (1340) (runs 8, 10, 12, and 13; see also fig. S18). Note that the TON eventually reached to 2960 when the **B**^**9**^-catalyzed hydrogenation of **Qin** was carried out under the solvent-free conditions including H_2_ (85 atm). The differences observed among **B**^**5**^, **B**^**7**^, **B**^**9**^, and **B**^**10**^ can be rationalized in terms of the electronic and steric properties of the meta-substituents, i.e., their electron-withdrawing ability, which influences the electron-accepting ability of the boron center, and their steric size, which should cause intramolecular steric repulsion among the introduced aryl groups (table S4 and fig. S44) (*43*). In this context, the larger size of the Br atoms in **B**^**9**^ compared to the F (**B**^**5**^) and Cl (**B**^**7**^) atoms can be expected to play a key role in maintaining high activity under the applied mixed gas conditions by destabilizing the four coordinated boron species that would be formed during the reactions involving CO, CO_2_, and/or H_2_O.

Both **B**^**7**^ and **B**^**9**^ exhibited high stability toward air and moisture. **B**^**9**^ can be stored under ambient conditions (22°C, ca. 30% humidity) for at least 1 year without any apparent decomposition, while very minor levels (ca. 1%) of decomposition were observed for **B**^**7**^ after 1 year of storage (figs. S14 and S15).

**Fig. 3.**
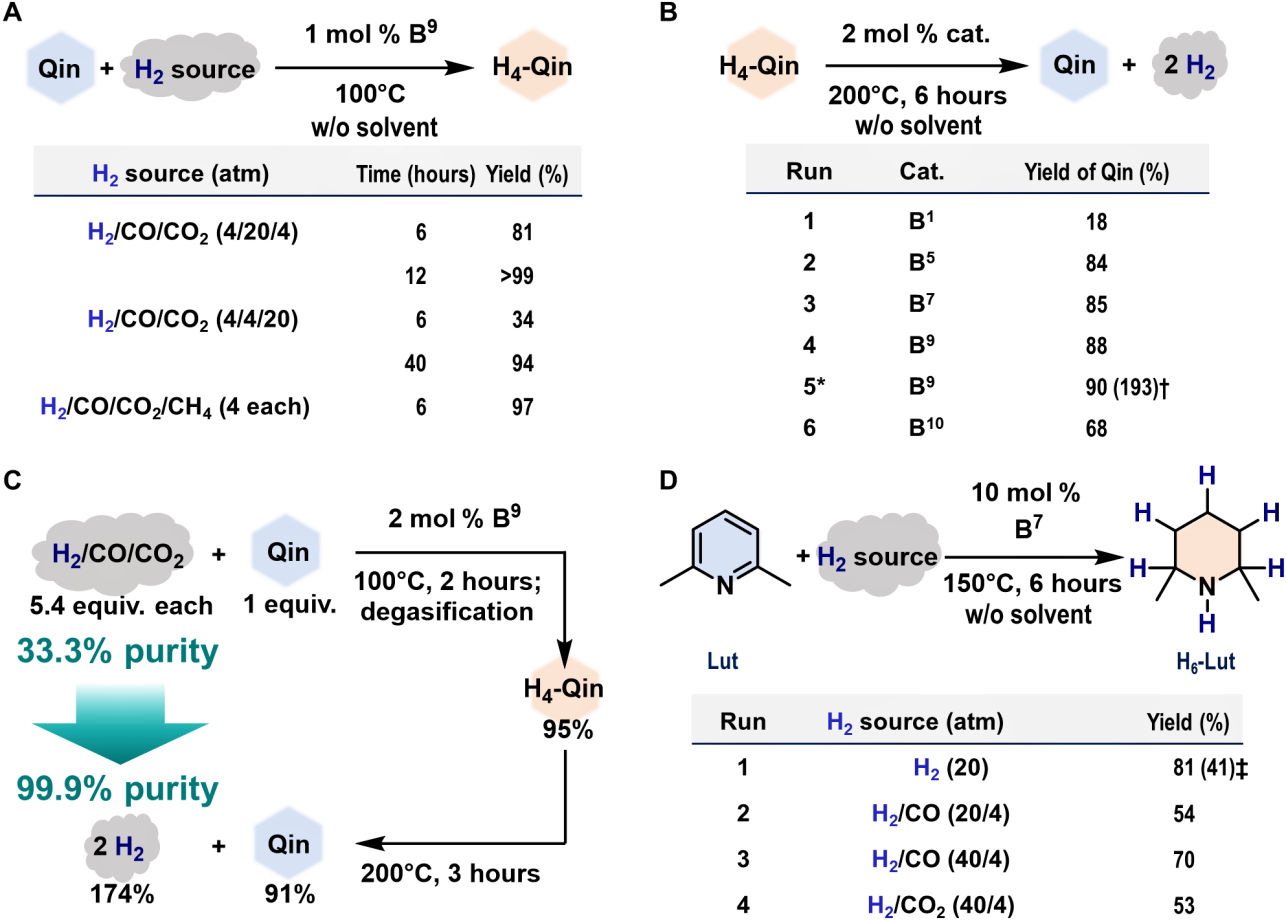
Direct H_2_ purification/storage from contaminated H_2_ gas based on the present catalytic process. (**A**) Exploration of the gas composition of the H_2_ source. Yields of **H**_**4**_**-Qin** were determined using GC analysis. (**B**) Catalytic dehydrogenation of **H**_**4**_**-Qin** (3.8 mmol) to **Qin** in the absence of solvent. Yields of **Qin** were determined by GC analysis. *denotes 2 hours. †denotes yield of recovered H_2_ based on the collected volume (*V* = 164 ml, 7.32 mmol). (**C**) H_2_ purification based on the **B**^**9**^-catalyzed hydrogenation of **Qin** (1.0 mmol) under mixed gas conditions and subsequent catalytic dehydrogenation. Yield of recovered H_2_ based on the collected volume (*V* = 39.0 ml, 1.74 mmol); H_2_ purity (%) = (molar amount of H_2_)/(sum of the molar amounts of H_2_, CO, and CO_2_) × 100. (**D**) **B**^**7**^-catalyzed hydrogenation of **Lut** using a variety of H_2_ sources that were dried over 4-Å MS before use. ‡indicates that **B**^**9**^ was used.

The **B**^**9**^-catalyzed hydrogenation of **Qin** (1 mol % catalyst, without solvent) also proceeded to furnish **H**_**4**_**-Qin** in >99 and 94% yield using CO-rich (H_2_/CO/CO_2_ = 4/20/4 atm; a model of syngas) and CO_2_-rich (H_2_/CO/CO_2_ = 4/4/20 atm; a model of industrial off-gas) mixtures, respectively, although a longer reaction time was required in both cases (Fig. 3A). These results imply that CO and CO_2_ kinetically affect the catalytic activity of **B**^**9**^ toward hydrogenation, with obvious suppression when an excess of the contaminant CO_2_ with respect to H_2_ is present. The coexistence of CH_4_ did not hamper the progress of the reaction.

We also explored the optimal conditions for the catalytic dehydrogenation of **H**_**4**_-**Qin** to afford **Qin** (Fig. 3B) (*44*, *45*). Without solvent, 2 mol % **B**^**5**^, **B**^**7**^, and **B**^**9**^ successfully catalyzed the production of **Qin** in 84 to 88% yields at 200°C for 6 hours, whereas **B**^**1**^ and **B**^**10**^ exhibited inferior results (18 and 68%, respectively) under otherwise identical reaction conditions. The recovery of H_2_ [193% yield; CO and CO_2_ were not detected by gas chromatography (GC) analysis; fig. S20] was confirmed during the **B**^**9**^-catalyzed dehydrogenation of **H**_**4**_-**Qin** (2 hours) to **Qin** in 90% yield. Under the applied reaction conditions, the hydrogenation of **Qin** and the dehydrogenation of **H**_**4**_**-Qin** could be catalyzed simultaneously by **B**^**9**^. Further optimization of the reaction equipment so that the recovered H_2_ can be efficiently removed could thus be expected to increase the dehydrogenation efficiency.

To develop a strategy for the purification of the contaminated H_2_, we designed a reaction system based on a **B**^**9**^-catalyzed hydrogenation/dehydrogenation sequence starting from H_2_/CO/CO_2_ [1/1/1 molar ratio; H_2_ purity = (molar amount of H_2_)/(sum of the molar amounts of H_2_, CO, and CO_2_) × 100 = 33.3%] as a feed gas (Fig. 3C). In the presence of 2 mol % **B**^**9**^, H_2_ was directly stored in **H**_**4**_**-Qin** (0.95 mmol, 95%) from H_2_/CO/CO_2_ (5.4 equivalents each) via the hydrogenation of **Qin** (1.0 mmol). After a simple evacuation, dehydrogenation of the obtained **H**_**4**_**-Qin** was carried out to generate H_2_ (1.74 mmol, 174%) with a concomitant regeneration of **Qin** (0.91 mmol, 91%). Thus, a significant increase in the H_2_ purity from 33.3 to 99.9% was demonstrated by the efficient removal of CO (not detected by GC analysis) and CO_2_ (detected in ca. 0.1%; fig. S21) via a single cycle of the **B**^**9**^-catalyzed hydrogenation/dehydrogenation sequence. The complete removal of CO in a single cycle would be especially noteworthy, as the removal of CO remains challenging in the well-developed multistep, multibed PSA and membrane technologies (*4*).

We further explored the catalytic activity of **B**^**7**^ and **B**^**9**^ toward the hydrogenation of **Lut** to afford **H**_**6**_**-Lut** under the mixed gas conditions (Fig. 3D). This further investigation revealed that the H_2_ storage capacity could be increased from 2.7 (**H**_**4**_**-Qin**) to 5.3 wt % (**H**_**6**_**-Lut**). Note that **Lut** has been a challenging substrate in previously reported organoborane-catalyzed hydrogenations using H_2_ even under diluted conditions (*46*, *47*). In the presence of **B**^**7**^ (10 mol %) and the absence of solvent, **H**_**6**_**-Lut** was formed in 81% yield using H_2_ [20 atm; dried over 4-Å molecular sieves (MSs) before use], while a decrease in yield was observed for **B**^**9**^ (run 1). Without the dehydration of H_2_, the hydrogenation of **Lut** also proceeded to afford **H**_**6**_**-Lut** in 72% under identical conditions (fig. S22). Moreover, **B**^**7**^ exhibited promising results for the simultaneous separation and storage of H_2_ in **H**_**6**_**-Lut** from CO- and CO_2_-contaminated H_2_ gas (runs 2 to 4), albeit an excess of H_2_ with respect to the contaminants was present.

**Fig. 4.**
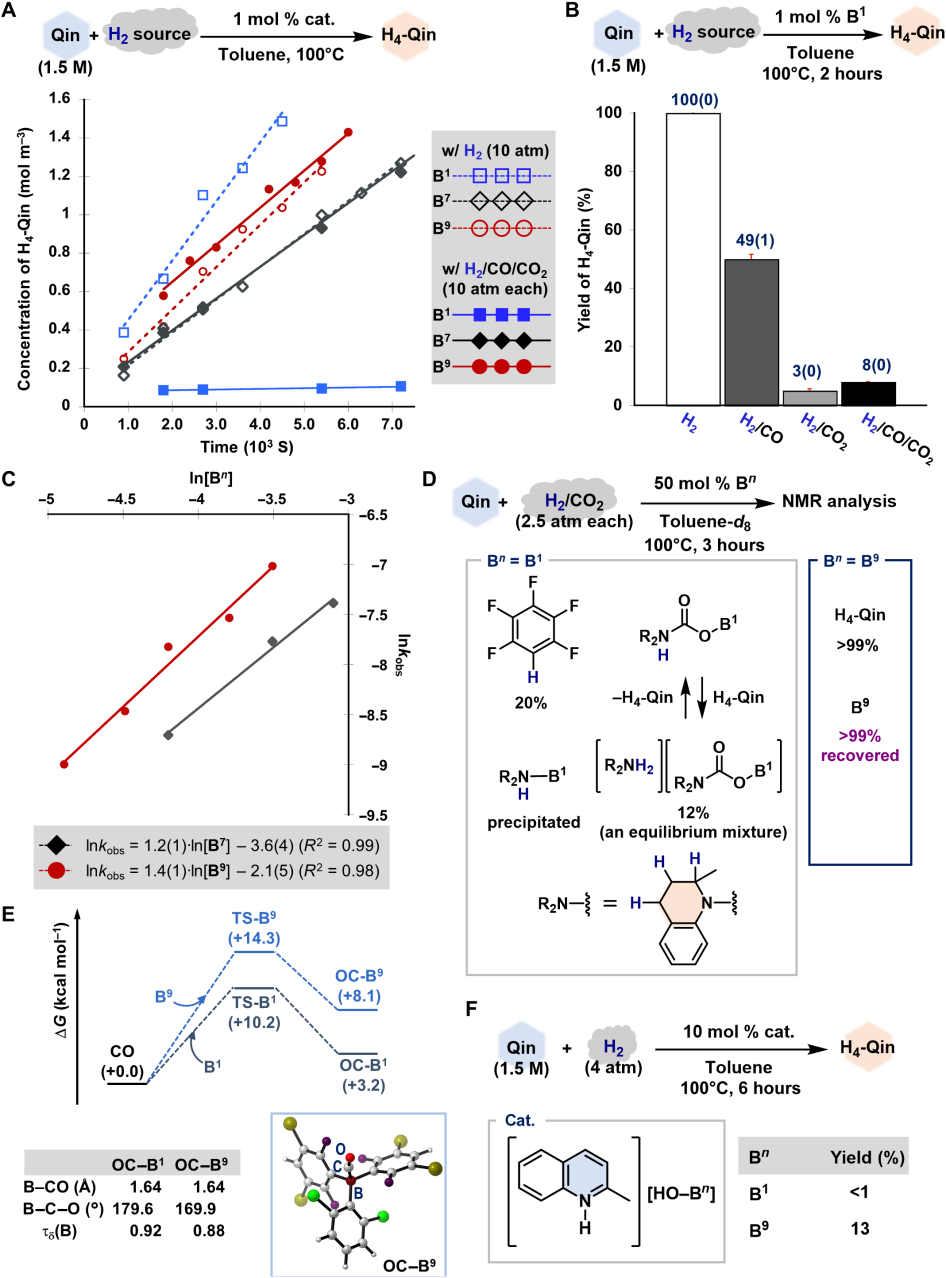
Mechanistic experiments. (**A**) Kinetic profiles of the concentration of **H**_**4**_**-Qin** (mole meter^−3^) with respect to reaction time (10^3^ s) obtained from the hydrogenation of **Qin** (1.5 M in toluene) in the presence of **B*n*** (*n* = 1, 7, and 9) and different H_2_ sources (pure H_2_ or H_2_/CO/CO_2_; 10 atm each). (**B**) Influence of the gas composition on the **B**^**1**^-catalyzed hydrogenation of **Qin**. Each experiment was pressurized with H_2_ (10 atm) and/or CO*_x_* (10 atm; *x* = 1 and/or 2). Average yields of independent three runs are shown with SEs. (**C**) Profile of ln*k*_obs_ with respect to ln[**B*n***] (*n* = 7 and 9). (**D**) Detailed analysis of the **B*n***-catalyzed hydrogenation of **Qin** (*n* = 1 and 9) in the presence of H_2_/CO_2_ (2.5 atm each). Product yields were calculated using ^19^F NMR analysis with C_6_H_5_CF_3_ as the internal standard. In the case of **B**^**1**^, several unidentified resonances were observed (for details, see fig. S38). (**E**) Calculated free energy profiles for the formation of **OC**─**B*n*** (*n* = 1 and 9) [kilocalorie mole^−1^; ωB97X-D/6-311+G(d,p)//ωB97X-D/6-31G(d,p) level]. The gas phase–optimized structure of **OC**─**B**^**9**^ and selected structural parameters for **OC**─**B*n*** (*n* = 1 and 9) are also shown. (**F**) Hydrogenation of **Qin** using [**Qin**─H][HO─**B*n***] (*n* = 1 and 9). Yields of **H**_**4**_**-Qin** were determined via GC analysis.

To gain insight into the reaction mechanism for the present hydrogenation of N-heteroaromatic compounds in the presence of CO, CO_2_, and H_2_O, preliminary mechanistic studies were conducted using **Qin** (Fig. 4). First, we monitored the progress of the conversion of **Qin** to **H**_**4**_**-Qin** using **B**^**1**^, **B**^**7**^, or **B**^**9**^ under each condition using solely H_2_ or H_2_/CO/CO_2_ (Fig. 4A). The production of **H**_**4**_**-Qin** exhibited a zeroth-order dependence on the concentration of **Qin** with rate constants (*k*_obs_) as follows: 3.08(29) × 10^−4^ mol m^−3^ s^−1^ (H_2_) and 3.56(60) × 10^−6^ m^−3^ s^−1^ (H_2_/CO/CO_2_) (**B**^**1**^); 1.71(6) × 10^−4^ m^−3^ s^−1^ (H_2_) and 1.66(5) × 10^−4^ m^−3^ s^−1^ (H_2_/CO/CO_2_) (**B**^**7**^); and 2.21(13) × 10^−4^ m^−3^ s^−1^ (H_2_) and 1.93(10) × 10^−4^ m^−3^ s^−1^ (H_2_/CO/CO_2_) (**B**^**9**^). Moreover, these results suggest that **H**_**4**_**-Qin** itself does not affect the rate of hydrogenation, as neither an increase nor decrease in the rate was observed increasing conversion to **H**_**4**_**-Qin**. Thus, the influence of CO and/or CO_2_ is almost negligible for the **B**^**7**^- and **B**^**9**^-catalyzed hydrogenation processes, at least under conditions that do not involve excess amounts of CO/CO_2_ with respect to H_2_ (vide supra). In stark contrast, the **B**^**1**^-catalyzed process was significantly inhibited in the presence of CO and/or CO_2_. Control experiments using H_2_/CO (10 atm each) and H_2_/CO_2_ (10 atm each) clarified that both CO and CO_2_ affect the catalytic activity of **B**^**1**^ and that contamination with CO_2_ is especially deleterious (Fig. 4B). We also confirmed the kinetic orders in catalyst **B**^**7**^ [1.2(1)] and **B**^**9**^ [1.4(1)] under the H_2_/CO/CO_2_ atmosphere, demonstrating that these triaryl boranes do catalyze the formation of **H**_**4**_**-Qin** (Fig. 4C).

Next, the influence of CO_2_, CO, and H_2_O was investigated using **B**^**1**^ and **B**^**9**^. The hydrogenation of **Qin** was carried out in the presence of each borane (50 mol %) using H_2_/CO_2_ (2.5 atm each) at 100°C in toluene-*d*_8_ and analyzed using multinuclear nuclear magnetic resonance (NMR) spectroscopy. In the case of **B**^**1**^, the resultant mixture included C_6_F_5_H (20%), an equilibrium mixture of [**H**_**4**_**-Qin**─COO**B**^**1**^] and [**H**_**4**_**-Qin**─H][**H**_**3**_**-Qin**─COO**B**^**1**^] (12%) (*28*, *29*), and several unidentified compounds (Fig. 4D and fig. S38). Precipitation of the nitrogen-boron adduct [**H**_**4**_**-Qin**─**B**^**1**^] was also confirmed. We separately confirmed that C_6_F_5_H was not formed in the absence of CO_2_ (fig. S41). These results clarify that CO_2_ triggers the irreversible decomposition of **B**^**1**^ to yield C_6_F_5_H via protodeboronation from both [**H**_**4**_**-Qin**─COO**B**^**1**^] and [**H**_**4**_**-Qin**─H][**H**_**3**_**-Qin**─COO**B**^**1**^] under heating conditions. In stark contrast, when **B**^**9**^ was used, the generation of **H**_**4**_**-Qin** and recovery of **B**^**9**^ in >99% yields were observed under otherwise identical conditions. These results are consistent with the fact that the **B**^**9**^-catalyzed hydrogenation of **Qin** is not irreversibly inhibited by CO_2_.

Density functional theory calculations were carried out at the ωB97X-D/6-311+G(d,p)//ωB97X-D/6-31G(d,p)//gas phase level of theory to shed light on the observed kinetic suppression of the hydrogenation of **Qin** by CO (*48*). The relative Gibbs energies (kilocalorie mole^−1^) for **OC─B*n*** (Lewis pairs comprising CO and **B*n*** and **TS─B*n*** (saddle point species) with respect to [CO + **B*n***] (*n* = 1 and 9) are shown in Fig. 4E. The coordination of CO to the boron atoms in both **B**^**1**^ and **B**^**9**^ is an endothermic process (*29*, *33*), and coordination to the latter is far less favorable from a kinetic and thermodynamic perspective. The standard Gibbs free energies for the formation of **OC─B*n*** are +3.2 (*n* = 1) and +8.1 (*n* = 9) kcal mol^−1^, and the activation energies to overcome **TS─B*n*** are +10.2 (*n* = 1) and +14.3 (*n* = 9) kcal mol^−1^. These results rationalize the experimental results, i.e., the observations that contamination with excess CO kinetically affects both the **B**^**1**^- and **B**^**9**^-catalyzed hydrogenation of **Qin** under the applied conditions, with this suppression being significant in the former case. The differences in the stability of **OC─B*n*** should be related to the degree of geometric deviation from the ideal tetrahedral geometry around their boron centers, which can be evaluated on the basis of the value of τ_δ_(B) [τ_δ_ = {360 – (α + β)/141 × β/α}, where α and β are the largest and second largest C**─**B**─**C angles obtained from the gas phase–optimized structures of **OC─B*n***] (*49*). More efficient orbital overlap between the lone pair on the carbon atom in CO and the p orbital on the boron atom in **B*n*** should result in higher stabilization of **OC─B*n*** adducts, adopting a more ideal tetrahedral geometry [τ_δ_(B) = 0.9 to 1.0] and a linear arrangement of the B**─**C**─**O atoms (∡B**─**C**─**O ≈ 180°). In the present study, the lower τ_δ_(B) of 0.88 for **OC─B**^**9**^ indicates that its boron atom adopts a more distorted tetrahedral geometry compared to that of **OC─B**^**1**^ [τ_δ_(B) = 0.92], and the B**─**C**─**O atoms in **OC─B**^**9**^ are confirmed to exhibit a bent alignment (169.9° versus 179.6° in **OC─B**^**1**^). These results thus demonstrate the effective destabilization of **OC─B**^**9**^ due to the increased steric repulsion between CO and the 2,6-Cl2-C6H3 group introduced on **B**^**9**^, which eventually results in the reduced impact of CO on the **B**^**9**^ hydrogenation of **Qin**.

We further evaluated the influence of H_2_O on the hydrogenation (Fig. 4F). GC analysis confirmed that no conversion of **Qin** occurred in the presence of H_2_ (4 atm) at 100°C when 10 mol % [**Qin**─H][HO─**B**^**1**^] was used. Although **H**_**4**_**-Qin** was furnished in 13% yield when 10 mol % [**Qin**─H][HO─**B**^**9**^] was used, the low yield again confirmed the deleterious influence of H_2_O. On the basis of these results and the stability of **B**^**9**^ toward moisture at ambient conditions (vide supra), [**Qin**─H][HO─**B**^**9**^] was not generated under the applied conditions shown in Figs. 2 and 3, although H_2_O might be present as a contaminant.

## DISCUSSION

The present results demonstrate a proof of concept for a H_2_ purification technology based on LOHCs that goes beyond their well-established use in H_2_ storage. This technology can be expected to change the industrial value of crude H_2_ containing substantial amounts of CO, CO_2_, and CH_4_, which can be produced from a variety of carbon resources such as biomass and industrial off-gases. The operational simplicity of the present method should allow the construction of combined processes involving PSA and/or membranes. Moreover, this work demonstrates a new aspect of main group catalysis beyond its application as a simple alternative to well-established transition metal–catalyzed processes, i.e., the main group–catalyzed hydrogenation of unsaturated molecules under mixed gas conditions.

## MATERIALS AND METHODS

### General considerations

Unless otherwise noted, all manipulations were conducted under a nitrogen atmosphere using standard Schlenk line or grove box techniques. MSs (4 Å) were activated by heating with a heat gun in vacuo (ca. 0.2 mmHg) for 5 min. ^1^H, ^11^B, ^13^C, ^19^F, and ^31^P NMR spectra were recorded on Bruker Avance III 400 or JEOL JNM-400 spectrometers at 25°C. The chemical shifts in the ^1^H NMR spectra were recorded relative to Me_4_Si or residual protonated solvent [C_6_D_5_H (δ 7.16), CHCl_3_ (δ 7.26), C_7_D_7_H (δ 2.08), and CDHCl_2_ (δ 5.32)]. The chemical shifts in the ^11^B NMR spectra were recorded relative to BF_3_. The chemical shifts in the ^13^C NMR spectra were recorded relative to Me_4_Si or deuterated solvent [C_6_D_6_ (δ 128.06), CDCl_3_ (δ 77.16), and CD_2_Cl_2_ (δ 53.84)]. The chemical shifts in the ^19^F NMR spectra were recorded relative to α,α,α-trifluorotoluene [δ −65.64]. The chemical shifts in the ^31^P NMR spectra were recorded relative to 85% H_3_PO_4_ as an external standard. Assignment of the resonances in ^1^H and ^13^C NMR spectra was based on ^1^H-^1^H correlation spectroscopy, heteronuclear multiple-quantum coherence, and/or heteronuclear multiple-bond correlation experiments. High-resolution mass spectrometry was performed at the Instrumental Analysis Center, Faculty of Engineering, Osaka University. A single-crystal x-ray diffraction analysis was carried out using the Rigaku XtaLAB Synergy equipped with the HyPix-6000HE detector. Analytical GC was carried out on a Shimadzu GC-2025 gas chromatograph, equipped with a flame ionization detector, or a Shimadzu GC-2010 gas chromatograph, equipped with a barrier discharge ionization detector.

### Materials

All commercially available reagents including superdehydrated solvents (*n*-hexane, toluene, tetrahydrofuran, and diethyl ether) were used as received. Benzene-*d*_6_ and toluene-*d*_8_ were distilled from sodium benzophenone ketyl before use. CDCl_3_ and CD_2_Cl_2_ were stored inside the grove box over MSs (4 Å) after several freeze-pump-thaw cycles. **Qin**, **H**_**4**_**-Qin**, and **Lut** were used after distillation over CaH_2_. Triaryl boranes (**B**^**2**^ to **B**^**6**^), (*26*, *39*–*42*) potassium (2,6-dichlorophenyl)trifluoroborate, (*41*) 1,5-dichloro-2,4-difluoro-3-iodobenzene (*50*), 1-chloro-2,4-difluoro-3-iodobenzene (*50*), and 1,5-dibromo-2,4-difluoro-3-iodobenzene (*50*) were prepared by following the reported procedures. Gaseous chemicals including H_2_, CO, CO_2_, CH_4_, H_2_/CO (a 1/1 molar ratio), H_2_/CO_2_ (a 1/1 molar ratio), and H_2_/CO/CO_2_ (a 1/1/1 molar ratio) were purchased and used as received otherwise noted.

### Synthesis of B^9^

A solution of 1,5-dibromo-2,4-difluoro-3-iodobenzene (3.45 g, 8.67 mmol, 0.29 M in Et_2_O) was slowly treated with *^i^*PrMgCl (8.7 ml, 8.7 mmol, 1.0 M in Et_2_O). After stirring at room temperature for 1 hour, the resultant solution was transferred into a suspension of potassium (2,6-dichlorophenyl)trifluoroborate (1.05 g, 4.16 mmol, 0.42 M in Et_2_O) at 0°C. The reaction mixture was then allowed to warm to room temperature, where it was stirred for another 14 hours. After the removal of all volatiles in vacuo, the residue was extracted with α,α,α-trifluorotoluene (50 ml for three times; warmed to 70°C before use). The combined organic layers were concentrated in vacuo and washed with hexane (cooled to −20°C before use) to afford **B**^**9**^ as a white solid (2.56 g, 3.66 mmol, 88%).

### H_2_ purification from H_2_/CO/CO_2_ via a B^9^-catalyzed hydrogenation/dehydrogenation sequence

A 30-ml autoclave was charged with **Qin** (145 mg, 1.01 mmol), **B**^**9**^ (13.9 mg, 0.199 mmol), and tetradecane (59.5 mg; internal standard). Once sealed, the autoclave was pressurized with H_2_/CO/CO_2_ (4 atm each; 5.4 mmol each) and heated to 100°C for 2 hours. After cooling to room temperature, all volatiles were removed in vacuo, and GC analysis showed the production of **H**_**4**_**-Qin** in 95%. Then, the reaction mixture was transferred into a 10-ml two-neck flask. During this manipulation, the residue inside the autoclave reactor was extracted with toluene to minimize the loss of reagents. The toluene was then removed in vacuo; however, this extraction step is not essential for the following dehydrogenation. The reaction mixture was then stirred at 200°C for 3 hours. The volume of collected gas was measured using a graduated cylinder to calculate the yield of H_2_ (39 ml, 1.7 mmol). The conversion of **H**_**4**_**-Qin** (94%), the yield of **Qin** (91%), and the purity of the collected H_2_ gas were determined using GC analysis.

## Supplementary Materials

### This PDF file includes:

Supplementary Text Figs. S1 to S44 Tables S1 to S32 NMR spectra References
