# Supplementary material for: Main group catalysis for H2 purification based on liquid organic hydrogen carriers
Source: Sci Adv. 2022 Oct 26;8(43):eade0189. doi: 10.1126/sciadv.ade0189 (PMC9604535; doi:10.1126/sciadv.ade0189)
Supplement: Supplementary file 1 — Supplementary Text Figs. S1 to S44 Tables S1 to S32 NMR spectra References [file sciadv.ade0189_sm.pdf]

Science Advances
AAAS

Supplementary Materials for

Main group catalysis for H_2 purification based on liquid organic hydrogen carriers

Taiki Hashimoto *et al.*

Corresponding author: Sensuke Ogoshi, ogoshi@chem.eng.osaka-u.ac.jp;
Yoichi Hoshimoto, hoshimoto@chem.eng.osaka-u.ac.jp

Sci. Adv. **8**, eade0189 (2022)
DOI: 10.1126/sciadv.ade0189

This PDF file includes:

Supplementary Text
Figs. S1 to S44
Tables S1 to S32
NMR spectra
References

[1] General considerations

Unless otherwise noted, all manipulations were conducted under a nitrogen atmosphere using standard Schlenk line or grove box (GB) techniques. Molecular sieves (4 Å) were activated by heating with a heat gun *in vacuo* (ca. 0.2 mmHg) for 5 min. ^{1}H, ^{11}B, ^{13}C, ^{19}F, and ^{31}P NMR spectra were recorded on Bruker AVANCE III 400 or JEOL JNM-400 spectrometers at 25 °C. The chemical shifts in the ^{1}H NMR spectra were recorded relative to Me_4Si or residual protonated solvent (C_6D_5H (δ 7.16), $CHCl_3$ (δ 7.26), C_7D_7H (δ 2.08), $CDHCl_2$ (δ 5.32)). The chemical shifts in the ^{11}B NMR spectra were recorded relative to BF_3. The chemical shifts in the ^{13}C NMR spectra were recorded relative to Me_4Si or deuterated solvent (C_6D_6 (δ 128.06), $CDCl_3$ (δ 77.16), CD_2Cl_2 (δ 53.84)). The chemical shifts in the ^{19}F NMR spectra were recorded relative to α,α,α-trifluorotoluene (δ −65.64). The chemical shifts in the ^{31}P NMR spectra were recorded relative to 85% H_3PO_4 as an external standard. Assignment of the resonances in ^{1}H and ^{13}C NMR spectra was based on ^{1}H–^{1}H COSY, HMQC, and/or HMBC experiments. High resolution mass spectrometry (HRMS) was performed at the Instrumental Analysis Center, Faculty of Engineering, Osaka University. A single-crystal X-ray diffraction analysis was carried out using the Rigaku XtaLAB Synergy equipping with the HyPix-6000HE detector. Analytical gas chromatography (GC) was carried out on a Shimadzu GC-2025 gas chromatograph, equipped with a flame ionization detector, or a Shimadzu GC-2010 gas chromatograph, equipped with a barrier discharge ionization detector.

[2] Materials

All commercially available reagents including super-dehydrated solvents (*n*-hexane, toluene, tetrahydrofuran, and diethyl ether) were purchased from Sigma Aldrich, Tokyo Chemical Industry (TCI) and FUJIFILM Wako Pure Chemical Corporation, and used as received. Benzene-d_6 and toluene-d_8 were distilled from sodium benzophenone ketyl prior to use. $CDCl_3$ and CD_2Cl_2 were stored inside the GB over molecular sieves (4 Å) after several freeze-pump-thaw cycles. 2-Methylquinoline (**Qin**), 1,2,3,4-tetrahydro-2-methylquinoline (**H_4-Qin**), and 2,6-lutidine (**Lut**) were purchased from TCI, and used after distillation over CaH_2. Triaryl boranes (**B^2**, **B^3**, **B^4**, **B^5**, and **B^6**),[26,39-42] potassium (2,6-dichlorophenyl)trifluoroborate,[41] 1,5-dichloro-2,4-difluoro-3-iodobenzene,[50] 1-chloro-2,4-difluoro-3-iodobenzene,[50] and 1,5-dibromo-2,4-difluoro-3-iodobenzene[50] were prepared by following the reported procedures.

Gaseous chemicals including H_2, CO, CO_2, CH_4, H_2/CO (a 1:1 molar ratio), H_2/CO_2 (a 1:1 molar ratio), and H_2/CO/CO_2 (a 1:1:1 molar ratio) were purchased from Sumitomo Seika Chemicals Company, and used as received otherwise noted. Note that these gases include some impurities as shown in Table S1.

	Impurity				
	N_2	O_2	CO	CO_2	H_2
H_2	<200	<50	<1	<1	-
CO	<500	<100	-	<100	<100
CH_4	<5000	<500	-	<5000	-

Table S1. Impurities contaminated in H_2, CO, and CH_4 (shown in ppm).

Metrical data for the solid-state structures are available from Cambridge Crystallographic Data Centre: CCDC2162155 (**B^7**), 2162156 (**B^9**), 2162157 (**B^{10}**), 2162153 ([**Qin**–H][HO–**B^1**]), 2162154 ([**Qin**–H][HO–**B^9**]), 2164165 (**H_4-Qin**–**B^1**).

[3] Synthesis of B^7

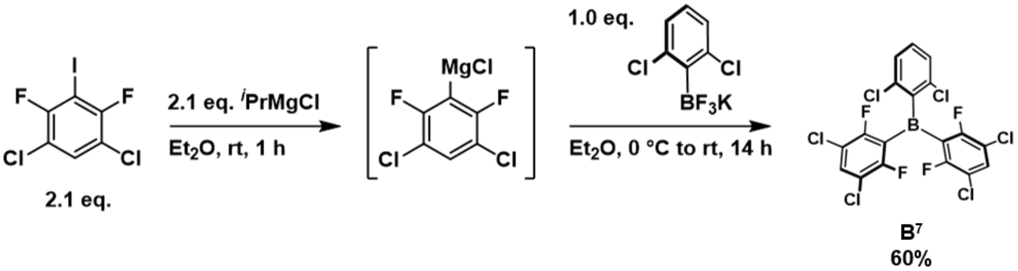

Fig S1. Synthesis of B^7.

A solution of 1,5-dichloro-2,4-difluoro-3-iodobenzene (3.01 g, 9.75 mmol, 0.32 M in Et_2O) was slowly treated with ^{i}PrMgCl (4.9 mL, 9.8 mmol, 2.0 M in Et_2O). After stirring at room temperature for 1 h, the resultant solution was transferred into a suspension of potassium (2,6-dichlorophenyl)trifluoroborate (1.17 g, 4.63 mmol, 0.93 M in Et_2O) at 0 °C. The reaction mixture was then allowed to warm to room temperature, where it was stirred for another 14 h. After the removal of all volatiles *in vacuo*, the residue was extracted with hexane (50 mL × 5; warmed to 70 °C prior to use). The combined organic layer was concentrated *in vacuo* and washed with hexane (cooled to −20 °C prior to use) to afford $\mathbf{B^7}$ as a white solid (1.46 g, 2.80 mmol, 60%). A single crystal suitable for X-ray diffraction analysis was prepared by recrystallization from toluene/hexane at −35 °C. ^{1}**H NMR** (400 MHz, C_6D_6): δ 6.80 (t, $^4J_{H,F}$ = 7.5 Hz, 2H); 6.76 (d, J = 8.0Hz, 2H), 6.46 (t, J = 8.0 Hz, 1H). ^{11}**B NMR** (128 MHz, C_6D_6): δ 64.6 (br). ^{13}**C{^{1}H} NMR** (101 MHz, C_6D_6): δ 159.1 (dd, J = 10.2 Hz, $^1J_{C,F}$ = 256.2 Hz), 141.1 (dm), 136.3, 134.8, 131.7, 127.2, 118.9 (dm), 117.9 (d, J = 22.2 Hz). ^{19}**F NMR** (376 MHz, C_6D_6): δ −104.0 (d, $^4J_{H,F}$ = 7.5 Hz, 4F). X-ray data for $\mathbf{B^7}$ (Fig S2): M = 520.73, colorless, orthorhombic, *Pbcn* (#60), a = 16.6550(3) Å, b = 10.3237(2) Å, c = 11.4217(2) Å, α = 90°, β = 90°, γ = 90°, V = 1963.86(6) Å^3, Z = 4, *Dcalcd* = 1.761 g/cm^3, T = −130 °C, R_1 (wR_2) = 0.0252 (0.0641).

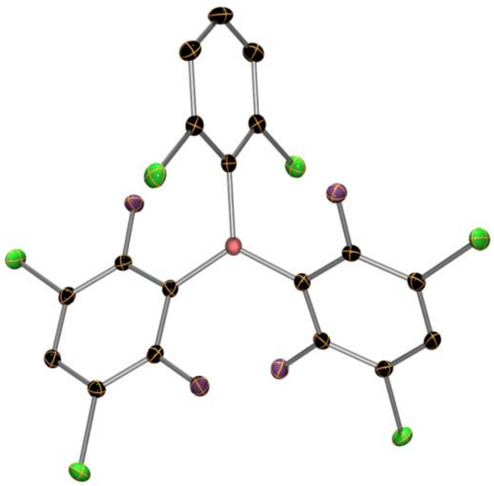

Fig S2. Molecular structure of B^7 with ellipsoids set at 30% probability. H atoms are omitted for clarity.

[4] Synthesis of B[8]

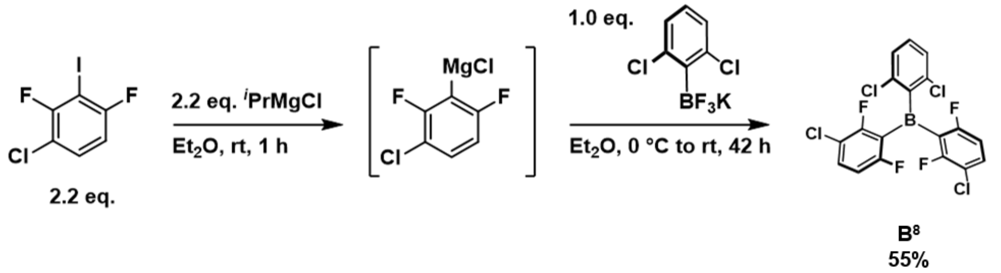

Fig S3. Synthesis of B[8].

A solution of 1-chloro-2,4-difluoro-3-iodobenzene (3.02 g, 11.0 mmol, 0.33 M in Et_2O) was slowly treated with ^{i}PrMgCl (11.0 mL, 11.0 mmol, 1.0 M in Et_2O). After stirring at room temperature for 1 h, the resultant solution was transferred into a suspension of potassium (2,6-dichlorophenyl)trifluoroborate (1.24 g, 4.90 mmol, 0.49 M in Et_2O) at 0 °C. The reaction mixture was then allowed to warm to room temperature, where it was stirred for another 42 h. After the removal of all volatiles *in vacuo*, the residue was extracted with α,α,α-trifluorotoluene (50 mL × 3; warmed to 70 °C prior to use). The combined organic layer was concentrated *in vacuo* and washed with hexane (cooled to −20 °C prior to use) to afford **B[8]** as a white solid (1.21 g, 2.68 mmol, 55%). **^{1}H NMR** (400 MHz, C_6D_6): δ 6.86 (d, J = 8.4 Hz, 2H), 6.81 (dt, J = 6.0 Hz, J = 8.5 Hz, 2H), 6.57 (t, J = 8.4 Hz, 1H), 6.19 (t, J =9.0 Hz, 2H). **^{11}B NMR** (128 MHz, C_6D_6): δ 62.8 (br). **^{13}C{^{1}H} NMR** (101 MHz, C_6D_6): δ 164.3 (dd, J = 9.6 Hz, $^{1}J_{C,F}$ = 256.1 Hz), 160.8 (dd, J = 11.1 Hz, $^{1}J_{C,F}$ = 257.1 Hz), 142.3 (dm), 136.4 (d, J = 11.6 Hz), 134.9, 131.2, 127.1, 118.6 (dm), 117.2 (dd, J = 3.8 Hz, J = 21.2 Hz), 112.6 (dd, J = 3.8 Hz, J = 27.0 Hz). **^{19}F NMR** (376 MHz, C_6D_6): δ −102.0 (d, J = 11.3 Hz, 2F), −102.2 (br, 2F).

[5] Synthesis of B[9]

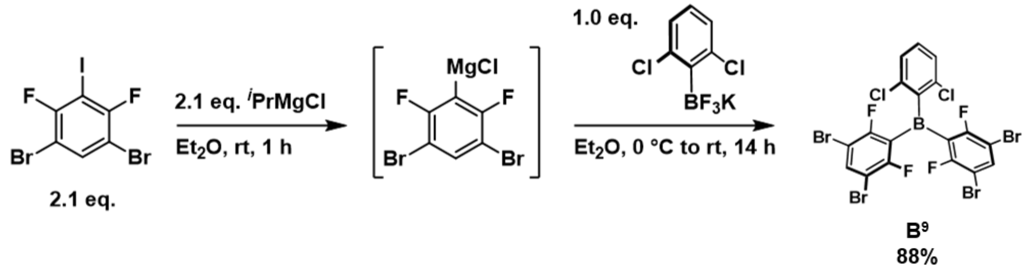

Fig S4. Synthesis of B[9].

A solution of 1,5-dibromo-2,4-difluoro-3-iodobenzene (3.45 g, 8.67 mmol, 0.29 M in Et_2O) was slowly treated with ^{i}PrMgCl (8.7 mL, 8.7 mmol, 1.0 M in Et_2O). After stirring at room temperature for 1 h, the resultant solution was trnsferred into a suspension of potassium (2,6-dichlorophenyl)trifluoroborate (1.05 g, 4.15 mmol, 0.42 M in Et_2O) at 0 °C. The reaction mixture was then allowed to warm to room temperature, where it was stirred for another 14 h. After the removal of all volatiles *in vacuo*, the residue was extracted with α,α,α-trifluorotoluene (50 mL × 3; warmed to 70 °C prior to use). The combined organic layer was concentrated *in vacuo* and washed with hexane (cooled to −20 °C prior to use) to afford **B[9]** as a white solid (2.56 g, 3.66 mmol, 88%). A single crystal suitable for X-ray diffraction analysis was prepared by recrystallization from toluene/hexane at −35 °C. **^{1}H NMR** (400 MHz, C_6D_6): δ 7.20 (t, $^{4}J_{H,F}$ = 7.2 Hz,

2H), 6.74 (d, J = 8.0 Hz, 2H), 6.45 (t, J = 8.0 Hz, 1H). **^{11}B NMR** (128 MHz, C_6D_6): δ 60.5 (br). **^{13}C{^{1}H} NMR** (101 MHz, C_6D_6): δ 160.7 (dd, J =9.6 Hz, $^1J_{C,F}$ = 255.2 Hz), 141.6, 141.2 (dm), 134.8, 131.6, 127.2, 118.8 (dm), 105.5 (m). **^{19}F NMR** (376 MHz, C_6D_6): δ −95.1 (d, $^4J_{H,F}$ = 7.5 Hz, 4F). X-ray data for **B^9** (Fig S5): M = 698.53, colorless, monoclinic, *C2/c* (#15), a = 30.3351(3) Å, b = 8.1009(1) Å, c = 17.1872(2) Å, α = 90°, β = 101.162(1)°, γ = 90°, V = 4143.72(8) Å^3, Z = 8, *Dcalcd* = 2.239 g/cm^3, T = −150 °C, R_1 (wR_2) = 0.0309 (0.0782).

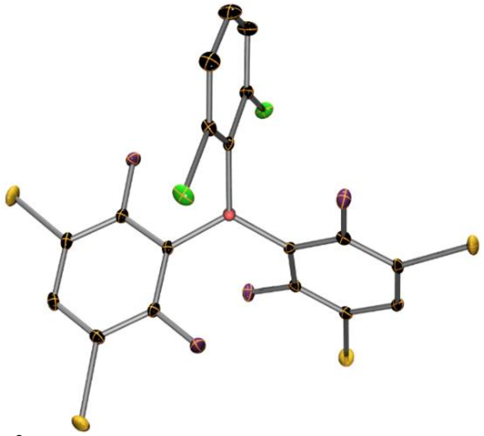

Fig S5. Molecular structure of B^9 with ellipsoids set at 30% probability. H atoms are omitted for clarity.

[6] Synthesis of B^{10}

6-1. Synthesis of 1,5-difluoro-2,4-di(3,5-bis(trifluoromethyl)phenyl)benzene

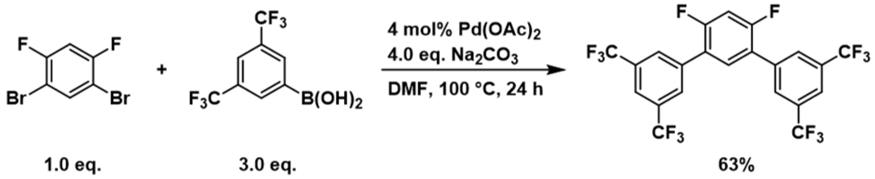

Fig S6. Synthesis of 1,5-difluoro-2,4-di(3,5-bis(trifluoromethyl)phenyl)benzene.

To a solution of 1,5-dibromo-2,4-difluorobenzene (1.51 g, 5.55 mmol, 0.11 M in N,N-dimethylformamide) was added 3,5-bis(trifluoromethyl)phenylboronic acid (4.31 g, 16.7 mmol), $Pd(OAc)_2$ (50.0 mg, 0.223 mmol) and Na_2CO_3 (2.36 g, 22.3 mmol). The reaction mixture was stirred at 100 °C for 24 h. Then, deionized water (30 mL) was added to the resultant mixture at room temperature, followed by the extraction of the organic layer with Et_2O (20 mL × 3). The combined organic layer was concentrated *in vacuo*. The residue was distilled at 170 °C under reduced pressure (ca. 0.2 mmHg) to afford 1,5-difluoro-2,4-di(3,5-bis(trifluoromethyl)phenyl)benzene as colorless liquid, which eventually solidified into a white solid at room temperature (1.87 g, 3.47 mmol, 63%). **^{1}H NMR** (400 MHz, $CDCl_3$): δ 7.99 (s, 4H), 7.94 (s, 2H), 7.54 (t, J = 8.2 Hz, 1H), 7.18 (t, J = 10.0 Hz, 1H). **^{13}C{^{1}H} NMR** (101 MHz, $CDCl_3$): δ 159.9 (dd, J = 12.1 Hz, $^1J_{C,F}$ = 256.5 Hz), 136.4, 132.4 (q, J = 33.7 Hz), 132.1, 129.3, 123.9 (dd, J = 6.6 Hz, J = 12.1 Hz), 123.3 (q, $^1J_{C,F}$ = 273.7 Hz), 122.3, 106.1 (t, J = 26.8 Hz). **^{19}F NMR** (376 MHz, $CDCl_3$): δ −62.9 (s, 12F), −112.0 (t, J = 7.5 Hz, 2F). HRMS (EI$^+$): m/z Calcd for $C_{22}H_8F_{14}$ 538.0402, found 538.0398.

6-2. Synthesis of 1-iode-2,6-difluoro-3,5-di(3,5-bis(trifluoromethyl)phenyl)benzene

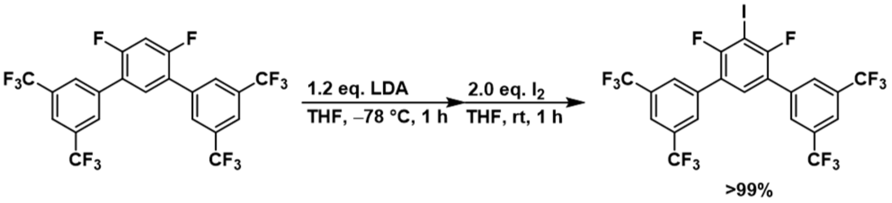

Fig S7. Synthesis of 1-iode-2,6-difluoro-3,5-di(3,5-bis(trifluoromethyl)phenyl)benzene.

A solution of diisopropylamine (1.19 mL, 8.47 mmol, 0.17 M in THF) was slowly treated with *n*-BuLi (5.3 mL, 8.5 mmol, 1.6 M in hexane) at −78 °C. After stirring at −78 °C for 1 h, the resultant solution was slowly transferred into a solution of 1,5-difluoro-2,4-di(3,5-bis(trifluoromethyl)phenyl)benzene (3.81 g, 7.08 mmol, 0.14 M in THF) at −78 °C. After stirring for 1 h at −78 °C, a solution of I_2 (3.6 g, 14 mmol, 0.28 M in THF) was added, and the resultant mixture was then allowed to warm to room temperature, where it was stirred for another 1 h. After diluted with 30 mL saturated aqueous $Na_2S_2O_3$ solution, the mixture was poured into water (100 mL) and the organic layer was extracted with hexane (50 mL × 3). The combined organic layer was concentrated *in vacuo* and purified by the chromatography on a silica gel eluted with hexane. Removal of all volatiles *in vacuo* afforded 1-iode-2,6-difluoro-3,5-di(3,5-bis(trifluoromethyl)phenyl)benzene as a white solid (4.70 g, 7.08 mmol, >99%). ^{1}**H NMR** (400 MHz, $CDCl_3$): δ 7.97 (s, 6H); 7.52 (t, J = 8.0 Hz, 1H). ^{13}**C{^{1}H} NMR** (101 MHz, $CDCl_3$): δ 159.6 (dd, J = 6.1 Hz, $^{1}J_{C,F}$ = 252.5 Hz), 135.9, 132.5 (q, J = 34.0 Hz), 132.0, 129.3, 123.9 (dd, J = 6.6 Hz, J = 15.2 Hz), 123.2 (q, $^{1}J_{C,F}$ = 273.7 Hz), 122.6, 74.1 (t, J = 31.3 Hz). ^{19}**F NMR** (376 MHz, $CDCl_3$): δ −62.8 (s, 12F), −92.3 (d, $^{4}J_{H,F}$ = 7.5 Hz, 2F). HRMS (EI^{+}): m/z Calcd for $C_{22}H_7F_{14}I$ 663.9369, found 663.9379.

6-3. Synthesis of B^{10}

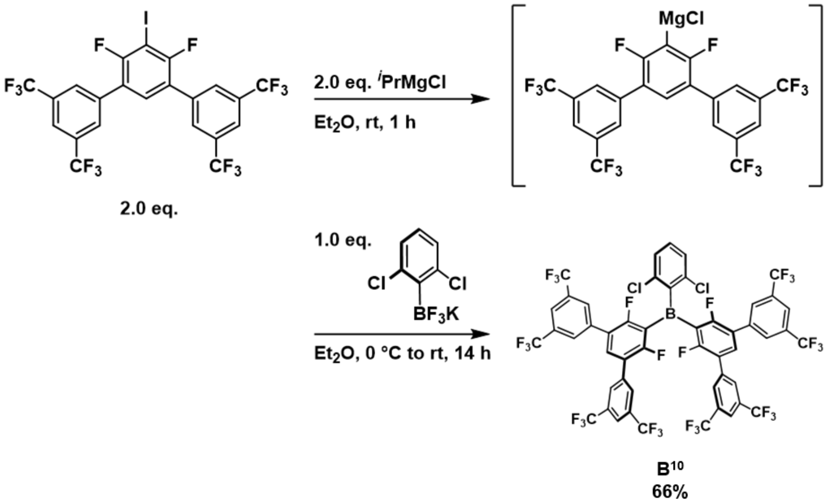

Fig S8. Synthesis of B^{10}.

A solution of 1-iode-2,6-difluoro-3,5-di(3,5-bis(trifluoromethyl)phenyl)benzene (2.15 g, 3.24 mmol, 0.11 M in Et_2O) was slowly treated with ^{i}PrMgCl (3.3 mL, 3.3 mmol, 1.0 M in Et_2O). After stirring at room temperature for 1 h, the resultant solution was transferred into a suspension of potassium (2,6-dichlorophenyl)trifluoroborate (410 mg, 1.62 mmol, 0.16 M in Et_2O) at 0 °C. The reaction mixture was then allowed to warm to room temperature, where it was stirred for another 14 h. After the removal of all volatiles *in vacuo*, the residue was extracted with α,α,α-trifluorotoluene (50 mL × 3; warmed to 70 °C prior to use). The combined organic layers were concentrated *in vacuo* and washed with hexane (cooled to −20 °C

prior to use) to afford **B^10^** as a white solid (1.32 g, 1.07 mmol, 66%). A single crystal suitable for X-ray diffraction analysis was prepared by recrystallization from α,α,α-trifluorotoluene at −35 °C. **^{1}H NMR** (400 MHz, $CDCl_3$): δ 7.94 (br, 12H), 7.71 (t, J = 8.2 Hz, 2H), 7.33 (br, 3H). **^{11}B NMR** (128 MHz, $CDCl_3$): Not observed. **^{13}C{^{1}H} NMR** (101 MHz, C_6D_6): δ 162.0 (d, $^{1}J_{C,F}$ = 173.7 Hz), 137.2, 136.2, 134.8, 132.4 (q, J = 22.6 Hz), 131.7, 129.4, 127.4, 123.9, 123.2 (q, $^{1}J_{C,F}$ = 182.8 Hz), 122.4. Resonances of the C_{ipso} with respect to the boron atom were not observed. **^{19}F NMR** (376 MHz, $CDCl_3$) δ −66.1 (s, 24F), −103.7 (d, $^{4}J_{H,F}$ = 7.5 Hz, 4F). X-ray data for **B^{10}** (Fig S9): M = 1234.51, colorless, monoclinic, *P2/n* (#13), a = 12.3665(3) Å, b = 9.6717(2) Å, c = 23.4111(5) Å, α = 90°, β = 100.471(2)°, γ = 90°, V = 2753.46(11) Å^3, Z = 2, *Dcalcd* = 1.489 g/cm^3, T = −150 °C, R_1 (wR_2) = 0.0831 (0.2620). In order to improve accuracy of the refinement, solvated C_7H_8 molecules were masked by the program Olex2 v1.3.

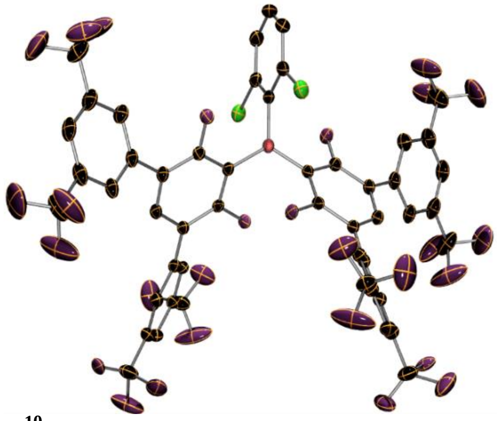

Fig S9. Molecular structure of B^{10} with ellipsoids set at 30% probability. H atoms are omitted for clarity.

[7] Synthesis of [Qin–H][HO–B^1]

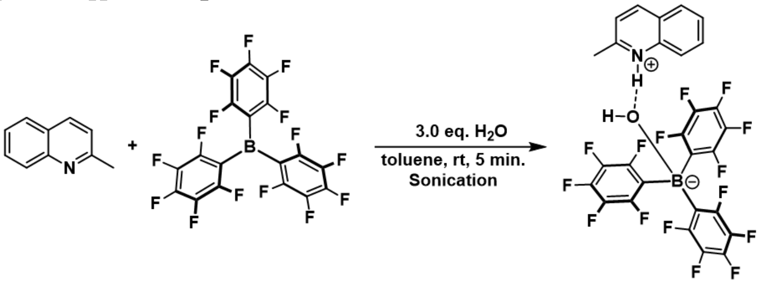

Qin
3.0 eq.

B^1
1.0 eq.

[Qin–H][HO–B^1]
74%

Fig S10. Synthesis of [Qin–H][HO–B^1].

To a solution of **B^1** (102 mg, 0.199 mmol, 0.040 M in toluene) was subsequently added **Qin** (86 mg, 0.60 mmol) and deionized H_2O (10.8 μL, 0.600 mmol). The reaction mixture was then sonicated (frequency: 37 kHz) for 5 min. After the removal of all volatiles *in vacuo*, the residue was washed with hexane to afford [**Qin**–H][HO–**B^1**] as a white solid (99.4 mg, 0.148 mmol, 74%). A single crystal suitable for X-ray diffraction analysis was prepared by recrystallization from toluene at room temperature. **^{1}H NMR** (400 MHz, CD_2Cl_2): δ 8.66 (d, J = 8.8 Hz, 1H), 8.05 (m, 2H), 7.89 (dt, J = 1.2 Hz, J = 7.9 Hz, 1H), 7.80 (t, J = 7.6 Hz, 1H), 7.60 (d, J = 8.8 Hz, 1H), 2.80 (s, 3H). Resonances of N–*H*/*H*O–B derived from H_2O were not confirmed. **^{11}B NMR** (128 MHz, CD_2Cl_2): δ −3.7 (s). **^{13}C{^{1}H} NMR** (101 MHz, CD_2Cl_2): δ 157.7, 148.3 (dm, $^{1}J_{C,F}$ = 241.4 Hz), 145.6, 139.3 (dd, J = 13.1 Hz, $^{1}J_{C,F}$ = 247.4 Hz), 138.7, 137.2 (dd, J = 14.1 Hz, $^{1}J_{C,F}$ = 228.3 Hz), 134.7, 129.9, 128.9, 127.2, 124.0, 123.3, 120.7, 20.6 (d, J = 7.1 Hz). **^{19}F NMR** (376 MHz, CD_2Cl_2): δ −135.8 (d, J = 18.8 Hz, 6F), −161.2 (t, J = 20.7 Hz, 3F), −165.7 (m, 6F). X-ray data for [**Qin**–H][HO–**B^1**] (Fig S11): M = 673.19, colorless, triclinic, *P-1* (#2), a = 8.2453(2) Å, b = 10.0093(2) Å,

c = 16.2936(3) Å, α = 98.863(2)°, β = 98.367(2)°, γ = 96.052(2)°, V = 1303.22(5) $Å^3$, Z = 2, *Dcalcd* = 1.715 g/cm^3, T = −100 °C, R_1 (wR_2) = 0.0322 (0.0928).

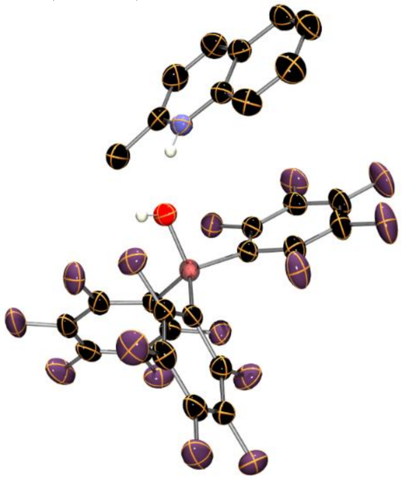

Fig S11. Molecular structure of [Qin–H][HO–B[1]] with ellipsoids set at 30% probability. H atoms except those derived from H_2O are omitted for clarity.

[8] Synthesis of [Qin–H][HO–B[9]]

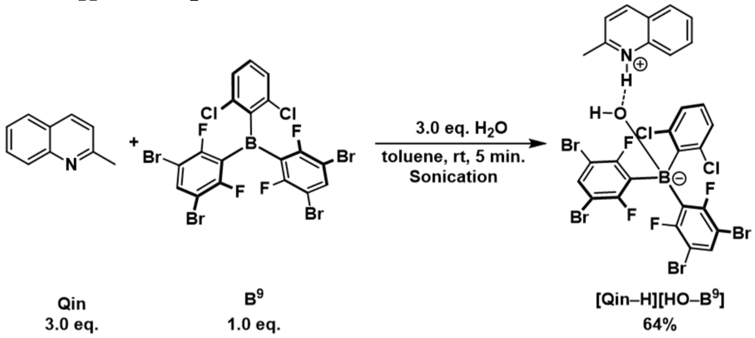

Fig S12. Synthesis of [Qin–H][HO–B[9]].

To a solution of **B[9]** (70 mg, 0.10 mmol, 0.020 M in toluene) was subsequently added **Qin** (43 mg, 0.30 mmol) and deionized H_2O (5.4 μL, 0.30 mmol). The reaction mixture was then sonicated (frequency: 37 kHz) for 5 min. After the removal of all volatiles *in vacuo*, the residue was washed with hexane to afford [**Qin**–H][HO–**B[9]**] as a white solid (54.7 mg, 0.0636 mmol, 64%). A single crystal suitable for X-ray diffraction analysis was prepared by recrystallization from toluene at room temperature. ^{1}**H NMR** (400 MHz, CD_2Cl_2): δ 8.53 (d, J = 8.4 Hz, 1H), 7.98 (t, J = 7.4 Hz, 2H), 7.78 (dt, J = 7.2 Hz, J = 11.3 Hz, 1H), 7.70 (d, J = 7.6 Hz, 1H), 7.51 (d, J = 8.4 Hz, 1H), 7.38 (m, 2H), 7.18 (d, J = 7.6 Hz, 2H), 7.01 (t, J = 7.6 Hz, 1H), 2.74 (s, 3H). Resonances of N–*H*/*H*O–B derived from H_2O were not confirmed. ^{11}**B NMR** (128 MHz, CD_2Cl_2): δ −1.6 (s). ^{13}**C{^{1}H} NMR** (101 MHz, CD_2Cl_2): δ 161.1 (d, J =17.2 Hz, $^{1}J_{C,F}$ = 243.4 Hz), 157.9, 144.0, 140.3, 139.8, 133.8, 132.7, 129.1, 128.4, 127.3, 127.1, 123.1, 122.1, 104.0, 104.0 (d, J = 32.3 Hz), 21.2 (d, J = 6.1 Hz). Resonances of the C_{ipso} with respect to the boron atom were not observed. ^{19}**F NMR** (376 MHz, CD_2Cl_2): δ −99.1 (d, J = 3.8 Hz, 4F). X-ray data for [**Qin**–H][HO–**B[9]**] (Fig S13): M = 859.73, colorless, monoclinic, $P2_1/n$ (#14), a = 13.6615(2) Å, b = 13.2687(2) Å, c = 18.1974(3) Å, α = 90°, β = 95.418(2)°, γ = 90°, V = 3283.91(9) $Å^3$, Z = 4, *Dcalcd* = 1.739 g/cm^3, T = −100 °C, R_1 (wR_2) = 0.0401 (0.1094). In order to improve accuracy of the refinement, solvated H_2O molecules were masked by the program Olex2 v1.3.

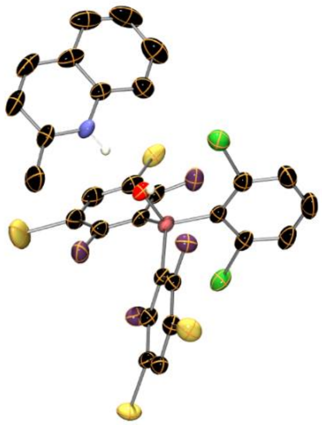

Fig S13. Molecular structure of [Qin–H][HO–B^9] with ellipsoids set at 30% probability. H atoms except those derived from H_2O are omitted for clarity.

[9] Evaluation of stability of B^7 and B^9 toward air and moisture

Crystalline powders of **B^7** and **B^9** were kept in a shelf under the ambient conditions (30% humidity, 22 °C), and the decomposition of these boranes were monitored by NMR analyses for the period of 1 year (Figs. S14–S15). These results revealed no observable decomposition on **B^9**, while a trace amount of decomposition was also confirmed for **B^7**.

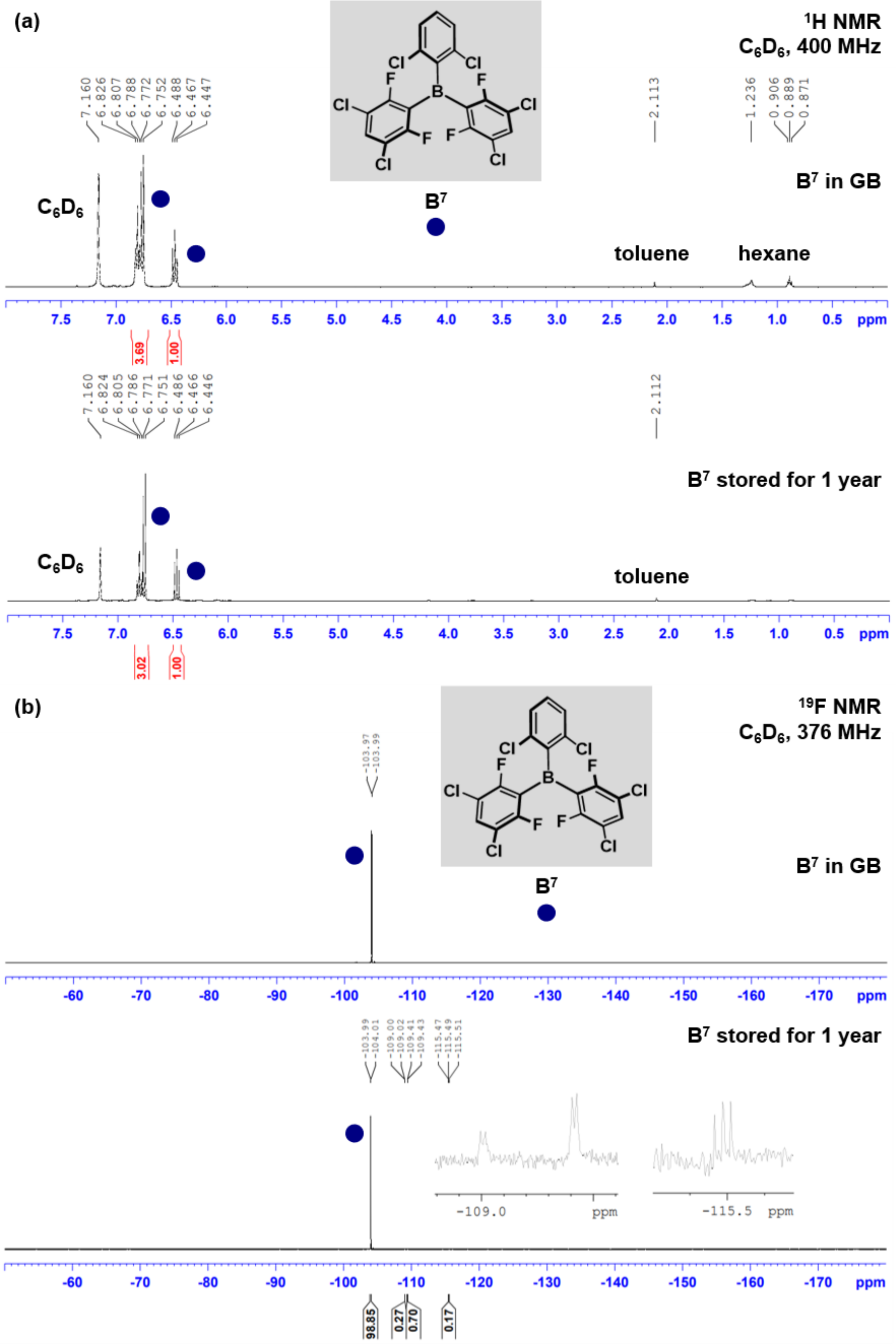

Fig S14. Stability of B^7 toward air and moisture. (a) ^{1}H NMR spectra. (b) ^{19}F NMR spectra.

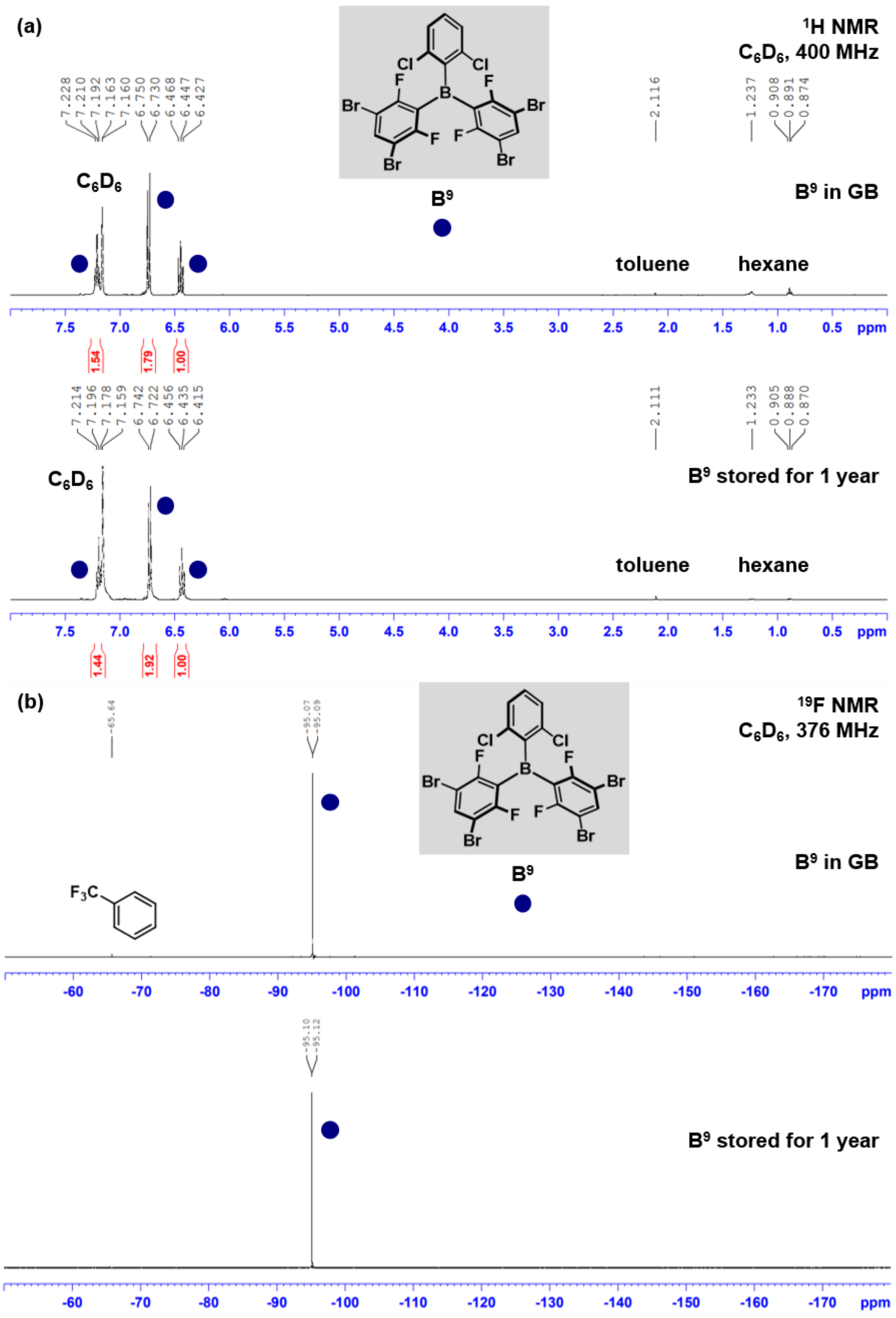

Fig S15. Stability of B[9] toward air and moisture. (a) ^{1}H NMR spectra. (b) ^{19}F NMR spectra.

[10] Titration of H_2O contaminated in the used gases

A pressure-tight NMR tube (Wilmad-LabGrass 542-PV-7; V = 1.8 mL) was charged with 1,3-dimethoxybenzene (65 mg, 0.47 mmol; an internal standard) and C_6D_6 (500 μL). Once sealed, the NMR tube was pressurized with H_2 (5 atm), CO_2 (5 atm), or $H_2/CO/CO_2$ (1.6 atm each) and analyzed by ^{1}H NMR. All experiments were repeated three times, and the averaged values (ppm) are used for the amount of H_2O contaminated in each gas reagent (Table S2).

Run	H_2	CO_2	$H_2/CO/CO_2$
1	34400	1420	3660
2	41500	1880	3380
3	34700	2090	3380
average	36867	1797	3473

Table S2. Amount of H_2O (ppm) contaminated in used gases.

[11] Screening of catalysts in hydrogenation of Qin

11-1. Reaction with transition-metal (TM) catalysts (runs 1–3 in Fig. 2)

General: A 10 mL autoclave was charged with **Qin**, TM complexes, tetradecane (an internal standard) and toluene. Once sealed, the autoclave was pressurized with $H_2/CO/CO_2$ (4 atm each; Condition A) or H_2 (4 atm; Condition B) and heated at 100 °C for 6 h. After degassed at room temperature, the yield of **H_4-Qin** was determined by GC analysis.

Run 1-A: Followed by the general procedures using $H_2/CO/CO_2$ (4 atm each), **Qin** (107.4 mg, 0.750 mmol), Rh-based complex **TM^1** (69.6 mg, 0.0752 mmol), tetradecane (55.8 mg), and toluene (0.5 mL) were employed, giving **H_4-Qin** in <1% GC yield.
Run 1-B: Followed by the general procedures using H_2 (4 atm), **Qin** (107.4 mg, 0.750 mmol), Rh-based complex **TM^1** (69.4 mg, 0.0750 mmol), tetradecane (57.7 mg), and toluene (0.5 mL) were employed, giving **H_4-Qin** in 14% GC yield.

Run 2-A: Followed by the general procedures using $H_2/CO/CO_2$ (4 atm each), **Qin** (107.4 mg, 0.750 mmol), Ru-based complex **TM^2** (8.10 mg, 0.00707 mmol), tetradecane (57.6 mg), and toluene (0.5 mL) were employed, giving **H_4-Qin** in 3% GC yield.
Run 2-B: Followed by the general procedures using H_2 (4 atm), **Qin** (107.4 mg, 0.750 mmol), Ru-based complex **TM^2** (8.12 mg, 0.00709 mmol), tetradecane (58.2 mg), and toluene (0.5 mL) were employed, giving **H_4-Qin** in 99% GC yield.

Run 3-A: Followed by the general procedures using $H_2/CO/CO_2$ (4 atm each), **Qin** (107.4 mg, 0.750 mmol), $[Ir(cod)_2Cl]_2$ (5.08 mg, 0.00694 mmol), *rac*-BINAP (10.4 mg, 0.0167 mmol), I_2 (19.2 mg, 0.0756 mmol), tetradecane (60.2 mg), and toluene (0.5 mL) were employed, giving **H_4-Qin** in 1% GC yield.
Run 3-B: Followed by the general procedures using H_2 (4 atm), **Qin** (107.4 mg, 0.750 mmol), $[Ir(cod)_2Cl]_2$ (5.04 mg, 0.00689 mmol), *rac*-BINAP (10.2 mg, 0.0164 mmol), I_2 (19.1 mg, 0.0753 mmol), tetradecane (60.7 mg), and toluene (0.5 mL) were employed, giving **H_4-Qin** in 87% GC yield.

Experiments using Ru-MACHO (purchased from TCI and used as received): Followed by the general procedures using $H_2/CO/CO_2$ (4 atm each), **Qin** (358.3 mg, 2.50 mmol), Ru-MACHO (15.1 mg, 0.249 mmol), tetradecane (61.7 mg), and toluene (1.7 mL) were employed; however, production of **H_4-Qin** was detected in <1% GC yield.

We have also carried out the following experiment based on the literature conditions that were applied for the hydrogenation of Ru complexes supported by pincer ligands;[51] we employed $H_2/CO/CO_2$ (4 atm each), **Qin** (358.7 mg, 2.50 mmol), Ru-MACHO (15.2 mg, 0.0250 mmol), KO^{t}Bu (28.1 mg, 0.250 mmol), tetradecane (59.7 mg), and THF (1.7 mL), and heated at 100 °C for 6 h. However, **H_4-Qin** was detected in <1% GC yield.

Experiment using Pd/C: Followed by the general procedures using $H_2/CO/CO_2$ (4 atm each), **Qin** (356.5 mg, 2.49 mmol), Pd/C (Pd 10% on carbon, 26.7 mg, 0.0251 mmol), tetradecane (60.5 mg), and toluene (1.7 mL) were employed, giving **H_4-Qin** in 1% GC yield.

11-2. Reaction with B^n catalyst (runs 4–13 in Fig. 2 and Fig. S16)

General using $H_2/CO/CO_2$ (Condition A): A 30 mL autoclave was charged with **Qin** (2.5 mmol), **B^n** (0.025 mmol; 1 mol%), and tetradecane (an internal standard), and toluene (1.67 mL). Once sealed, the autoclave was pressurized with $H_2/CO/CO_2$ (4 atm each) and heated at 100 °C for 6 h. After degassed at room temperature, the yield of **H_4-Qin** was determined by GC analysis (Fig. 2).

General using H_2 (Condition B): A autoclave was charged with **Qin** (0.75 mmol, otherwise noted), **B^n** (0.075 mmol; 10 mol% otherwise noted), and toluene (0.5 mL). Tetradecane or pentadecane was added as an internal standard. Once sealed, the autoclave was pressurized with H_2 (4 atm) and heated at 100 °C for 6 h. After degassed at room temperature, the yield of **H_4-Qin** was determined by GC analysis (for run 4-B using 1 mol% **B^1**, see Fig. 2; for others, see Fig. S16).

Run 4-A: Followed by the general procedures using $H_2/CO/CO_2$ (4 atm each), **Qin** (359.5 mg), **B^1** (12.8 mg), and tetradecane (57.2 mg) were employed, giving **H_4-Qin** in 12% GC yield.
Run 4-B: Followed by the general procedures using H_2 (4 atm), **Qin** (356.9 mg), **B^1** (12.9 mg; 1 mol%), and tetradecane (57.2 mg) were employed, giving **H_4-Qin** in >99% GC yield.

Run 5-A: Followed by the general procedures using $H_2/CO/CO_2$ (4 atm each), we independently conducted two experiments using **B^2** to accurately evaluate the catalyst activity of **B^2**. In the first experiment, **Qin** (356.1 mg), **B^2** (11.5 mg), and tetradecane (68.7 mg) were employed to afford **H_4-Qin** in 62% GC yield. In the second, **Qin** (357.9 mg), **B^2** (11.4 mg), and tetradecane (65.5 mg) were employed to afford **H_4-Qin** in 63% GC yield. The average of these two experiments (63%) is shown in Fig. 2.
Run 5-B: Followed by the general procedures with a 10 mL autoclave using H_2 (4 atm), **Qin** (107.4 mg), **B^2** (34.4 mg), and tetradecane (71.2 mg) were employed, giving **H_4-Qin** in 99% GC yield.

Run 6-A: Followed by the general procedures using $H_2/CO/CO_2$ (4 atm each), we independently conducted two experiments using **B^3** to accurately evaluate the catalyst activity of **B^3**. In the first experiment, **Qin** (357.4 mg), **B^3** (15.0 mg), and tetradecane (60.7 mg) were employed to afford **H_4-Qin** in 27% GC yield. In the second, **Qin** (362.0 mg), **B^3** (14.9 mg), and tetradecane (63.4 mg) were employed to afford **H_4-Qin** in 36% GC yield. The average of these two experiments (32%) is shown in Fig. 2.
Run 6-B: Followed by the general procedures with a 10 mL autoclave using H_2 (4 atm), **Qin** (107.4 mg), **B^3** (44.5 mg), and tetradecane (71.2 mg) were employed, giving **H_4-Qin** in >99% GC yield.

Run 7-A: Followed by the general procedures using $H_2/CO/CO_2$ (4 atm each), **Qin** (360.8 mg), **B^4** (10.7 mg), and tetradecane (59.9 mg) were employed, giving **H_4-Qin** in 2% GC yield.

Run 7-B: Followed by the general procedures with a 10 mL autoclave using H_2 (4 atm), **Qin** (107.4 mg), **B**^{4} (32.0 mg), and pentadecane (75.3 mg) were employed, giving **H$_4$-Qin** in 84% GC yield.

Run 8-A: Followed by the general procedures using $H_2/CO/CO_2$ (4 atm each), we independently conducted three experiments using **B**^{5} to accurately evaluate the catalyst activity of **B**^{5}. In the first experiment, **Qin** (360.8 mg), **B**^{5} (11.5 mg), and tetradecane (59.6 mg) were employed to afford **H$_4$-Qin** in 72% GC yield. In the second, **Qin** (358.9 mg), **B**^{5} (11.4 mg), and tetradecane (63.2 mg) were employed to afford **H$_4$-Qin** in 86% GC yield. In the third, **Qin** (358.3 mg), **B**^{5} (11.4 mg), and tetradecane (61.3 mg) were employed to afford **H$_4$-Qin** in 85% GC yield. The average of these three experiments (81%) is shown in Fig. 2.
Run 8-B: Followed by the general procedures with a 10 mL autoclave using H_2 (4 atm), **Qin** (107.4 mg), **B**^{5} (34.1 mg), and pentadecane (71.3 mg) were employed, giving **H$_4$-Qin** in >99% GC yield.

Run 9-A: Followed by the general procedures using $H_2/CO/CO_2$ (4 atm each), **Qin** (354.6 mg), **B**^{6} (9.6 mg), and tetradecane (64.2 mg) were employed, giving **H$_4$-Qin** in 3% GC yield.
Run 9-B: Followed by the general procedures with a 10 mL autoclave using H_2 (4 atm), **Qin** (107.4 mg), **B**^{6} (28.7 mg), and tetradecane (75.4 mg) were employed, giving **H$_4$-Qin** in 71% GC yield.

Run 10-A: Followed by the general procedures using $H_2/CO/CO_2$ (4 atm each), we independently conducted three experiments using **B**^{7} to accurately evaluate the catalyst activity of **B**^{7}. In the first experiment, **Qin** (355.8 mg), **B**^{7} (13.1 mg), and tetradecane (60.2 mg) were employed to afford **H$_4$-Qin** in 67% GC yield. In the second, **Qin** (358.8 mg), **B**^{7} (13.1 mg), and tetradecane (58.8 mg) were employed to afford **H$_4$-Qin** in 87% GC yield. In the third, **Qin** (355.5 mg), **B**^{7} (13.1 mg), and tetradecane (63.6 mg) were employed to afford **H$_4$-Qin** in 87% GC yield. The average of these three experiments (80%) is shown in Fig. 2.
Run 10-B: Followed by the general procedures with a 10 mL autoclave using H_2 (4 atm), **Qin** (122.6 mg), **B**^{7} (39.0 mg), and pentadecane (66.1 mg) were employed, giving **H$_4$-Qin** in >99% GC yield.

Run 11-A: Followed by the general procedures using $H_2/CO/CO_2$ (4 atm each), **Qin** (356.1 mg), **B**^{8} (11.3 mg), and tetradecane (60.5 mg) were employed, giving **H$_4$-Qin** in 40% GC yield.
Run 11-B: Followed by the general procedures with a 10 mL autoclave using H_2 (4 atm), **Qin** (107.4 mg), **B**^{8} (33.9 mg), and tetradecane (59.1 mg) were employed, giving **H$_4$-Qin** in >99% GC yield.

Run 12-A: Followed by the general procedures using $H_2/CO/CO_2$ (4 atm each), we independently conducted three experiments using **B**^{9} to accurately evaluate the catalyst activity of **B**^{9}. In the first experiment, **Qin** (360.1 mg), **B**^{9} (17.5 mg), and tetradecane (60.5 mg) were employed to afford **H$_4$-Qin** in 83% GC yield. In the second, **Qin** (356.0 mg), **B**^{9} (17.4 mg), and tetradecane (60.7 mg) were employed to afford **H$_4$-Qin** in 86% GC yield. In the third, **Qin** (358.8 mg), **B**^{9} (17.6 mg), and tetradecane (60.8 mg) were employed to afford **H$_4$-Qin** in 82% GC yield. The average of these three experiments (84%) is shown in Fig. 2.

Run 12-B: Followed by the general procedures with a 30 mL autoclave using H_2 (4 atm), **Qin** (108.6 mg), **B**^{9} (52.4 mg), and tetradecane (58.6 mg) were employed, giving **H_4-Qin** in >99% GC yield.

Run 13-A: Followed by the general procedures using H_2/CO/CO_2 (4 atm each), we independently conducted three experiments using **B**^{10} to accurately evaluate the catalyst activity of **B**^{10}. In the first experiment, **Qin** (359.1 mg), **B**^{10} (30.7 mg), and tetradecane (60.1 mg) were employed to afford **H_4-Qin** in 63% GC yield. In the second, **Qin** (360.6 mg), **B**^{10} (30.7 mg), and tetradecane (62.8 mg) were employed to afford **H_4-Qin** in 86% GC yield. In the third, **Qin** (359.8 mg), **B**^{10} (30.9 mg), and tetradecane (60.3 mg) were employed to afford **H_4-Qin** in 77% GC yield. The average of these three experiments (75%) is shown in Fig. 2.
Run 13-B: Followed by the general procedures with a 30 mL autoclave using H_2 (4 atm), **Qin** (114.0 mg), **B**^{10} (92.2 mg), and tetradecane (62.0 mg) were employed, giving **H_4-Qin** in >99% GC yield.

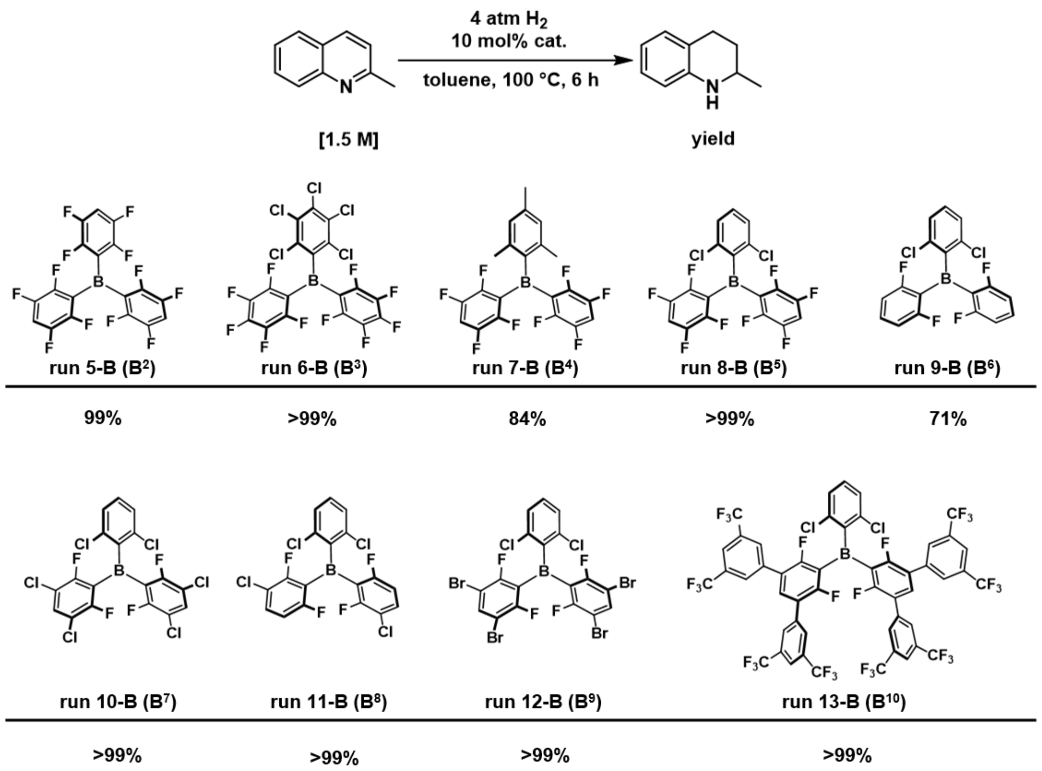

Fig S16. Hydrogenation of Qin using H_2.

[12] Hydrogenation of Qin using H_2/CO/CO_2 without solvent

12-1. Screening of catalysts (Fig S17)

General: A 30 mL autoclave was charged with **Qin** (3.0 mmol), **B**^{n} (0.030 mmol), and tetradecane (an internal standard). Once sealed, the autoclave was pressurized with H_2/CO/CO_2 (4 atm each; Condition A) or H_2 (4 atm; Condition B) and heated at 100 °C for 6 h. After degassed at room temperature, the yield of **H_4-Qin** was determined by GC analysis.

Run 1-A: Followed by the general procedures using $H_2/CO/CO_2$ (4 atm each), **Qin** (429.5 mg), **B[1]** (15.5 mg), and tetradecane (233.9 mg) were employed, giving **H_4-Qin** in 3% GC yield.
Run 1-B: Followed by the general procedures using H_2 (4 atm), **Qin** (431.6 mg), **B[1]** (15.3 mg), and tetradecane (235.6 mg) were employed, giving **H_4-Qin** in 7% GC yield.

Run 2-A: Followed by the general procedures, **Qin** (431.3 mg), **B[5]** (13.7 mg), and tetradecane (231.2 mg) were employed, giving **H_4-Qin** in 97% GC yield.

Run 3-A: Followed by the general procedures, **Qin** (438.1 mg), **B[7]** (15.6 mg), and tetradecane (237.8 mg) were employed, giving **H_4-Qin** in 99% GC yield.

Run 4-A: Followed by the general procedures, **Qin** (439.9 mg), **B[9]** (21.0 mg), and tetradecane (236.4 mg) were employed, giving **H_4-Qin** in >99% GC yield. We also carried out this reaction with **Qin** (423.8 mg), **B[9]** (20.9 mg), and tetradecane (242.8 mg) for 3 h, giving **H_4-Qin** in 81% GC yield

Run 5-A: Followed by the general procedures, **Qin** (436.1 mg), **B[10]** (36.8 mg), and tetradecane (227.6 mg) were employed, giving **H_4-Qin** in 84% GC yield.

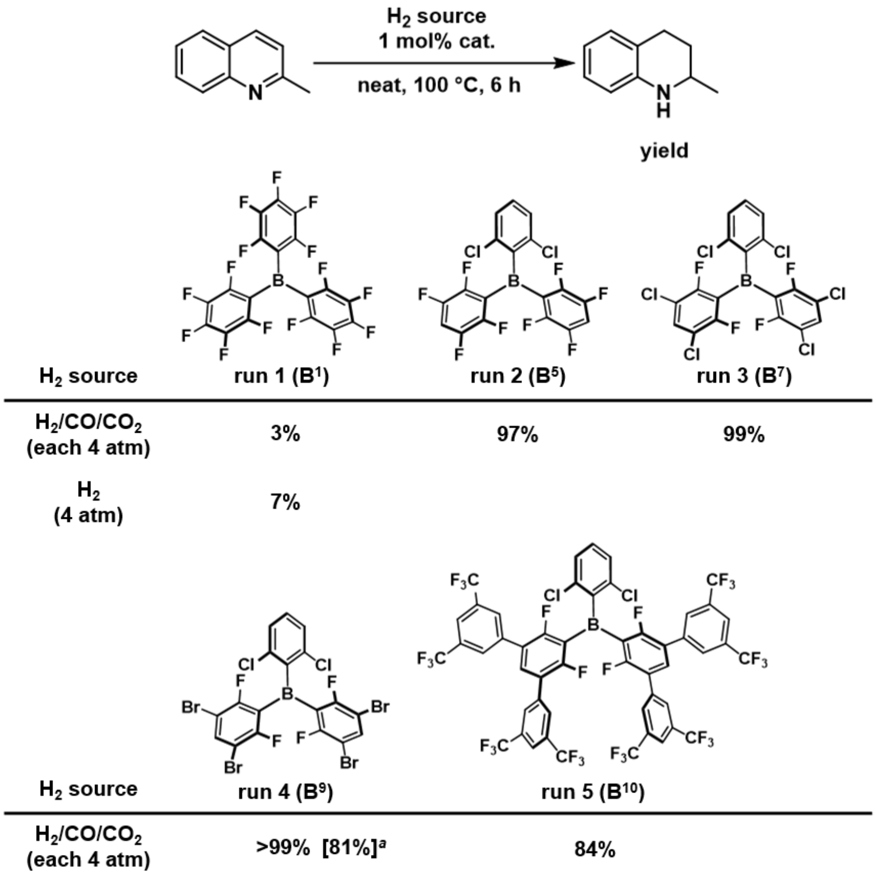

Fig S17. Screening of catalysts. [a]3 h.

12-2. Catalyst turnover number exhibited by B^n (n = 1, 5, 7, 9, 10) under solvent-free and mixed gas conditions (Fig S18)

General: A 30 mL autoclave was charged with **Qin** (25.0 mmol), $\mathbf{B^n}$ (0.025 mmol), and tetradecane (an internal standard). Once sealed, the autoclave was pressurized with $H_2/CO/CO_2$ (30 atm each; Condition A) or H_2 (85 atm; Condition B) and heated at 100 °C for 48 h. After degassed at room temperature, the yield of **H_4-Qin** was determined by GC analysis.

Run 1-A: Followed by the general procedures using $H_2/CO/CO_2$ (30 atm each), **Qin** (3.578 g), $\mathbf{B^1}$ (12.9 mg), and tetradecane (240.0 mg) were employed, giving **H_4-Qin** in <1% GC yield.

Run 2-A: Followed by the general procedures using $H_2/CO/CO_2$ (30 atm each), **Qin** (3.580 g), $\mathbf{B^5}$ (11.3 mg), and tetradecane (233.5 mg) were employed, giving **H_4-Qin** in 50% GC yield.

Run 3-A: Followed by the general procedures using $H_2/CO/CO_2$ (30 atm each), **Qin** (3.575 g), $\mathbf{B^7}$ (13.1 mg), and tetradecane (242.2 mg) were employed, giving **H_4-Qin** in 70% GC yield.

Run 4-A: Followed by the general procedures using $H_2/CO/CO_2$ (30 atm each), **Qin** (3.593 g), $\mathbf{B^9}$ (17.5 mg), and tetradecane (239.5 mg) were employed, giving **H_4-Qin** in 76% GC yield.
Run 4-B: Followed by the general procedures using H_2 (85 atm), **Qin** (7.16 g, 50.0 mmol), $\mathbf{B^9}$ (17.6 mg), and tetradecane (240.0 mg) were employed, giving **H_4-Qin** in 74% GC yield.

Run 5-A: Followed by the general procedures using $H_2/CO/CO_2$ (30 atm each), **Qin** (3.582 g), $\mathbf{B^{10}}$ (30.8 mg), and tetradecane (244.2 mg) were employed, giving **H_4-Qin** in 67% GC yield.

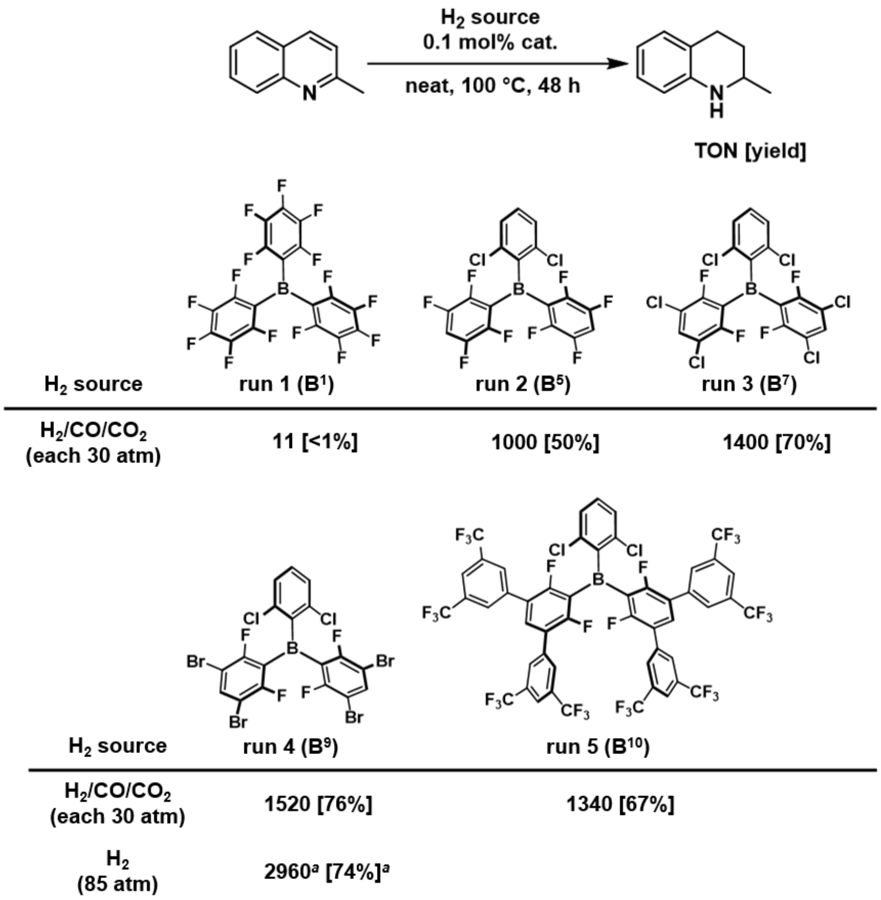

Fig S18. Catalyst turnover number exhibited by B^{n} (n = 1, 5, 7, 9, 10) under the solvent-free and mixed gas conditions. [a]0.05 mol% B^{9} was used.

12-3. Exploration of the gas compositions included in H_2-source

General: A 30 mL autoclave was charged with **Qin** (3.0 mmol), **B^{9}** (0.030 mmol), and tetradecane (an internal standard). Once sealed, the autoclave was pressurized with each H_2-source and heated at 100 °C. After degassed at room temperature, the yield of **H_4-Qin** was determined by GC analysis. The results are shown in Fig 3a.

Use of H_2/CO/CO_2 (4 atm/20 atm/4 atm): Followed by the general procedures, **Qin** (426.8 mg), **B^{9}** (21.0 mg), and tetradecane (238.1 mg) were employed and the autoclave was pressurized with CO (16 atm) and H_2/CO/CO_2 (4 atm each). After period of 6 h, **H_4-Qin** was afforded in 81% GC yield. We also carried out this reaction with **Qin** (424.6 mg), **B^{9}** (21.0 mg), and tetradecane (241.6 mg) for 12 h, giving **H_4-Qin** in >99% GC yield

Use of H_2/CO/CO_2 (4 atm/4 atm/20 atm): Followed by the general procedures, **Qin** (425.2 mg), **B^{9}** (20.8 mg), and tetradecane (238.9 mg) were employed and the autoclave was pressurized with CO_2 (16 atm) and H_2/CO/CO_2 (4 atm each). After period of 6 h, **H_4-Qin** was afforded in 34% GC yield. We also carried out this reaction with **Qin** (425.8 mg), **B^{9}** (21.0 mg), and tetradecane (244.1 mg) for 40 h, giving **H_4-Qin** in 94% GC yield

Use of $H_2/CO/CO_2/CH_4$ (4 atm/4 atm/4 atm/4 atm): Followed by the general procedures, **Qin** (429.4 mg), **B^9** (20.9 mg), and tetradecane (239.7 mg) were employed and the autoclave was pressurized with CH_4 (4 atm) and $H_2/CO/CO_2$ (4 atm each). After period of 6 h, **H_4-Qin** was afforded in 97% GC yield.

[13] Screening of catalysts in dehydrogenation of H_4-Qin

General: A 10 mL two-necked flask was charged with **H_4-Qin** (3.8 mmol), **B^n** (0.075 mmol), and tetradecane (an internal standard). The reaction mixture was heated at 200 °C for 6 h, and then allowed to cool to room temperature. The yield of **Qin** was determined by GC analysis. The results are shown in Fig 3b.

Run 1: Followed by the general procedures, **H_4-Qin** (552.5 mg), **B^1** (38.1 mg), and tetradecane (102.6 mg) were employed, giving **Qin** in 18% GC yield.

Run 2: Followed by the general procedures, **H_4-Qin** (534.8 mg), **B^5** (34.1 mg), and tetradecane (100.1 mg) were employed, giving **Qin** in 84% GC yield.

Run 3: Followed by the general procedures, **H_4-Qin** (539.3 mg), **B^7** (39.1 mg), and tetradecane (96.6 mg) were employed, giving **Qin** in 85% GC yield.

Run 4: Followed by the general procedures, **H_4-Qin** (535.4 mg), **B^9** (52.4 mg), and tetradecane (100.0 mg) were employed, giving **Qin** in 88% GC yield.

Run 5: Followed by the general procedures, **H_4-Qin** (538.1 mg), **B^{10}** (92.4 mg), and tetradecane (100.9 mg) were employed, giving **Qin** in 68% GC yield.

[14] H_2 recovery via B^9-catalyzed dehydrogenation of H_4-Qin

The schematic representation of a reaction setup is shown in Fig S19. A 10 mL two-neck flask was charged with **H_4-Qin** (542 mg, 3.79 mmol), **B^9** (52.4 mg, 0.0750 mmol) and tetradecane (103.9 mg; an internal standard). The reaction mixture was stirred at 200 °C for 2 h, and then allowed to cool to room temperature. H_2 was collected in a graduated cylinder to calculate the yield of H_2 based on its collected volume (164 mL, 7.32 mmol). The yield of **Qin** and the purity of the collected H_2 were determined by GC analysis.

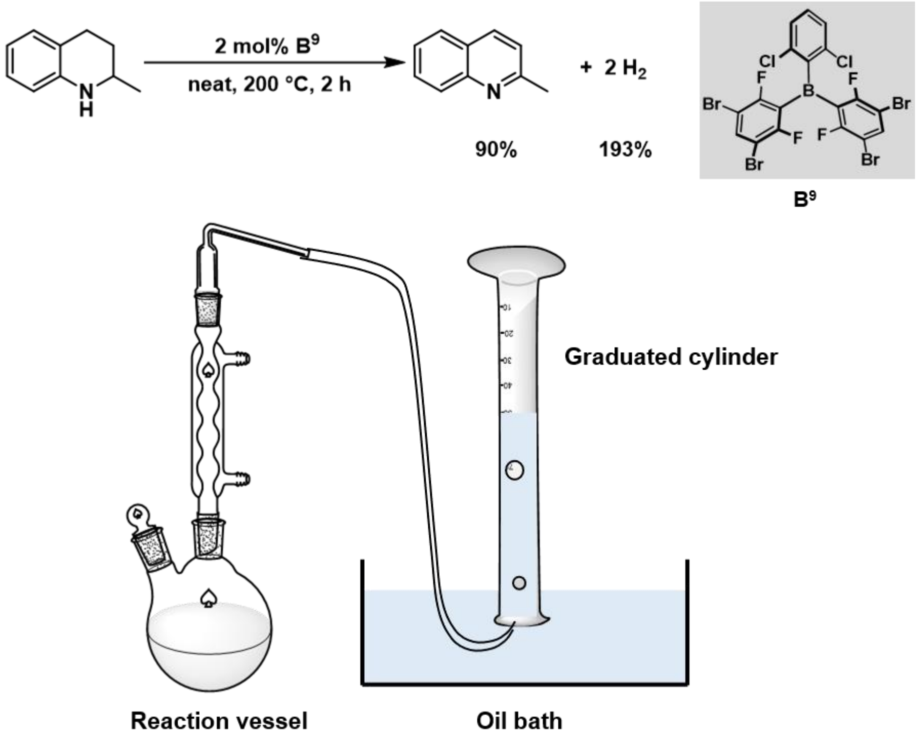

Fig S19. The reaction setup for H_2 collection.

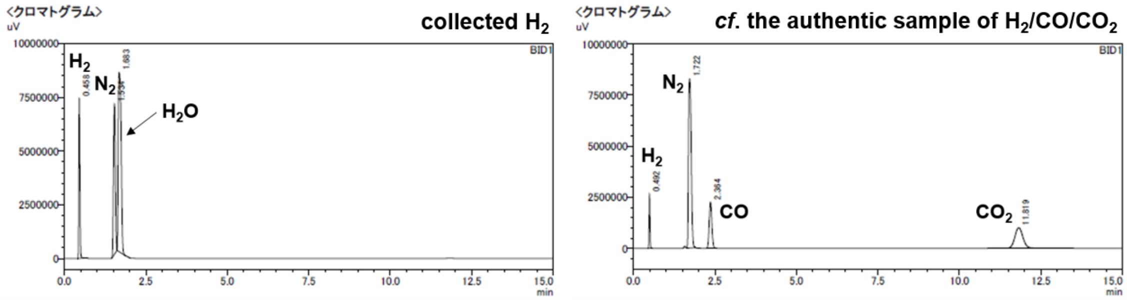

Fig S20. GC chromatograms for the collected H_2 gas (shown in left) and an authentic sample of $H_2/CO/CO_2$ (1:1:1 molar ratio; shown in right) used in this work.

[15] B^9-catalyzed hydrogenation/dehydrogenation sequences from $H_2/CO/CO_2$

A 30 mL autoclave was charged with **Qin** (145 mg, 1.01 mmol), $\mathbf{B^9}$ (13.9 mg, 0.199 mmol), and tetradecane (59.5 mg; an internal standard). Once sealed, the autoclave was pressurized with $H_2/CO/CO_2$ (4 atm each; 5.4 mmol each based on n = PV/RT, where T = 273.15 K, P = 4 atm, V = 30 mL) and heated to 100 °C for 2 h. After cooling to room temperature, all volatiles were removed *in vacuo*, and GC analysis showed the production of $\mathbf{H_4}$**-Qin** in 95%. Then, the reaction mixture was transferred into a 10 mL two-neck flask (Fig. S19). During this manipulation, the residue inside the autoclave reactor was extracted with toluene in order to minimize the loss of reagents. The toluene was then removed *in vacuo*; however, this is not essential for the following dehydrogenation. The reaction mixture was then stirred at 200 °C for 3 h. The volume of collected gas was measured using a graduated cylinder to calculate the yield of H_2 (39.0 mL, 1.74 mmol). The conversion of $\mathbf{H_4}$**-Qin** (94%), the yield of **Qin** (91%), and the purity of the collected H_2 gas were determined using GC analysis. As shown in Fig. S21, CO was not detected (or its concentration is less than a detection limit of the GC), while 0.1% molar of CO_2 with respect to that of H_2 was detected

after the background calibration (vide infra). However, we cannot rule out a possibility that a trace amount of CO_2 (0.1%) was contaminated during the collection of H_2 (i.e. from silicon oil) and/or the injection of the recovered H_2 into the GC (i.e. from air).

$$
\begin{aligned}
H_2 \text{ purity} &= (\text{molar amount of } H_2)/(\text{sum of the molar amounts of } H_2\text{, CO, and } CO_2)\times 100 \\
&= (1.74)/(1.74 + 0 + 0.00178)\times 100 \\
&= 99.9\%
\end{aligned}
$$

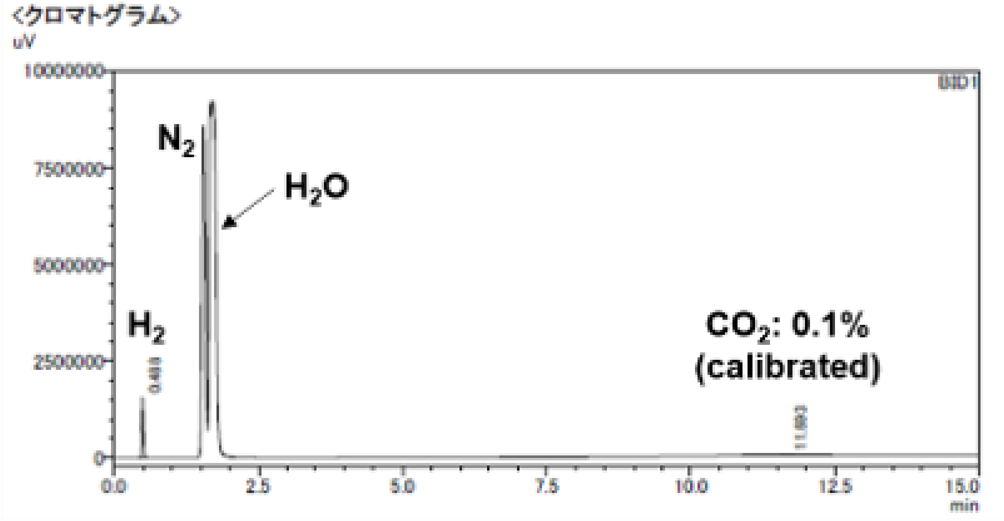

Fig S21. The GC chromatogram for the collected H_2.

We experimentally confirmed that a certain amount of CO_2 and H_2O were inevitably contaminated from air and/or silicon oil that was used for H_2 collection. In order to accurately evaluate the H_2 purity, we performed a background calibration of CO_2 through several control experiments using N_2 gas. In fact, as shown in Table S3, the contamination of CO_2 was always confirmed when N_2 gas, collected through the same experimental procedures as mentioned above (Fig. S19), was analyzed by GC. Based on these experiments, an average amount of CO_2 contaminated from air and/or silicon oil was estimated as shown in Table S3, which was used for the correction of H_2 purity.

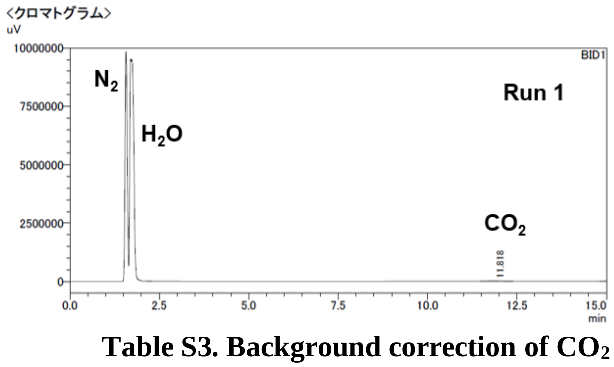

Run	Area values of CO_2
1	272898
2	273634
3	278042
average	274858
standard error	1600

Table S3. Background correction of CO_2 contaminated from air/silicon oil.

[16] Optimization of reaction conditions for hydrogenation of Lut

General: The gases used in this reaction was dehydrated over molecular sieves (4 Å) prior to use for at least 12 h. A 30 mL autoclave was charged with **Lut** (1.0 mmol), $\mathbf{B^{n}}$ (0.10 mmol), and tetradecane (an internal standard). Once sealed, the autoclave was pressurized with H_2-source and heated at 150 °C for 6 h. After degassed at room temperature, the yield of $\mathbf{H_6}$**-Lut** was determined by GC analysis. The results of runs 1–4 are shown in Fig 3d and those of run 5–6 are shown in Fig S22.

Run 1: Followed by the general procedures using dehydrated H_2 (20 atm), **Lut** (108.9 mg), $\mathbf{B^{7}}$ (52.0 mg), and tetradecane (79.4 mg) were employed and the autoclave was pressurized with dehydrated H_2 (20 atm), giving $\mathbf{H_6}$**-Lut** in 81% GC yield. We also carried out this reaction with **Lut** (102.7 mg), $\mathbf{B^{9}}$ (69.6 mg), and tetradecane (79.9 mg), giving $\mathbf{H_6}$**-Lut** in 41% GC yield

Run 2: Followed by the general procedures using dehydrated H_2/CO (20 atm/4 atm), **Lut** (108.8 mg), $\mathbf{B^{7}}$ (51.9 mg), and tetradecane (81.9 mg) were employed and the autoclave was pressurized with dehydrated H_2/CO (4 atm each) and H_2 (16 atm), giving $\mathbf{H_6}$**-Lut** in 54% GC yield.

Run 3: Followed by the general procedures using dehydrated H_2/CO (40 atm/4 atm), **Lut** (112.1 mg), $\mathbf{B^{7}}$ (52.1 mg), and tetradecane (81.8 mg) were employed and the autoclave was pressurized with dehydrated H_2/CO (4 atm each) and H_2 (36 atm), giving $\mathbf{H_6}$**-Lut** in 70% GC yield.

Run 4: Followed by the general procedures using dehydrated H_2/CO_2 (40 atm/4 atm), **Lut** (110.3 mg), $\mathbf{B^{7}}$ (52.0 mg), and tetradecane (83.2 mg) were employed and the autoclave was pressurized with dehydrated H_2/CO_2 (4 atm each) and H_2 (36 atm), giving $\mathbf{H_6}$**-Lut** in 53% GC yield.

Run 5: Followed by the general procedures using H_2 (20 atm), **Lut** (114.1 mg), $\mathbf{B^{7}}$ (52.1 mg), and tetradecane (87.4 mg) were employed and the autoclave was pressurized with H_2 (20 atm; not treated with molecular sieves (4 Å)), giving $\mathbf{H_6}$**-Lut** in 72% GC yield.

Run 6: Followed by the general procedures using dehydrated H_2/CO (4 atm each), **Lut** (109.0 mg), $\mathbf{B^{7}}$ (52.1 mg), and tetradecane (81.9 mg) were employed and the autoclave was pressurized with dehydrated H_2/CO (4 atm each), giving $\mathbf{H_6}$**-Lut** in 38% GC yield.

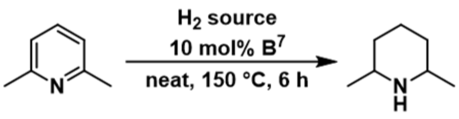

run	H_2 source (atm)	yield
5[a]	H_2 (20)	72%
6	H_2/CO (4/4)	38%

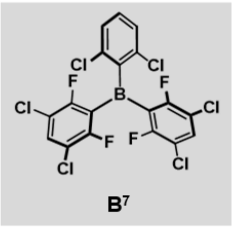

Fig S22. Hydrogenation of Lut. [a]H_2 without dehydration.

[17] Mechanistic studies

17-1. Determination of the reaction rate constants in the hydrogenation of Qin

General using $H_2/CO/CO_2$ (Condition A): A mixture of **Qin** (1.5 M), **B^n** ([**B^n** = 0.015 M]), and tetradecane (an internal standard) in toluene was prepared and equally divided into 10 autoclave reactors (*V* = 10 mL). Once sealed, each reactor was pressurized with $H_2/CO/CO_2$ (10 atm each) and heated at 100 °C. The conversion of **Qin** and the yield of **H_4-Qin** were monitored by GC analysis. In several cases, these experiments were repeated twice to confirm the reproducibility.

General using H_2 (Condition B): A mixture of **Qin** (1.5 M), **B^n** ([**B^n** = 0.015 M]), and tetradecane (an internal standard) in toluene was prepared and equally divided into 10 autoclave reactors (*V* = 10 mL). Once sealed, each reactor was pressurized with H_2 (10 atm) and heated at 100 °C. The conversion of **Qin** and the yield of **H_4-Qin** were monitored by GC analysis. In several cases, these experiments were repeated twice to confirm the reproducibility.

Run 1-A using B^9: Followed by the general procedures using $H_2/CO/CO_2$ (10 atm each), we independently conducted two experiments to confirm the reproducibility. In the first experiment, **Qin** (1.07 g, 7.47 mmol), **B^9** (52.4 mg, 0.0750 mmol), tetradecane (597 mg), and toluene (5.0 mL) were employed, which demonstrated that the rate constant for the formation of **H_4-Qin** is 1.93(10) × 10^{-4} (mol m^{-3} s^{-1}) as shown in Fig.S23. In the second, **Qin** (1.29 g, 9.01 mmol), **B^9** (63.1 mg, 0.0903 mmol), tetradecane (718 mg), and toluene (6.0 mL) were employed, which demonstrated that the and rate constant for the formation of **H_4-Qin** is 1.69(3) × 10^{-4} (mol m^{-3} s^{-1}).

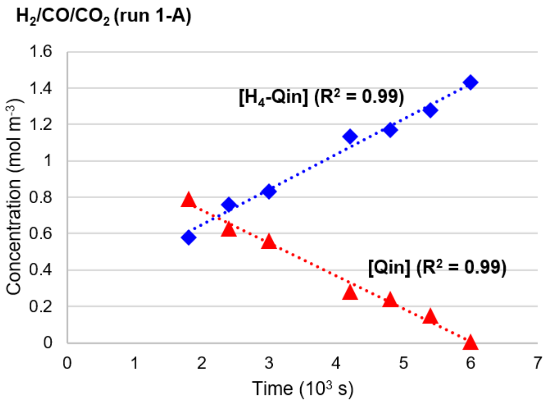

$$-\frac{d[\boldsymbol{Qin}]}{dt} = k_{Qin} = 1.80(9) \times 10^{-4}\ [mol\ m^{-3}\ s^{-1}]$$

$$\frac{d[\boldsymbol{H_4 - Qin}]}{dt} = k_{H4-Qin} = 1.93(10) \times 10^{-4}\ [mol\ m^{-3}\ s^{-1}]$$

Each gas was pressurized at 10 atm.

Fig S23. Time-concentration profiles for run 1-A.

Run 1-B using B^9: Followed by the general procedures using H_2 (10 atm), we independently conducted two experiments to confirm the reproducibility. In the first experiment, **Qin** (1.29 g, 9.01 mmol), **B^9** (62.8 mg, 0.0899 mmol), tetradecane (694 mg), and toluene (6.0 mL) were employed, which demonstrated that the rate constant for the formation of **H_4-Qin** is 2.21(13) × 10^{-4} (mol m^{-3} s^{-1}) as shown in Fig.S24. In the second, **Qin** (1.29 g, 9.01 mmol), **B^9** (62.9 mg, 0.0900 mmol), tetradecane (716 mg), and toluene (6.0 mL) were employed, which demonstrated that the rate constant for the formation of **H_4-Qin** is 1.82(8) × 10^{-4} (mol m^{-3} s^{-1}).

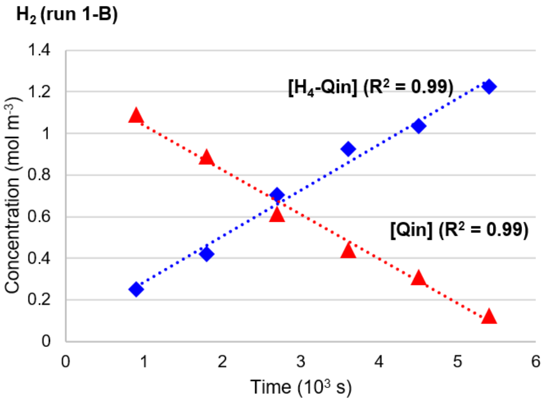

$$-\frac{d[\boldsymbol{Qin}]}{dt} = k_{\boldsymbol{Qin}} = 2.14(12) \times 10^{-4}\ [mol\ m^{-3}\ s^{-1}]$$

$$\frac{d[\boldsymbol{H_4 - Qin}]}{dt} = k_{H4-\boldsymbol{Qin}} = 2.21(13) \times 10^{-4}\ [mol\ m^{-3}\ s^{-1}]$$

H_2 was pressurized at 10 atm.

Fig S24. Time-concentration profiles for run 1-B.

Run 2-A using B[1]: Followed by the general procedures using $H_2/CO/CO_2$ (10 atm each), **Qin** (1.07 g, 7.47 mmol), **B[1]** (38.4 mg, 0.0750 mmol), tetradecane (605 mg), and toluene (5.0 mL) were employed, which demonstrated that the rate constant for the formation of **H_4-Qin** is 3.56(60) × 10^{-6} (mol m^{-3} s^{-1}) as shown in Fig.S25.

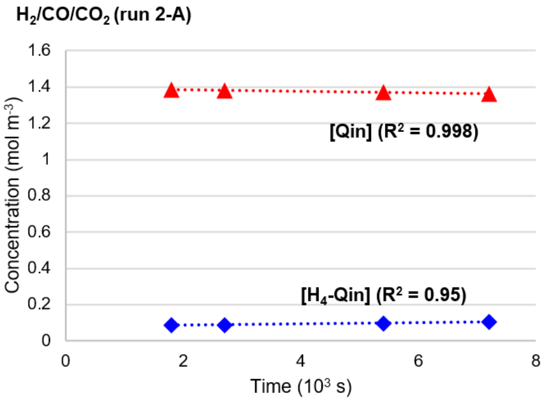

$$-\frac{d[\boldsymbol{Qin}]}{dt} = k_{\boldsymbol{Qin}} = 4.51(15) \times 10^{-6}\ [mol\ m^{-3}\ s^{-1}]$$

$$\frac{d[\boldsymbol{H_4 - Qin}]}{dt} = k_{H4-\boldsymbol{Qin}} = 3.56(60) \times 10^{-6}\ [mol\ m^{-3}\ s^{-1}]$$

Each gas was pressurized at 10 atm.

Fig S25. Time-concentration profiles for run 2-B.

Run 2-B using B[1]: Followed by the general procedures using H_2 (10 atm), **Qin** (1.07 g, 7.47 mmol), **B[1]** (38.4 mg, 0.0750 mmol), tetradecane (608 mg), and toluene (5.0 mL) were employed, which demonstrated that the rate constant for the formation of **H_4-Qin** is 3.08(29) × 10^{-4} (mol m^{-3} s^{-1}) as shown in Fig.S26.

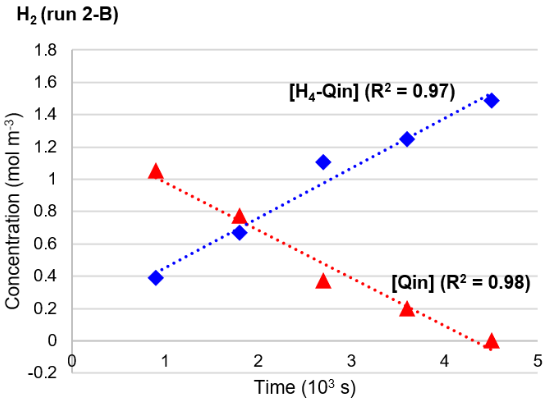

$$-\frac{d[\boldsymbol{Qin}]}{dt} = k_{\boldsymbol{Qin}} = 2.97(26) \times 10^{-4}\ [mol\ m^{-3}\ s^{-1}]$$

$$\frac{d[\boldsymbol{H_4 - Qin}]}{dt} = k_{H4-\boldsymbol{Qin}} = 3.08(29) \times 10^{-4}\ [mol\ m^{-3}\ s^{-1}]$$

H_2 was pressurized at 10 atm.

Fig S26. Time-concentration profiles for run 2-B.

Run 3-A using B[7]: Followed by the general procedures using $H_2/CO/CO_2$ (10 atm each), **Qin** (1.07 g, 7.47 mmol), **B**[7] (39.1 mg, 0.0751 mmol), tetradecane (606 mg), and toluene (5.0 mL) were employed, which demonstrated that the rate constant for the formation of **H_4-Qin** is 1.66(5) × 10^{-4} (mol m^{-3} s^{-1}) as shown in Fig.S27.

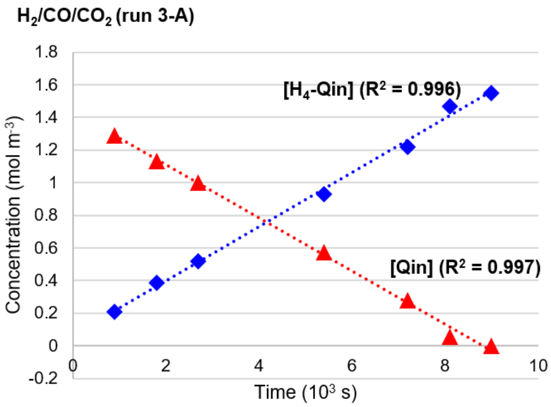

$$-\frac{d[\boldsymbol{Qin}]}{dt} = k_{\boldsymbol{Qin}} = 1.63(4) \times 10^{-4}\ [mol\ m^{-3}\ s^{-1}]$$

$$\frac{d[\boldsymbol{H_4 - Qin}]}{dt} = k_{\boldsymbol{H4-Qin}} = 1.66(5) \times 10^{-4}\ [mol\ m^{-3}\ s^{-1}]$$

Each gas was pressurized at 10 atm.

Fig S27. Time-concentration profiles for run 3-A.

Run 3-B using B[7]: Followed by the general procedures using H_2 (10 atm), **Qin** (1.07 g, 7.47 mmol), **B**[7] (39.1 mg, 0.0751 mmol), tetradecane (604 mg), and toluene (5.0 mL) were employed, which demonstrated that the rate constant for the formation of **H_4-Qin** is 1.71(6) × 10^{-4} (mol m^{-3} s^{-1}) as shown in Fig.S28.

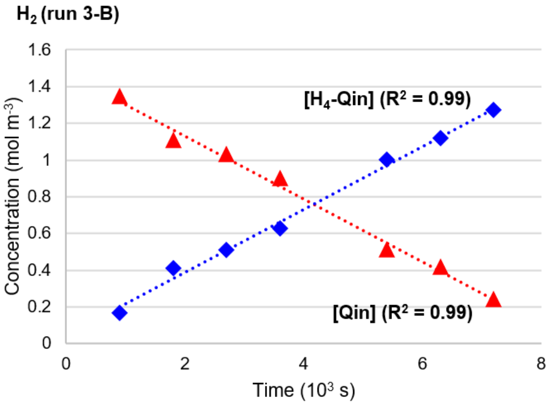

$$-\frac{d[\boldsymbol{Qin}]}{dt} = k_{\boldsymbol{Qin}} = 1.71(7) \times 10^{-4}\ [mol\ m^{-3}\ s^{-1}]$$

$$\frac{d[\boldsymbol{H_4 - Qin}]}{dt} = k_{\boldsymbol{H4-Qin}} = 1.71(6) \times 10^{-4}\ [mol\ m^{-3}\ s^{-1}]$$

H_2 was pressurized at 10 atm.

Fig S28. Time-concentration profiles for run 3-B.

17-2. Influences of gas composition in B[1]-catalyzed hydrogenation of Qin

A mixture of **Qin** (429.6 mg, 3.0 mmol, 1.5 M), **B**[1] (15.4 mg, 0.030 mmol, [**B**[1] = 0.015 M]), and tetradecane (an internal standard) in toluene was prepared and equally divided into 4 autoclave reactors (*V* = 10 mL). Once sealed, each reactor was pressurized with H_2 (10 atm), H_2/CO (10 atm each), H_2/CO_2 (10 atm each), and $H_2/CO/CO_2$ (10 atm each) and heated at 100 °C for 2 h. After degassed at room temperature, the yield of **H_4-Qin** was determined by GC analysis. We repeated these experiments at least twice for each gas composition in order to confirm their reproducibility.

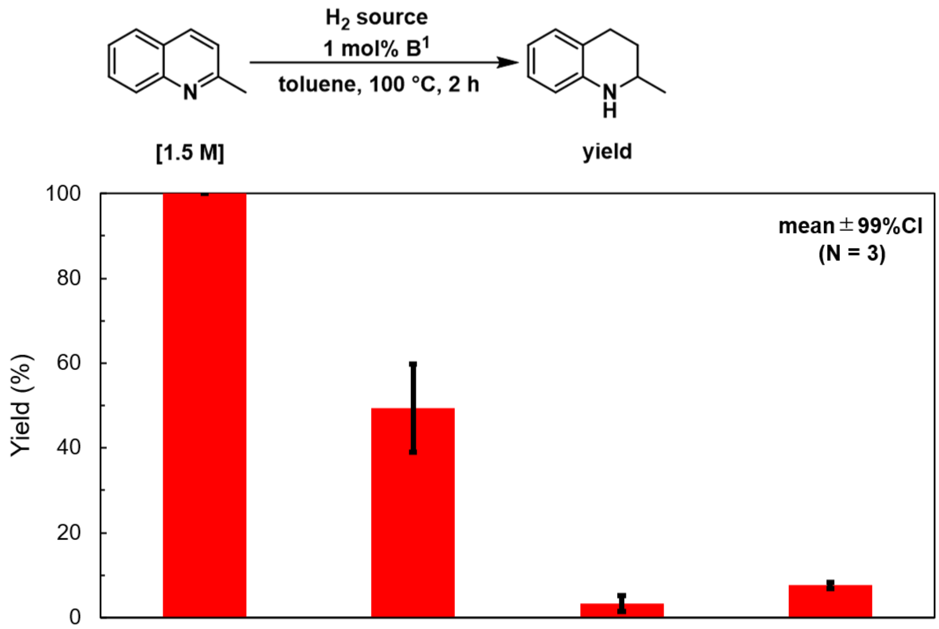

Gas (atm)	H_2 (10)	H_2/CO (10/10)	H_2/CO_2 (10/10)	$H_2/CO/CO_2$ (10/10/10)
run 1	100	48	4	8
run2	100	52	3	7
run 3	-	48	3	8
average	100	49	3	8
standard error	0	1	0.2	0.09
standard deviation	0	1.8	0.3	0.1
99% confidencial interval	-	10	2	1

Fig S29. Influences of gas composition in B^1-catalyzed hydrogenation of Qin.

17-3. Determination of kinetic order in B^9

General: A mixture of **Qin** (1.5 M), $\mathbf{B^9}$ ([$\mathbf{B^9}$ = 0.0075 M, 0.010 M, 0.0015 M, 0.0225 M, 0.030 M]), and tetradecane (an internal standard) in toluene was prepared and equally divided into 10 autoclave reactors (V = 10 mL). Once sealed, each reactor was pressurized with $H_2/CO/CO_2$ (10 atm each) and heated at 100 °C. The conversion of **Qin** and the yield of **H_4-Qin** were monitored by GC analysis. These experiments were repeated twice to confirm the reproducibility.

Run 1: Followed by the general procedures, we independently conducted two experiments to confirm the reproducibility. In the first experiment, **Qin** (2.15 g, 15.0 mmol), $\mathbf{B^9}$ (52.4 mg, 0.0750 mmol), tetradecane (1.20 g), and toluene (10 mL) were employed, which demonstrated that the rate constant for the formation of **H_4-Qin** is 1.29(5) × 10^{-4} (mol m^{-3} s^{-1}) as shown in Fig.S30. In the second, **Qin** (2.16 g, 15.1 mmol), $\mathbf{B^9}$ (52.4 mg, 0.0750 mmol), tetradecane (1.20 g), and toluene (10 mL) were employed, which demonstrated that the and rate constant for the formation of **H_4-Qin** is 1.19(5) × 10^{-4} (mol m^{-3} s^{-1}).

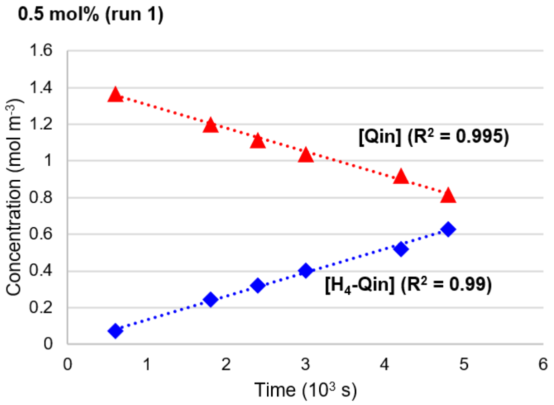

$$-\frac{d[\boldsymbol{Qin}]}{dt} = k_{\boldsymbol{Qin}} = 1.27(4) \times 10^{-4}\ [mol\ m^{-3}\ s^{-1}]$$

$$\frac{d[\boldsymbol{H_4-Qin}]}{dt} = k_{\boldsymbol{H4-Qin}} = 1.29(5) \times 10^{-4}\ [mol\ m^{-3}\ s^{-1}]$$

Fig S30. Time-concentration profiles for run 1.

Run 2: Followed by the general procedures, we independently conducted two experiments to confirm the reproducibility. In the first experiment, **Qin** (1.43 g, 9.99 mmol), **B**[9] (52.4 mg, 0.0750 mmol), tetradecane (806 mg), and toluene (6.7 mL) were employed and, which demonstrated that the rate constant for the formation of **H_4-Qin** is 2.13(8) × 10^{-4} (mol m^{-3} s^{-1}) as shown in Fig.S31. In the second, **Qin** (1.43 g, 9.99 mmol), **B**[9] (52.3 mg, 0.0749 mmol), tetradecane (798 mg), and toluene (6.7 mL) were employed and, which demonstrated that the rate constant for the formation of **H_4-Qin** is 2.09(11) × 10^{-4} (mol m^{-3} s^{-1}).

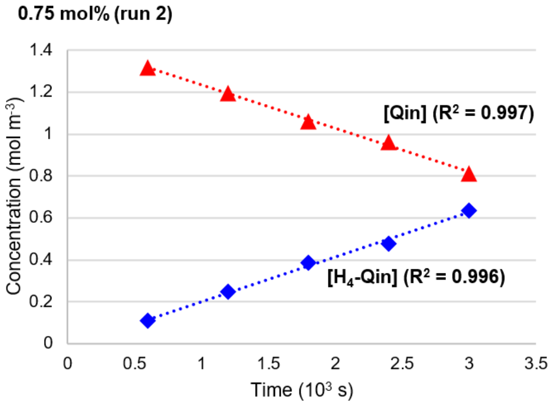

$$-\frac{d[\boldsymbol{Qin}]}{dt} = k_{\boldsymbol{Qin}} = 2.08(7) \times 10^{-4}\ [mol\ m^{-3}\ s^{-1}]$$

$$\frac{d[\boldsymbol{H_4-Qin}]}{dt} = k_{\boldsymbol{H4-Qin}} = 2.13(8) \times 10^{-4}\ [mol\ m^{-3}\ s^{-1}]$$

Fig S31. Time-concentration profiles for run 2.

Run 3: Followed by the general procedures, we independently conducted two experiments to confirm the reproducibility. In the first experiment, **Qin** (862 mg, 6.02 mmol), **B**[9] (63.0 mg, 0.0902 mmol), tetradecane (481 mg), and toluene (4.0 mL) were employed and, which demonstrated that the rate constant for the formation of **H_4-Qin** is 5.76(51) × 10^{-4} (mol m^{-3} s^{-1}) as shown in Fig.S32. In the second, **Qin** (859 mg, 6.00 mmol), **B**[9] (62.9 mg, 0.0900 mmol), tetradecane (485 mg), and toluene (4.0 mL) were employed and, which demonstrated that the rate constant for the formation of **H_4-Qin** is 4.93(47) × 10^{-4} (mol m^{-3} s^{-1}).

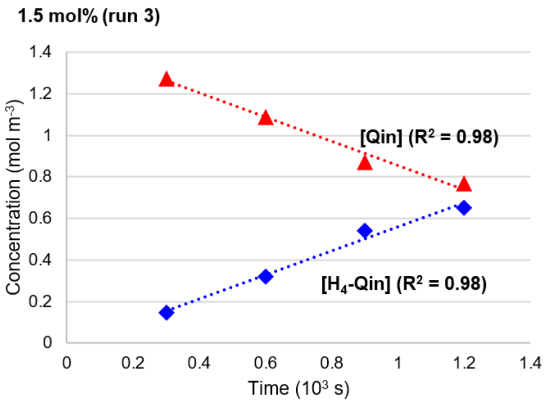

$$-\frac{d[\boldsymbol{Qin}]}{dt} = k_{\boldsymbol{Qin}} = 5.80(56) \times 10^{-4}\ [mol\ m^{-3}\ s^{-1}]$$

$$\frac{d[\boldsymbol{H_4 - Qin}]}{dt} = k_{H4-\boldsymbol{Qin}} = 5.76(51) \times 10^{-4}\ [mol\ m^{-3}\ s^{-1}]$$

Fig S32. Time-concentration profiles for run 3.

Run 4: Followed by the general procedures, we independently conducted two experiments to confirm the reproducibility. In the first experiment, **Qin** (644 mg, 4.50 mmol), **B**[9] (62.8 mg, 0.0899 mmol), tetradecane (370 mg), and toluene (3.0 mL) were employed and, which demonstrated that the rate constant for the formation of **H_4-Qin** is 8.57(66) × 10^{-4} (mol m^{-3} s^{-1}) as shown in Fig.S33. In the second, **Qin** (647 mg, 4.52 mmol), **B**[9] (62.8 mg, 0.0899 mmol), tetradecane (360 mg), and toluene (3.0 mL) were employed and, which demonstrated that the rate constant for the formation of **H_4-Qin** is 9.36(117) × 10^{-4} (mol m^{-3} s^{-1}).

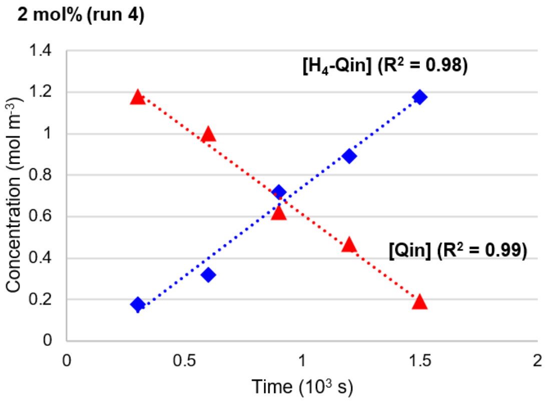

$$-\frac{d[\boldsymbol{Qin}]}{dt} = k_{\boldsymbol{Qin}} = 8.35(58) \times 10^{-4}\ [mol\ m^{-3}\ s^{-1}]$$

$$\frac{d[\boldsymbol{H_4 - Qin}]}{dt} = k_{H4-\boldsymbol{Qin}} = 8.57(66) \times 10^{-4}\ [mol\ m^{-3}\ s^{-1}]$$

Fig S33. Time-concentration profiles for run 4.

Order in B[9]: From these results, a plot of k_{int} vs. [**B**[9]] and the corresponding log-log profile are given (Fig S34), suggesting the first order dependence on **B**[9].

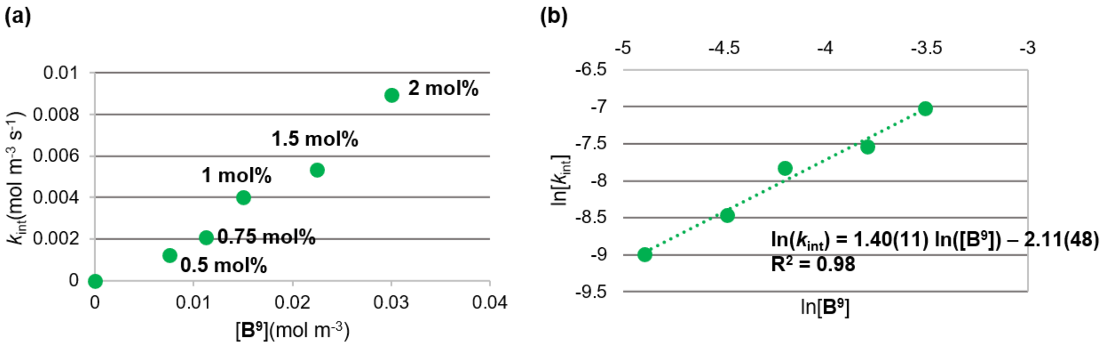

Fig S34. Order in B[9]**.** (a) Kinetic order in [B[9]]. (b) A profile of lnk_{int} with respect to ln[B[9]].

17-4. Determination of kinetic order in B[7]

General: A mixture of **Qin** (1.5 M), **B**[7] ([**B**[7] = 0.030 M, 0.045 M]), and tetradecane (an internal standard) in toluene was prepared and equally divided into 8 (run 1) or 4 (run 2) autoclave reactors (V = 10 mL). Once sealed, each reactor was pressurized with H_2/CO/CO_2 (10 atm each) heated at 100 °C. The conversion of **Qin** and the yield of **H_4-Qin** were monitored by GC analysis, resulting into the determination of the initial rate constant k_{obs}.

Run 1: Followed by the general procedures, **Qin** (860 mg, 6.01 mmol), **B**[7] (62.5 mg, 0.120 mmol), tetradecane (494 mg), and toluene (4 mL) were employed, which demonstrated that the rate constant for the formation of **H_4-Qin** generation is 4.23(50) × 10^{-4} (mol m^{-3} s^{-1}) as shown in Fig.S35.

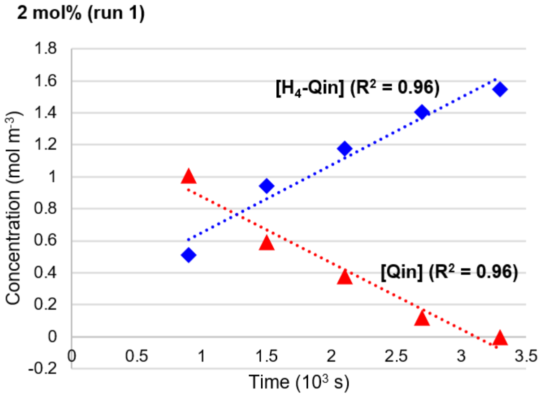

$$-\frac{d[Qin]}{dt} = k_{Qin} = 4.14(47) \times 10^{-4}\ [mol\ m^{-3}\ s^{-1}]$$

$$\frac{d[H_4 - Qin]}{dt} = k_{H4-Qin} = 4.23(50) \times 10^{-4}\ [mol\ m^{-3}\ s^{-1}]$$

Fig S35. Time-concentration profiles for run 1.

Run 2: Followed by the general procedures, **Qin** (430 mg, 3.00 mmol), **B**[7] (46.9 mg, 0.0901 mmol), tetradecane (248.7 mg), and toluene (2 mL) were employed, which demonstrated that the rate constant for the formation of **H_4-Qin** generation is 6.19(32) × 10^{-4} (mol m^{-3} s^{-1}) as shown in Fig.S36.

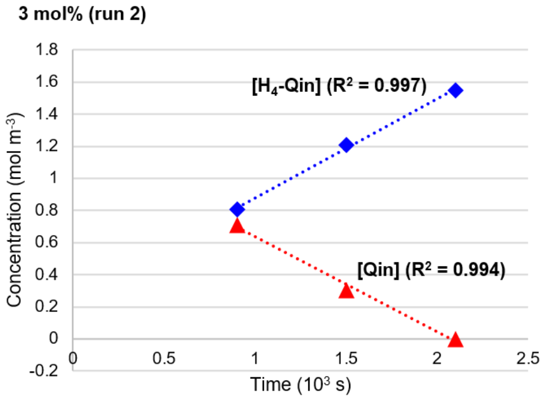

$$-\frac{d[\boldsymbol{Qin}]}{dt} = k_{\boldsymbol{Qin}} = 5.93(47) \times 10^{-4}\ [mol\ m^{-3}\ s^{-1}]$$

$$\frac{d[\boldsymbol{H_4} - \boldsymbol{Qin}]}{dt} = k_{\boldsymbol{H4-Qin}} = 6.19(32) \times 10^{-4}\ [mol\ m^{-3}\ s^{-1}]$$

Fig S36. Time-concentration profiles for run 2.

Order in B^7: From these results, a plot of k_{obs} vs. [**B^7**] and the corresponding log-log profile are given (Fig S37), suggesting the first order dependence on **B^7**.

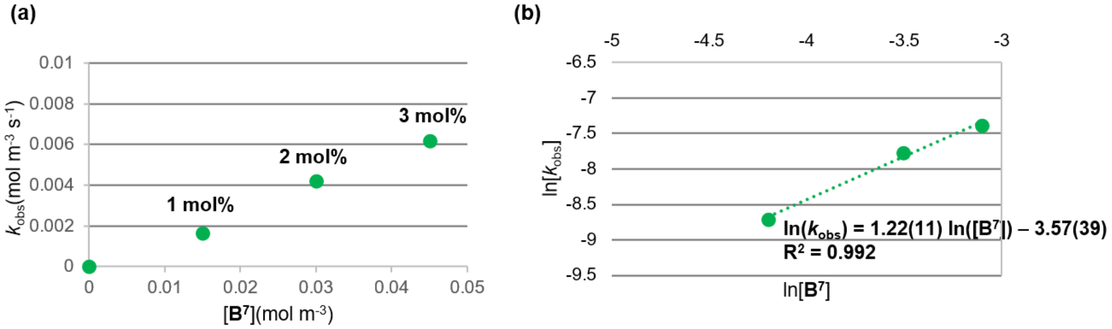

Fig S37. Order in B^7. (a) Kinetic order in [B^7]. (b) A profile of lnk_{obs} with respect to ln[B^7].

17-5. Reaction of Qin with B^1 in the presence of H_2/CO_2

A pressure-tight NMR tube (Wilmad-LabGrass 542-PV-7; V = 1.8 mL) was charged with **Qin** (15.0 mg, 0.105 mmol), **B^1** (25.6 mg, 0.0500 mmol) and α,α,α-trifluorotoluene (23.9 mg, 0.164 mmol; an internal standard). Once sealed, the NMR tube was pressurized with H_2/CO_2 (2.5 atm each), followed by heating at 100 °C for 3 h. Then, the ^{1}H, ^{11}B, and ^{19}F NMR analyses were conducted. X-ray data for **H_4-Qin–B^1** (Fig S38d): M = 659.20, colorless, monoclinic, $P2_1/n$ (#14), a = 8.5215(2) Å, b = 14.9248(3) Å, c = 20.1080(3) Å, α = 90°, β = 100.571(2)°, γ = 90°, V = 2513.97(9) Å^3, Z = 4, $Dcalcd$ = 1.742 g/cm^3, T = −150 °C, R_1 (wR_2) = 0.0280 (0.0765).

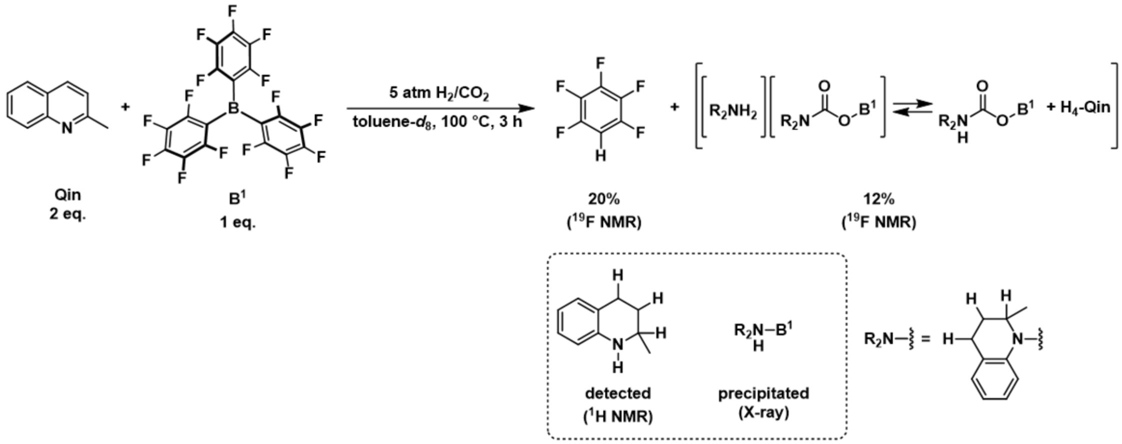
Qin
2 eq.
B1
1 eq.
5 atm H2/CO2
toluene-d8, 100 °C, 3 h
20%
(19F NMR)
12%
(19F NMR)
R2NH2
R2N
+ H4-Qin
detected
(1H NMR)
R2N–B1
precipitated
(X-ray)
R2N– =

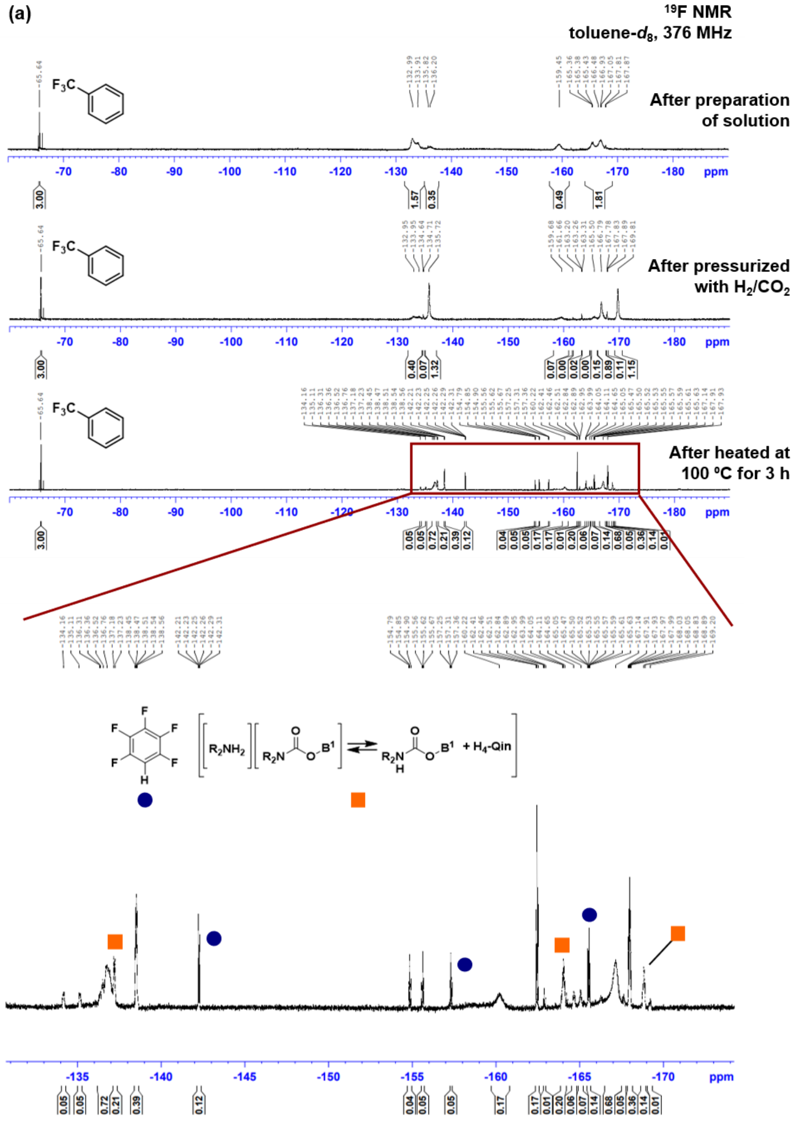
(a)
19F NMR
toluene-d8, 376 MHz
F3C
After preparation
of solution
After pressurized
with H2/CO2
After heated at
100 ºC for 3 h
ppm
+ H4-Qin

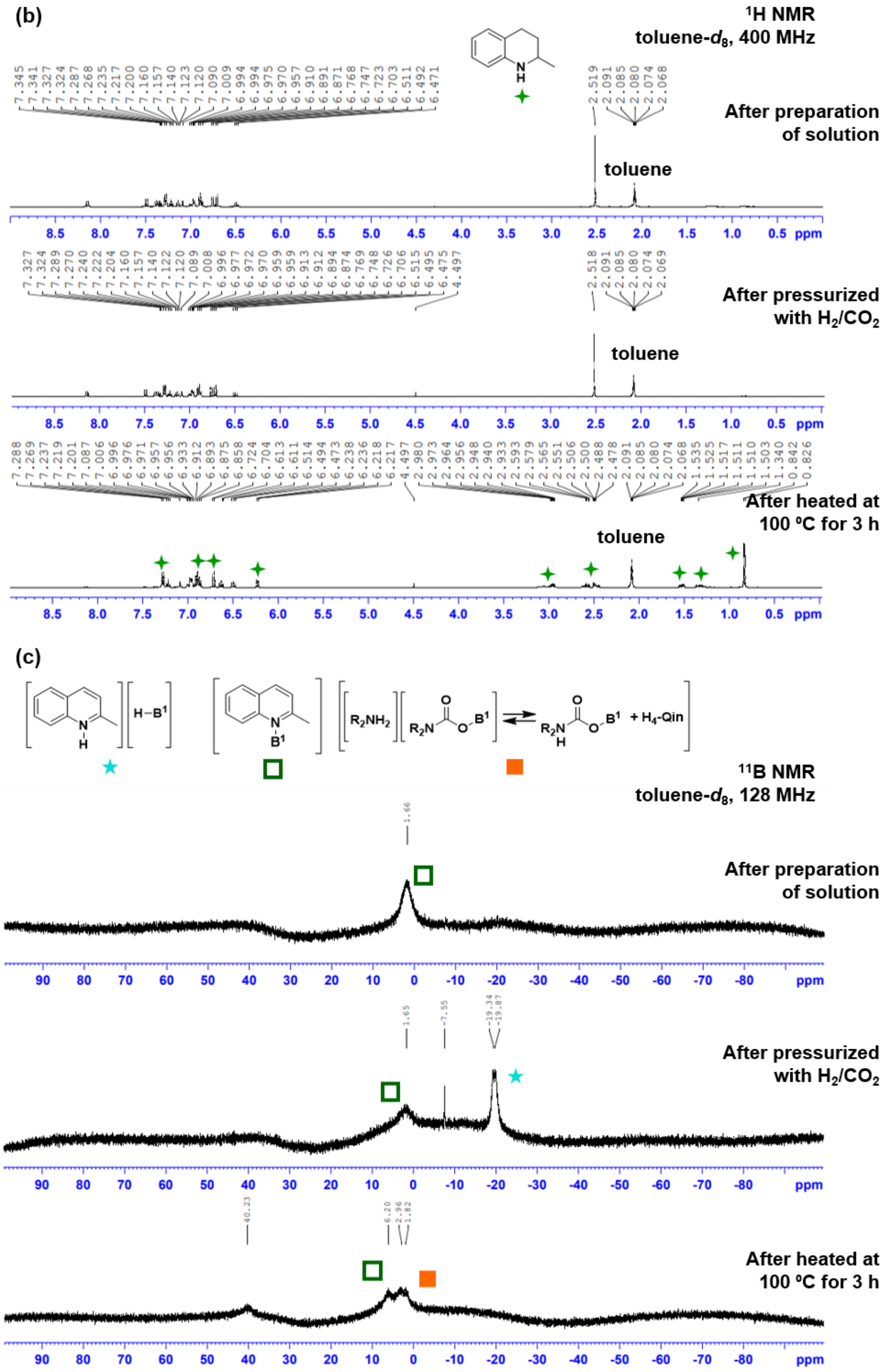

(b)
^{1}H NMR
toluene-d_8, 400 MHz
After preparation of solution
toluene
After pressurized with H_2/CO_2
toluene
After heated at 100 ºC for 3 h
toluene
ppm
(c)
H–B^1
R_2NH_2
R_2N
R_2N H
+ H_4-Qin
^{11}B NMR
toluene-d_8, 128 MHz
After preparation of solution
After pressurized with H_2/CO_2
After heated at 100 ºC for 3 h
ppm

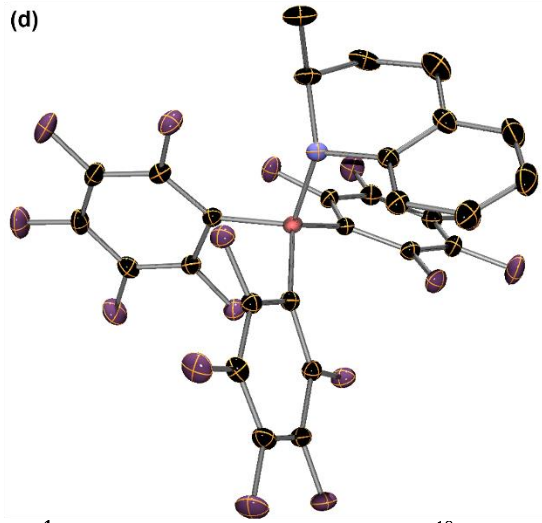

Fig S38. Reaction of Qin with B[1] in the presence of H_2/CO_2. (a) ^{19}F NMR spectra. (b) ^{1}H NMR spectra. (c) ^{11}B NMR spectra. (d) Molecular structure of H_4-Qin–B[1] with ellipsoids set at 30% probability; H atoms are omitted for clarity.

17-6. Reaction of Qin with B[9] in the presence of H_2/CO_2

A pressure-tight NMR tube (Wilmad-LabGrass 542-PV-7; V = 1.8 mL) was charged with **Qin** (13.9 mg, 0.0971 mmol), **B[9]** (34.8 mg, 0.0498 mmol) and α,α,α-trifluorotoluene (24.0 mg, 0.164 mmol; an internal standard). Once sealed, the NMR tube was pressurized with H_2/CO_2 (2.5 atm each), followed by heating at 100 °C for 3 h. Then the ^{1}H, ^{11}B, and ^{19}F NMR analyses were conducted.

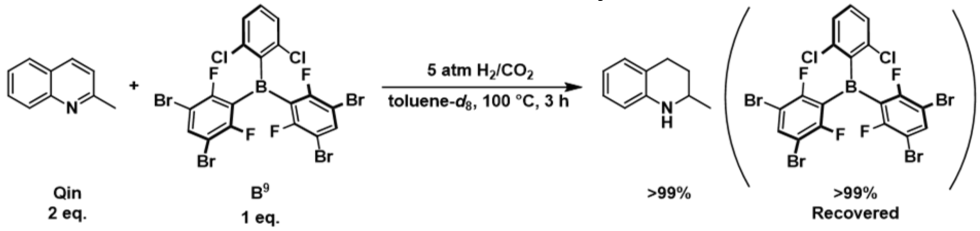

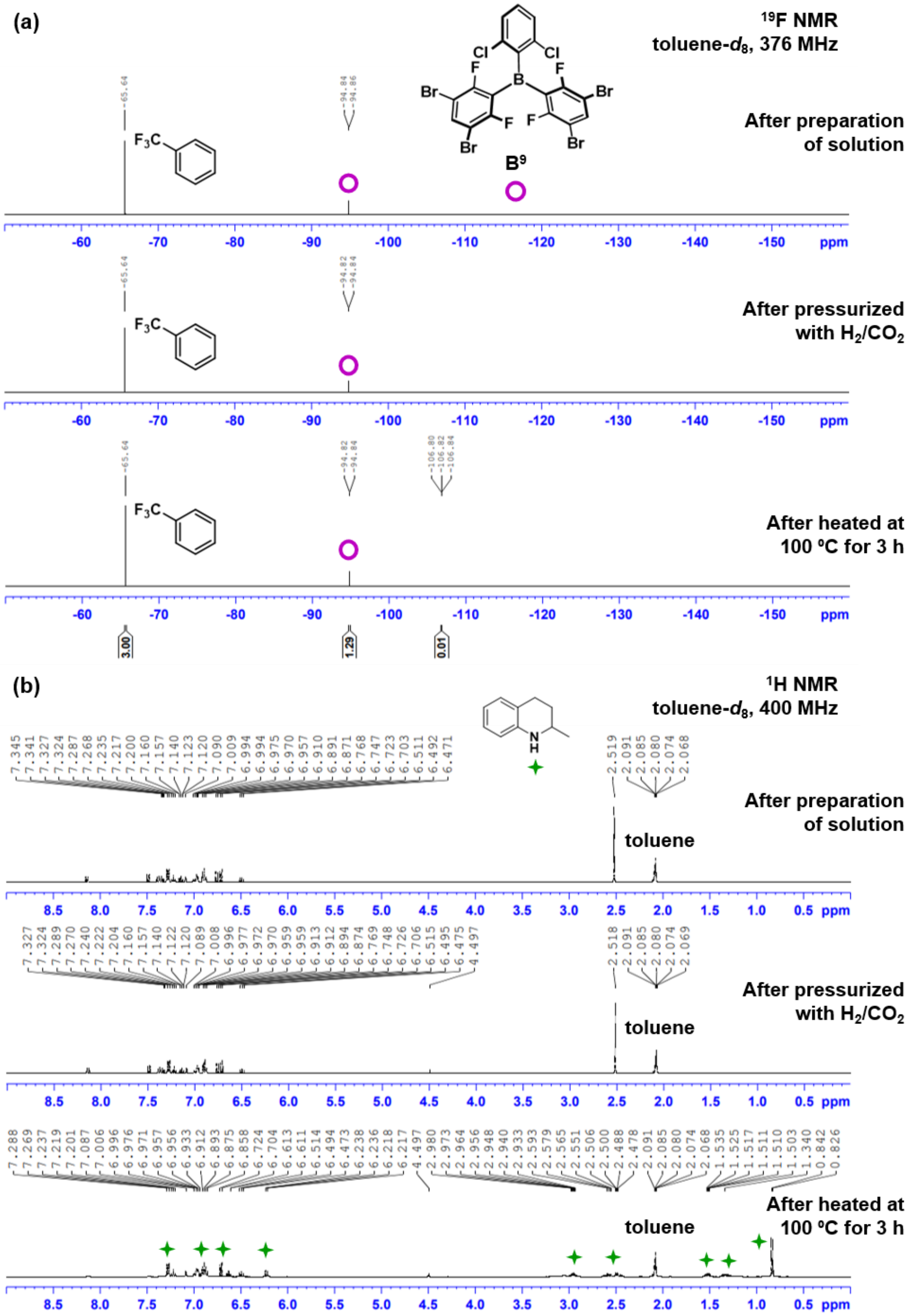
(a)
19F NMR
toluene-d8, 376 MHz
B9
After preparation of solution
After pressurized with H2/CO2
After heated at 100 ºC for 3 h
-65.64
-94.84
-94.86
-94.82
-106.80
-106.82
-106.84
3.00
1.29
0.01
ppm
(b)
1H NMR
toluene-d8, 400 MHz
toluene
ppm

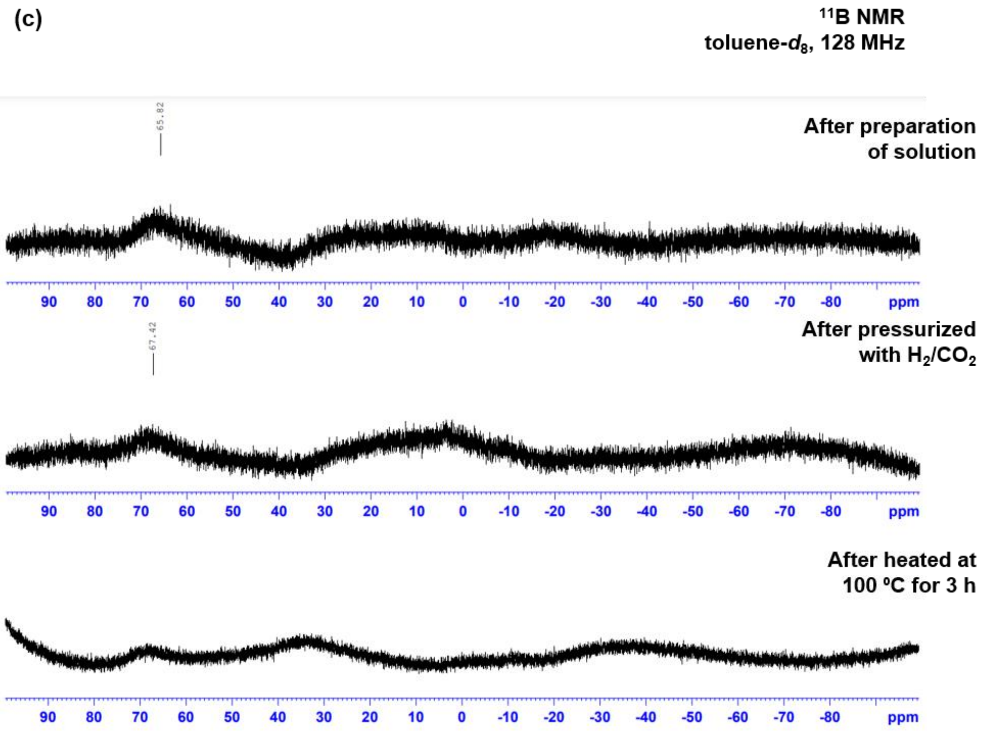

Fig S39. Reaction of Qin with B^9 in the presence of H_2/CO_2. (a) ^{19}F NMR spectra. (b) ^{1}H NMR spectra. (C) ^{11}B NMR spectra.

17-7. Reaction of H_4-Qin with B^1 in the presence of CO_2

A pressure-tight NMR tube (Wilmad-LabGrass 542-PV-7; V = 1.8 mL) was charged with **H_4-Qin** (14.3 mg, 0.0971 mmol), and **B^1** (25.6 mg, 0.0500 mmol). Once sealed, the NMR tube was pressurized with CO_2 (5 atm), followed by heating at 100 °C for 1 h. Then, the ^{1}H, ^{11}B, and ^{19}F NMR analyses were conducted.

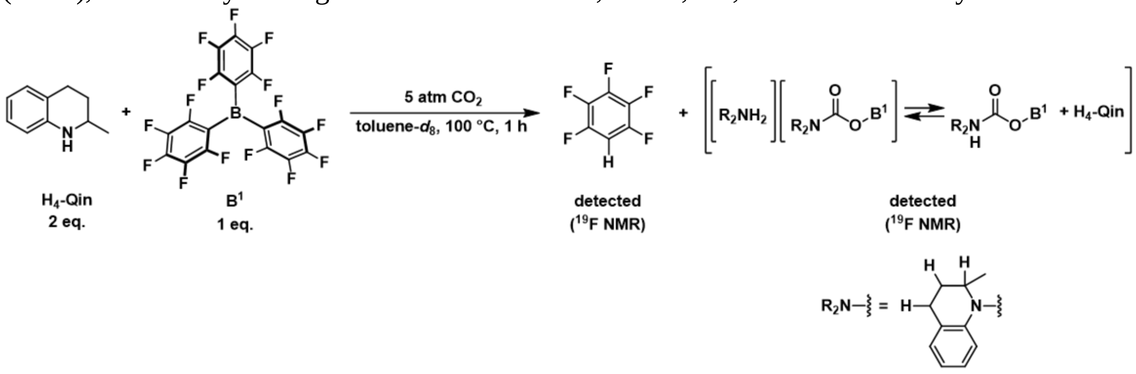

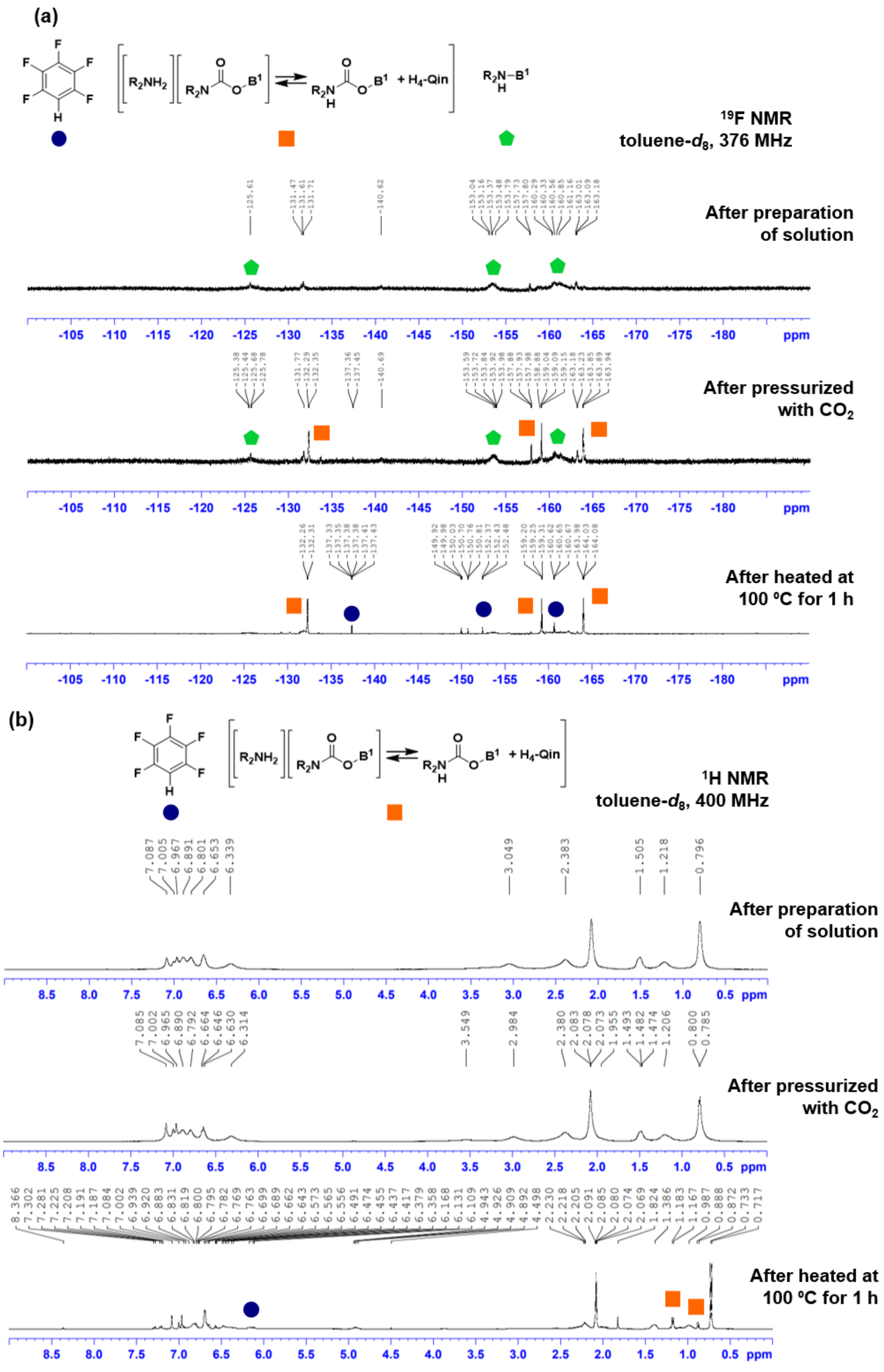

(a)
R2NH2
R2N
O
B1
+ H4-Qin
R2N-B1
H
19F NMR
toluene-d8, 376 MHz
After preparation of solution
After pressurized with CO2
After heated at 100 ºC for 1 h
ppm
(b)
1H NMR
toluene-d8, 400 MHz
After preparation of solution
After pressurized with CO2
After heated at 100 ºC for 1 h
ppm

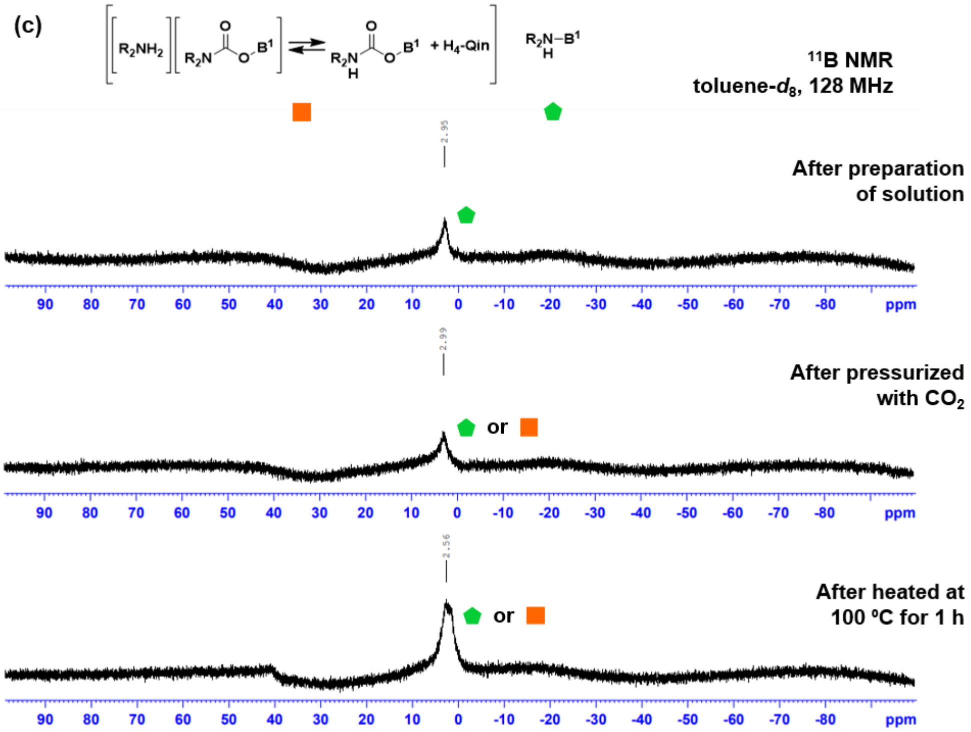

Fig S40. Reaction of H_4-Qin with B[1] in the presence of CO_2. (a) ^{19}F NMR spectra. (b) ^{1}H NMR spectra. (C) ^{11}B NMR spectra.

17-8. Reaction of [H_4-Qin–B[1]] with H_2 followed by CO_2

A pressure-tight NMR tube (Wilmad-LabGlass 542-PV-7; V = 1.8 mL) was charged with **H_4-Qin** (14.8 mg, 0.101 mmol), and **B[1]** (25.6 mg, 0.0500 mmol). Once sealed, the NMR tube was pressurized with H_2 (2.5 atm), followed by heating at 100 °C for 1 h. Then, the ^{1}H, ^{11}B, and ^{19}F NMR analyses were conducted. Next, this NMR tube was pressurized with CO_2 (2.5 atm), followed by heating at 100 °C for 1 h. Then, the ^{1}H, ^{11}B, and ^{19}F NMR analyses were again conducted.

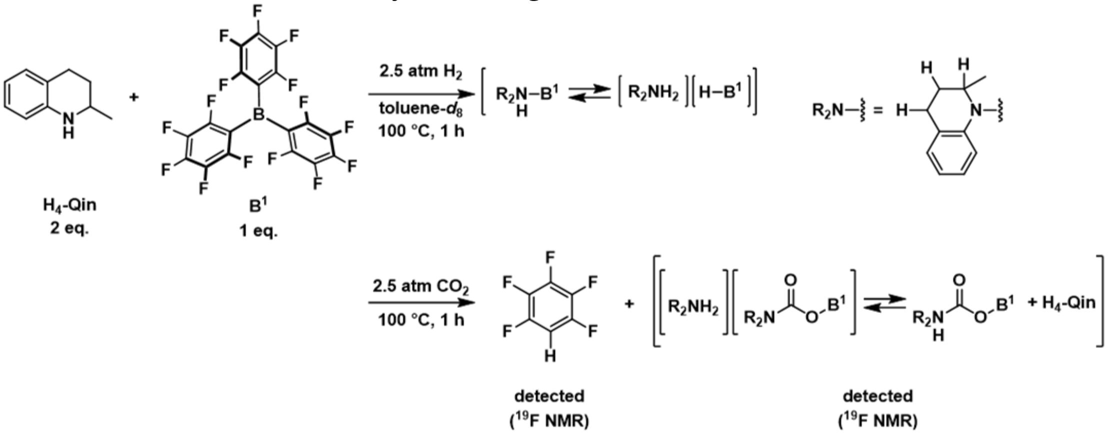

(a)

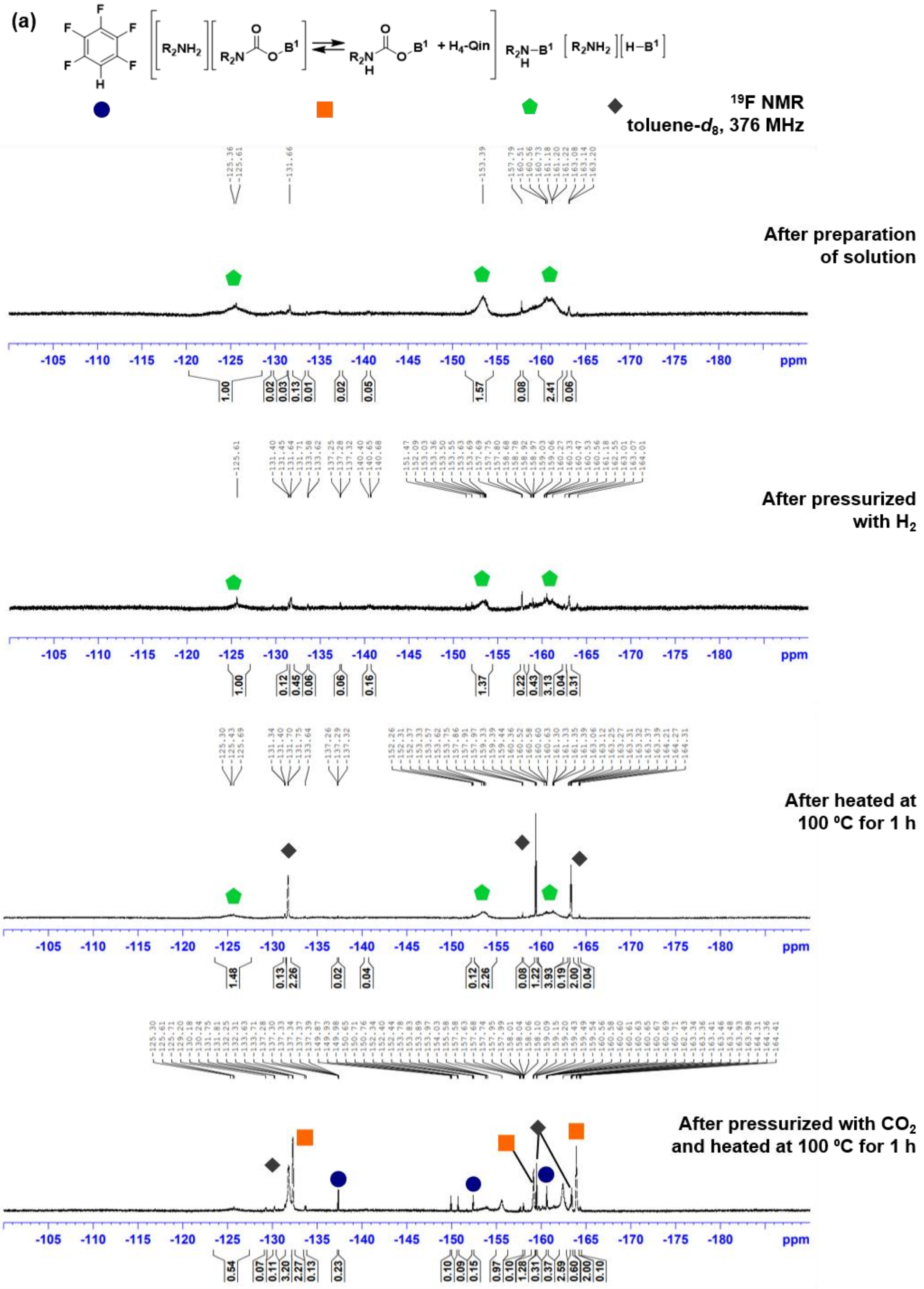

R2NH2
R2N
O
B1
H4-Qin
R2N–B1
H–B1
19F NMR
toluene-d8, 376 MHz
After preparation of solution
After pressurized with H2
After heated at 100 ºC for 1 h
After pressurized with CO2 and heated at 100 ºC for 1 h
ppm

(b)

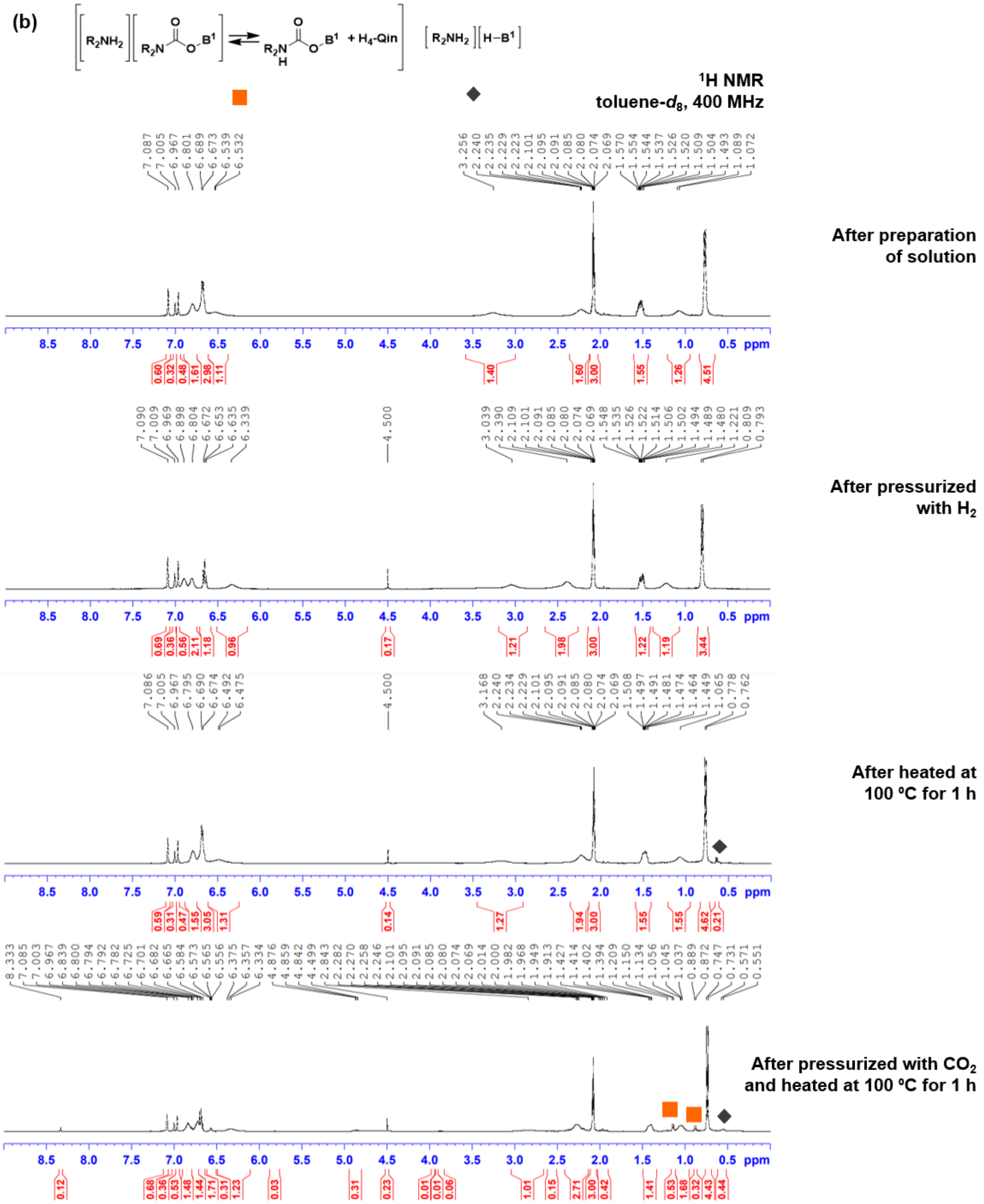
[R2NH2][R2N–C(O)–O–B1] ⇌ R2NH–C(O)–O–B1 + H4-Qin
[R2NH2][H–B1]
1H NMR
toluene-d8, 400 MHz
After preparation of solution
After pressurized with H2
After heated at 100 ºC for 1 h
After pressurized with CO2 and heated at 100 ºC for 1 h
ppm

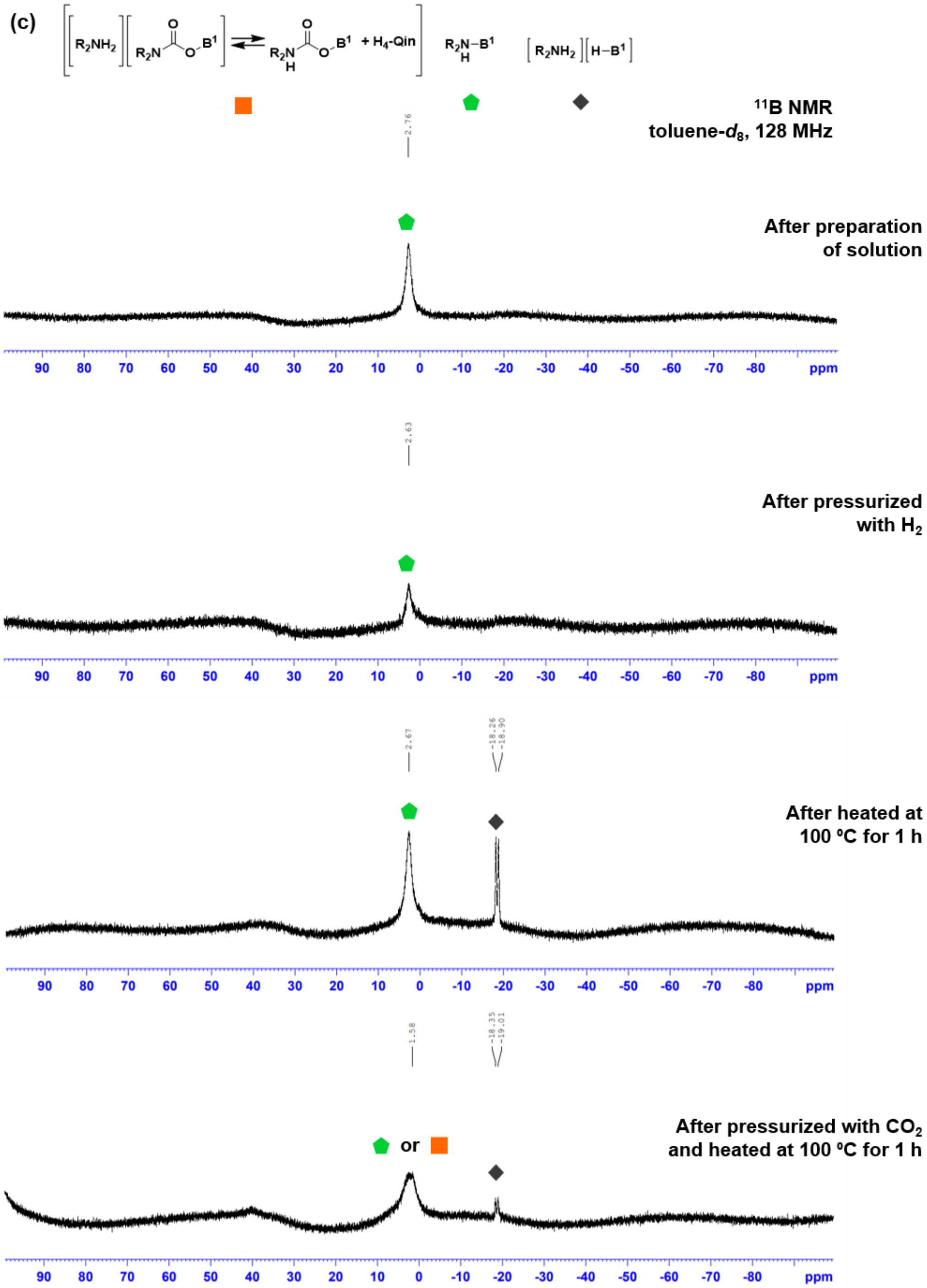

Fig S41. Reaction of [H_4-Qin–B^1] with H_2 followed by CO_2. (a) ^{19}F NMR spectra. (b) ^{1}H NMR spectra. (C) ^{11}B NMR spectra.

17-9. Hydrogenation of Qin using [Qin–H][HO–B^1] or [Qin–H][HO–B^9]

General: A 30 mL autoclave was charged with **Qin** (0.225 mmol), [**Qin**–H][HO–$\mathbf{B^n}$] (0.025 mmol), and tetradecane (an internal standard), and toluene (0.17 mL). Once sealed, the autoclave was pressurized with H_2 (4 atm) and heated at 100 °C for 6 h. After degassed at room temperature, the yield of **H_4-Qin** was determined by GC analysis. The results were shown in Fig 4f.

Use of [Qin–H][HO–B^1]: Followed by the general procedures, **Qin** (31.2 mg), [**Qin**–H][HO–$\mathbf{B^1}$] (16.9 mg), and tetradecane (13.9 mg) were employed, giving **H_4-Qin** in <1% GC yield.

Use of [Qin–H][HO–B^9]: Followed by the general procedures, **Qin** (32.3 mg), [**Qin**–H][HO–$\mathbf{B^9}$] (21.3 mg), and tetradecane (19.6 mg) were employed, giving **H_4-Qin** in 13% GC yield.

[18] Comparison of Lewis acidity among B^n by Gutmann-Beckett method

General: A J. Young tube was charged with $\mathbf{B^n}$ (0.060 mmol), triethylphosphine oxide (8.1 mg, 0.057 mmol), and C_6D_6 (n = 1-9) or CD_2Cl_2 (n = 10). Then, the ^{31}P NMR analyses were conducted. The acceptor number (AN) was calculated according to the literature and summarized in Table S4.[52]

B^n	B^1	B^2	B^3	B^4	B^5
δ_p	76.7	75.6	75.2	71.3	75.4
AN	79	77	76	67	76

B^n	B^6	B^7	B^8	B^9	B^{10}
δ_p	61.8	73.1	71.8	72.6	73.6
AN	46	71	68	70	72

Table S4. Comparison of Lewis acidity based on the Gutmann-Beckett method.

[19] Computational details

The density functional theory (DFT) calculations were performed with Gaussian 16 (Revision C.01) software.[53] All the structures were optimized at the *ω*B97X-D[54] level of theory with the 6-31G(d,p) basis sets. Frequency calculations were performed to verify that intermediates have no imaginary frequency, whereas the transition structures have only one imaginary frequency. The appropriateness of the connections between each reactant and product via a transition state was confirmed using intrinsic reaction coordinate (IRC). Single-point energy calculations were carried out at *ω*B97X-D/6-311+G(d,p)/gas-phase level of theory otherwise noted. A polarizable continuum model (PCM)[55] was adopted, and the parameters for quinoline were used. The reported Gibbs free energies were calculated at 298.15 K. These calculations involve a certain margin of error.

19-1. Structural optimization of $\mathbf{B^n}$ and their LUMO energy levels

In general, optimization of $\mathbf{B^n}$ was carried out based on the geometrical parameters obtained by SC-XRD analysis. The energy levels of LUMO were then calculated by the single point calculation (Fig S42).

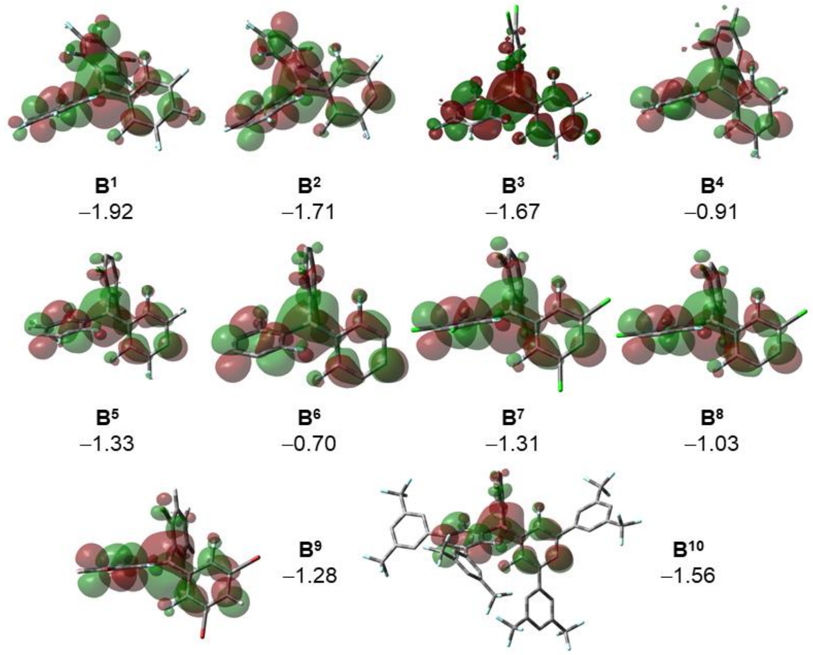

Fig S42. LUMO of $\mathbf{B^n}$ and their energy levels (eV).

19-2. Theoretical investigation of reaction between $\mathbf{B^n}$ and CO

The optimized molecular structure of **OC–$\mathbf{B^n}$** and saddle point species **TS-$\mathbf{B^n}$** (n= 1, 5, 7, 9-10) with their relative Gibbs free energy (ΔG^{o} kcal·mol^{-1}) with respect to [CO + $\mathbf{B^n}$] (+ 0.0 kcal·mol^{-1}) are summarized in Fig S43.

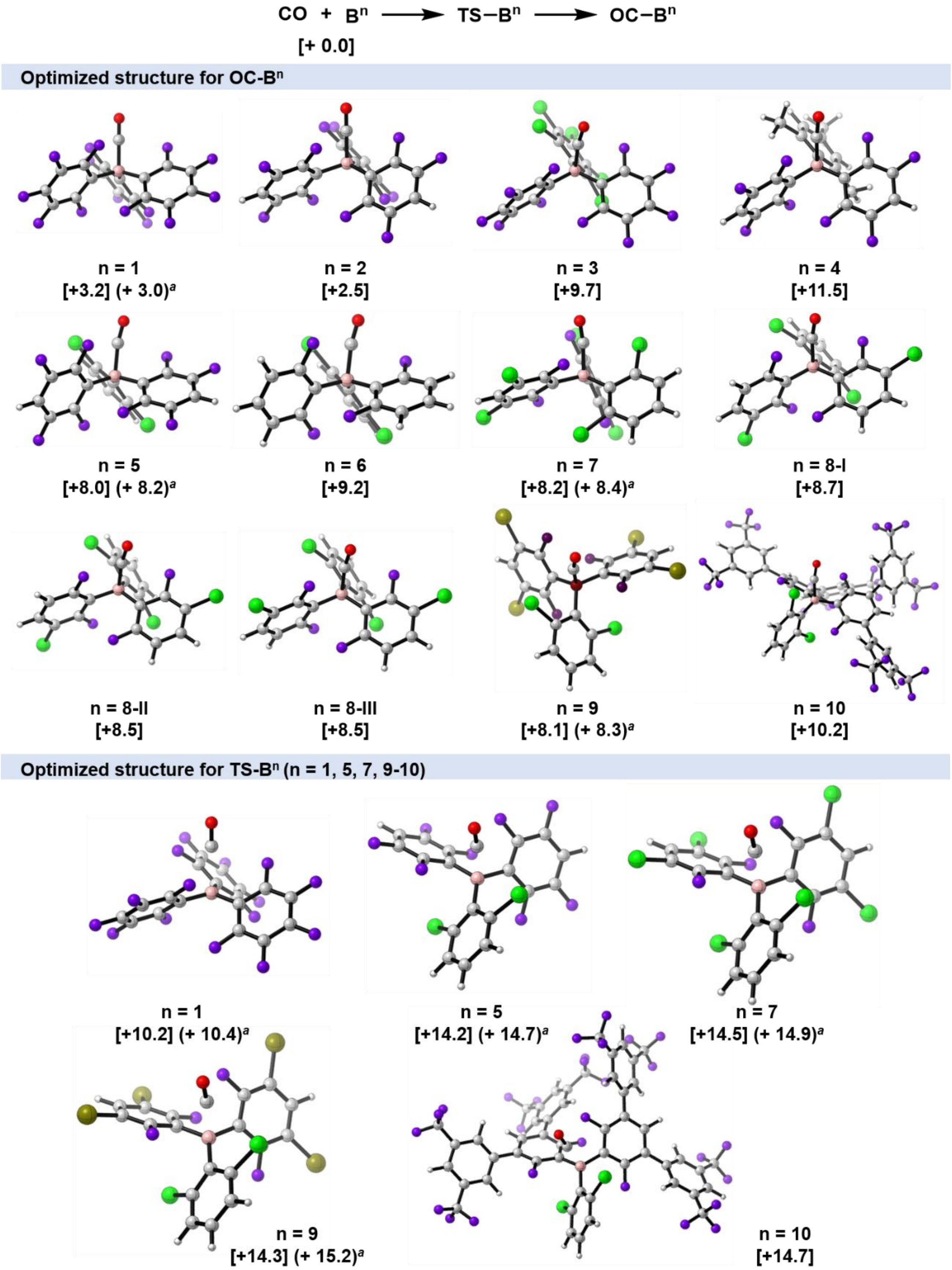

Fig S43. Optimized structure of OC–B^n and saddle point species TS-B^n (n= 1, 5, 7, 9-10). The relative Gibbs free energy (kcal·mol^{-1}) with respect to [CO + B^n] (+ 0.0 kcal·mol^{-1}) is shown. ^{a}SPC results calculated at the ωB97X-D/6-311+G(d,p)/PCM (quinoline) level of theory.

Table S5. Computed Cartesian coordinates (x, y, z) for the optimized structure of CO.

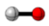

E [a.u.] = −113.27
G [a.u.] = −113.29

C	0	0	-0.648715
O	0	0	0.486542

Table S6. Computed Cartesian coordinates (x, y, z) for the optimized structure of B[1].

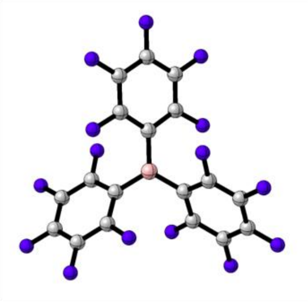

E [a.u.] = −2207.46
G [a.u.] = −2207.52

F	-2.267086	1.059895	1.476880	C	-0.201537	3.806070	-0.750830
F	-3.051356	3.615835	1.471830	C	-1.307153	4.189376	-0.001293
F	-1.701507	5.453383	-0.002311	C	-1.998934	3.246331	0.749265
F	0.454366	4.707502	-1.474261	C	-1.577035	1.925581	0.730986
F	1.262417	2.159175	-1.476005	C	1.528572	-0.343944	0.000830
F	2.051765	1.433039	1.477270	C	2.047754	-1.415511	-0.730390
F	4.657338	0.835242	1.472300	C	3.398466	-1.728443	-0.750281
F	5.574813	-1.252405	-0.001014	C	4.282863	-0.962396	-0.000533
F	3.851556	-2.746971	-1.473813	C	3.811511	0.108144	0.749747
F	1.240688	-2.173358	-1.476173	C	2.456683	0.402723	0.731326
F	0.214226	-2.493705	1.478785	C	-1.061680	-1.151936	0.001329
F	-1.608217	-4.449951	1.474251	C	-2.248965	-1.065120	-0.730276
F	-3.873885	-4.199470	-0.000154	C	-3.196118	-2.077640	-0.750124
F	-4.304410	-1.960237	-1.474074	C	-2.975957	-3.226374	0.000234
F	-2.500528	0.012935	-1.476216	C	-1.813557	-3.354031	0.751038
C	-0.466341	1.494770	0.000705	C	-0.880122	-2.328820	0.732355
C	0.202437	2.479765	-0.730649	B	0.000260	-0.000487	0.001212

Table S7. Computed Cartesian coordinates (x, y, z) for the optimized structure of B[2].

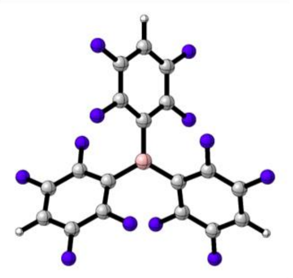

E [a.u.] = −1909.86
G [a.u.] = −1909.92

F	-2.234679	1.061493	1.514382
F	-3.015952	3.610394	1.509291
F	0.432720	4.681292	-1.515470
F	1.235019	2.139110	-1.517467
F	2.032637	1.403827	1.518473
F	4.631247	0.809427	1.515897
F	3.842449	-2.712173	-1.511458
F	1.238926	-2.139542	-1.515849
F	0.203128	-2.469721	1.509029
F	-1.611686	-4.421782	1.505295
F	-4.276400	-1.965581	-1.503842
F	-2.477868	0.001623	-1.507564
C	-0.467091	1.496007	-0.001130
C	0.189451	2.471695	-0.753766
C	-0.224963	3.796325	-0.765168
C	-1.308402	4.199879	-0.003078
C	-1.971976	3.253772	0.759887
C	-1.561776	1.927843	0.750348
C	1.529026	-0.343854	0.001109
C	2.047983	-1.399363	-0.751227
C	3.402722	-1.701012	-0.761229
C	4.292119	-0.963678	0.002187
C	3.802286	0.082946	0.765093
C	2.448507	0.388966	0.754113
C	-1.062958	-1.153080	0.000755
C	-2.239187	-1.071714	-0.747372
C	-3.178369	-2.093617	-0.757788
C	-2.982404	-3.235349	0.000820
C	-1.828429	-3.337736	0.759309
C	-0.886067	-2.318757	0.748874
B	-0.000575	-0.000528	0.000275
H	-1.630111	5.233669	-0.003787
H	-3.716137	-4.031506	0.000894
H	5.348559	-1.200580	0.002610

Table S8. Computed Cartesian coordinates (x, y, z) for the optimized structure of B[3].

E [a.u.] = −4009.33
G [a.u.] = −4009.40

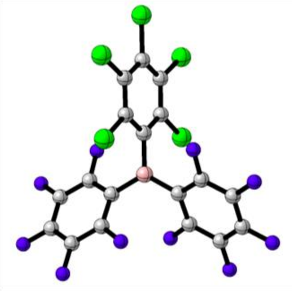

B	-0.000002	0.170743	0.000001
C	1.371856	-0.578786	0.013581
C	2.502923	-0.049561	0.644193
C	1.561174	-1.789951	-0.661993
C	3.737267	-0.683611	0.629771
C	2.783599	-2.440286	-0.711436
C	3.876177	-1.882900	-0.056655
F	2.425926	1.095100	1.321654
F	0.551861	-2.354472	-1.325516
F	4.780724	-0.154550	1.259465
F	2.922249	-3.581587	-1.377609
C	-1.371860	-0.578788	-0.013578
C	-1.561172	-1.789960	0.661985
C	-2.502932	-0.049560	-0.644179
C	-2.783595	-2.440298	0.711429
C	-3.737274	-0.683612	-0.629756
C	-3.876178	-1.882908	0.056659
F	-0.551855	-2.354487	1.325498
F	-2.425942	1.095106	-1.321631
F	-2.922239	-3.581606	1.377591
F	-4.780736	-0.154548	-1.259439
C	-0.000001	1.756234	-0.000001
C	-0.315105	2.472354	1.149398
C	0.315104	2.472348	-1.149404
C	-0.318856	3.867995	1.171756
C	0.318860	3.867989	-1.171767
C	0.000004	4.567097	-0.000007
Cl	0.675065	1.566427	-2.591245
Cl	-0.675069	1.566440	2.591243
Cl	-0.707649	4.719571	2.620908
Cl	0.000008	6.292807	-0.000010
Cl	0.707658	4.719558	-2.620923
F	-5.048678	-2.495815	0.088469
F	5.048679	-2.495804	-0.088464

Table S9. Computed Cartesian coordinates (x, y, z) for the optimized structure of B[4].

E [a.u.] = −1630.85
G [a.u.] = −1630.92

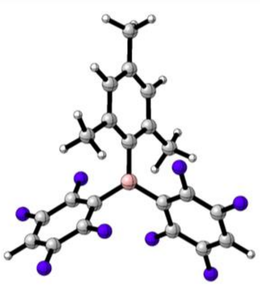

B	0.153088	-0.004479	-0.000519
C	-0.630623	1.359792	0.063565
C	-0.363871	2.392555	-0.829923
C	-1.618427	1.590471	1.016365
C	-1.052973	3.597868	-0.778670
C	-2.298774	2.797935	1.090099
C	-2.023386	3.811956	0.186550
F	0.551349	2.220807	-1.789665
F	-1.894538	0.641980	1.922537
F	-0.771412	4.552824	-1.666991
F	-3.222173	2.976595	2.036859
C	-0.699447	-1.326875	-0.065517
C	-1.694897	-1.508172	-1.020977
C	-0.489033	-2.370080	0.830865
C	-2.436344	-2.679145	-1.094625
C	-1.239208	-3.538379	0.779690
C	-2.216369	-3.704136	-0.188231
F	-1.918850	-0.548388	-1.929653
F	0.430822	-2.243687	1.793227
F	-3.364819	-2.811892	-2.043981
F	-1.010136	-4.504719	1.670738
C	1.709154	-0.044193	0.001046
C	2.407361	-0.656524	-1.058614
C	2.435491	0.528375	1.064115
C	3.799511	-0.671752	-1.046190
C	3.826385	0.467580	1.057101
C	4.527498	-0.119145	0.005478
H	-2.556849	4.752838	0.233416
H	-2.797444	-4.616402	-0.234972
H	4.378850	0.890794	1.893054
H	4.331277	-1.131070	-1.876504
C	1.673503	-1.277302	-2.225641
H	2.361377	-1.499167	-3.044908
H	1.190507	-2.216119	-1.934989
H	0.896946	-0.614829	-2.620209
C	1.732028	1.181262	2.232446
H	2.427623	1.358093	3.056166
H	1.304947	2.148306	1.946842
H	0.917370	0.561474	2.619279
C	6.033941	-0.130276	-0.007371
H	6.421629	-0.986523	-0.565601
H	6.423527	0.776818	-0.482628
H	6.440660	-0.170541	1.006581

Table S10. Computed Cartesian coordinates (x, y, z) for the optimized structure of B[5].

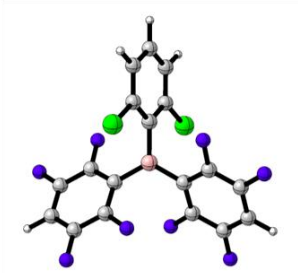

E [a.u.] = −2432.19
G [a.u.] = −2432.24

B	0	0.178890	0.000001
C	1.370156	-0.581585	-0.006593
C	2.494702	-0.079520	0.653739
C	1.550753	-1.773427	-0.714661
C	3.720014	-0.733663	0.622101
C	2.775554	-2.422412	-0.765457
C	3.871894	-1.909106	-0.092004
F	2.414173	1.044586	1.367870
F	0.536037	-2.309223	-1.398316
F	4.754565	-0.214326	1.285161
F	2.890995	-3.549966	-1.469028
C	-1.370156	-0.581587	0.006594
C	-1.550750	-1.773429	0.714662
C	-2.494702	-0.079524	-0.653738
C	-2.775551	-2.422416	0.765457
C	-3.720014	-0.733669	-0.622100
C	-3.871891	-1.909113	0.092005
F	-0.536034	-2.309224	1.398318
F	-2.414175	1.044582	-1.367869
F	-2.890990	-3.549971	1.469028
F	-4.754565	-0.214334	-1.285161
C	-0.000001	1.758231	-0.000001
C	-0.362940	2.496952	1.125976
C	0.362937	2.496951	-1.125978
C	-0.366253	3.884745	1.150006
C	0.366248	3.884744	-1.150011
C	-0.000003	4.574334	-0.000003
H	4.827444	-2.417202	-0.124008
H	-4.827441	-2.417210	0.124008
H	-0.000003	5.658799	-0.000004
H	0.647944	4.411851	-2.053654
H	-0.647950	4.411853	2.053649
Cl	0.797246	1.629123	-2.587121
Cl	-0.797248	1.629126	2.587119

Table S11. Computed Cartesian coordinates (x, y, z) for the optimized structure of B^{6}.

E [a.u.] = −2035.36
G [a.u.] = −2035.41

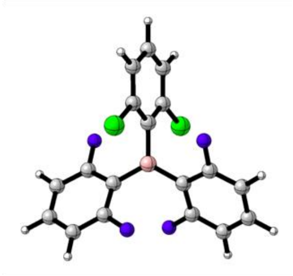

B	0.000001	0.167565	-0.000001
C	1.371316	-0.582957	-0.000192
C	2.511949	-0.082313	0.638659
C	1.577017	-1.786433	-0.686851
C	3.748673	-0.707811	0.624684
C	2.791200	-2.447727	-0.750437
C	3.879123	-1.897308	-0.081271
F	2.411945	1.057188	1.335886
F	0.548981	-2.325933	-1.356685
C	-1.371313	-0.582958	0.000191
C	-1.577011	-1.786438	0.686844
C	-2.511950	-0.082308	-0.638649
C	-2.791193	-2.447731	0.750434
C	-3.748674	-0.707806	-0.624670
C	-3.879120	-1.897307	0.081278
F	-0.548970	-2.325942	1.356668
F	-2.411948	1.057198	-1.335867
C	-0.000001	1.751886	-0.000003
C	-0.348433	2.493155	1.128832
C	0.348428	2.493154	-1.128840
C	-0.352257	3.881255	1.153393
C	0.352248	3.881254	-1.153405
C	-0.000006	4.572048	-0.000007
H	4.839143	-2.401389	-0.111732
H	-4.839141	-2.401387	0.111742
H	-0.000007	5.656618	-0.000008
H	0.623963	4.407453	-2.060731
H	-0.623974	4.407456	2.060718
Cl	0.767870	1.630679	-2.598581
Cl	-0.767871	1.630683	2.598575
H	-4.578271	-0.260499	-1.157913
H	-2.868257	-3.368980	1.314365
H	2.868266	-3.368972	-1.314374
H	4.578267	-0.260509	1.157935

Table S12. Computed Cartesian coordinates (x, y, z) for the optimized structure of B[7].

E [a.u.] = −3873.70
G [a.u.] = −3873.76

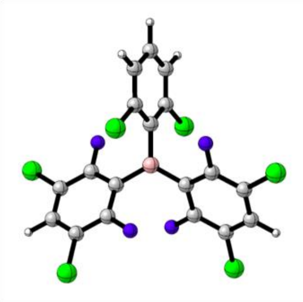

B	0.000003	0.443588	0.000005
C	-1.368719	-0.318980	0.061361
C	-2.518251	0.172844	-0.562421
C	-1.522770	-1.506038	0.783598
C	-3.747241	-0.476376	-0.487887
C	-2.739708	-2.168007	0.890174
C	-3.855683	-1.649406	0.245493
F	-2.440738	1.289412	-1.281165
F	-0.470290	-2.013513	1.423026
C	1.368715	-0.318998	-0.061357
C	1.522748	-1.506058	-0.783596
C	2.518256	0.172810	0.562421
C	2.739677	-2.168042	-0.890178
C	3.747237	-0.476426	0.487881
C	3.855661	-1.649455	-0.245501
F	0.470259	-2.013519	-1.423020
F	2.440760	1.289378	1.281167
C	0.000014	2.022216	0.000008
C	0.328280	2.760631	-1.136982
C	-0.328243	2.760631	1.137001
C	0.330858	4.148399	-1.160819
C	-0.330803	4.148399	1.160843
C	0.000032	4.837801	0.000014
H	-4.808482	-2.159866	0.315451
H	4.808454	-2.159928	-0.315464
H	0.000039	5.922239	0.000016
H	-0.584582	4.675662	2.072615
H	0.584644	4.675663	-2.072588
Cl	-0.715681	1.892842	2.610988
Cl	0.715706	1.892843	-2.610973
Cl	-2.860039	-3.627314	1.817895
Cl	-5.128553	0.180817	-1.303515
Cl	2.859985	-3.627350	-1.817901
Cl	5.128562	0.180749	1.303503

Table S13. Computed Cartesian coordinates (x, y, z) for the optimized structure of B[8].

E [a.u.] = −2954.53
G [a.u.] = −2954.59

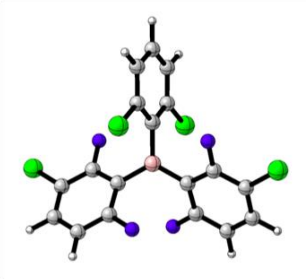

B	0.000000	-0.088936	0.000006
C	1.349334	-0.847877	-0.234218
C	2.574766	-0.346766	0.217719
C	1.426205	-2.042419	-0.958549
C	3.786925	-0.988941	-0.019207
C	3.798146	-2.169920	-0.750355
F	2.591594	0.777956	0.930090
F	0.297595	-2.570204	-1.449026
C	-1.349338	-0.847873	0.234226
C	-1.426215	-2.042414	0.958558
C	-2.574767	-0.346758	-0.217714
C	-3.786929	-0.988929	0.019210
C	-3.798156	-2.169908	0.750358
F	-0.297607	-2.570203	1.449037
F	-2.591590	0.777964	-0.930085
C	0.000002	1.492376	0.000004
C	-0.168624	2.232180	1.170337
C	0.168629	2.232178	-1.170330
C	-0.168161	3.620033	1.194789
C	0.168169	3.620031	-1.194785
C	0.000005	4.310013	0.000002
H	4.742155	-2.667484	-0.939762
H	-4.742166	-2.667470	0.939762
H	0.000006	5.394490	0.000001
H	0.294664	4.146764	-2.133053
H	-0.294655	4.146768	2.133057
Cl	0.351699	1.366828	-2.685589
Cl	-0.351696	1.366834	2.685598
Cl	5.262873	-0.311216	0.595520
Cl	-5.262873	-0.311199	-0.595521
C	2.610144	-2.704321	-1.231798
H	2.593515	-3.619351	-1.810303
C	-2.610156	-2.704312	1.231804
H	-2.593531	-3.619343	1.810309

Table S14. Computed Cartesian coordinates (x, y, z) for the optimized structure of B^{9}.

E [a.u.] = −12319.90
G [a.u.] = −12319.96

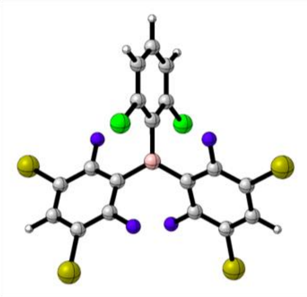

B	0.000002	0.838470	-0.000002
C	-1.365676	0.075677	0.101132
C	-2.534880	0.566781	-0.485764
C	-1.498044	-1.114124	0.823518
C	-3.759685	-0.082158	-0.375797
C	-2.708587	-1.778116	0.964544
C	-3.843245	-1.257606	0.356019
F	-2.476682	1.686364	-1.203014
F	-0.423601	-1.619778	1.428261
C	1.365677	0.075672	-0.101134
C	1.498042	-1.114129	-0.823521
C	2.534882	0.566769	0.485767
C	2.708582	-1.778128	-0.964543
C	3.759684	-0.082177	0.375804
C	3.843240	-1.257625	-0.356013
F	0.423598	-1.619777	-1.428269
F	2.476686	1.686350	1.203019
C	0.000005	2.417211	-0.000001
C	0.295321	3.155638	-1.145940
C	-0.295307	3.155638	1.145939
C	0.297300	4.543418	-1.169842
C	-0.297279	4.543417	1.169844
C	0.000012	5.232872	0.000002
H	-4.792854	-1.769372	0.453019
H	4.792846	-1.769395	-0.453009
H	0.000015	6.317320	0.000003
H	-0.524818	5.070647	2.088552
H	0.524842	5.070648	-2.088550
Cl	-0.639934	2.287982	2.630561
Cl	0.639944	2.287984	-2.630563
Br	-5.285527	0.631502	-1.211651
Br	-2.806822	-3.365737	1.967086
Br	2.806813	-3.365749	-1.967085
Br	5.285525	0.631474	1.211666

Table S15. Computed Cartesian coordinates (x, y, z) for the optimized structure of B^{10}.

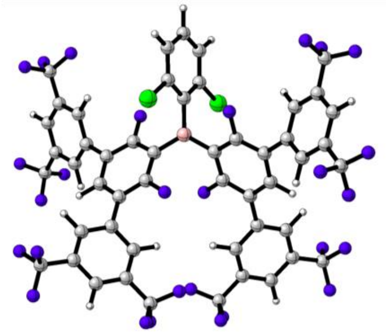

E [a.u.] = −5654.56
G [a.u.] = −5654.68

B	-0.002801	-1.827551	-0.016736	C	1.730273	3.136334	1.558665
C	-1.359369	-1.066844	-0.212669	C	3.604258	3.529700	3.563825
C	-2.578795	-1.579539	0.235228	H	4.288258	1.545461	3.113153
C	-1.442791	0.155113	-0.884315	C	1.747527	4.324561	2.275790
C	-3.810139	-0.950152	0.049793	H	1.010606	3.000295	0.761622
C	-2.626634	0.852624	-1.105159	C	2.684713	4.531324	3.282683
C	-3.799511	0.268515	-0.626788	H	2.694858	5.459019	3.841613
F	-2.567727	-2.729170	0.920922	C	5.071347	-1.520432	-0.592501
F	-0.311494	0.677172	-1.381217	C	5.997344	-0.694811	-1.230618
C	1.353678	-1.069145	0.188015	C	5.376083	-2.874253	-0.429878
C	1.437012	0.145267	0.873323	C	7.205410	-1.210069	-1.688434
C	2.573195	-1.577130	-0.265141	H	5.771201	0.355232	-1.383096
C	2.620867	0.840041	1.102510	C	6.583104	-3.381509	-0.893022
C	3.804364	-0.949619	-0.072614	H	4.669909	-3.533879	0.059356
C	3.793726	0.261428	0.617377	C	7.507059	-2.555415	-1.523826
F	0.305643	0.661868	1.375810	H	8.446943	-2.955889	-1.883680
F	2.562463	-2.719227	-0.963190	C	-0.769797	5.437787	-1.870384
C	-0.002989	-3.407660	-0.025974	C	-4.593718	3.764132	-4.654247
C	0.208703	-4.153870	1.133299	C	-8.175108	-0.315288	2.374612
C	-0.215003	-4.140126	-1.193910	C	-6.873060	-4.863883	0.700674
C	0.208646	-5.541826	1.149577	C	0.766120	5.415520	1.931048
C	-0.215759	-5.527791	-1.226537	C	4.591714	3.703045	4.688521
C	-0.003762	-6.224633	-0.042521	C	8.159072	-0.310140	-2.430506
H	-4.741406	0.784733	-0.788544	C	6.924109	-4.828762	-0.648084
H	4.735734	0.775665	0.784763	F	-6.423608	-5.554769	1.761302
H	-0.004050	-7.308999	-0.048868	F	-6.278893	-5.377958	-0.387757
H	-0.376116	-6.049524	-2.162440	F	-8.189293	-5.104614	0.598799
H	0.368679	-6.074615	2.079287	F	-9.406325	-0.839887	2.449860
Cl	-0.451693	-3.264155	-2.695804	F	-8.274741	0.873762	1.756651
Cl	0.446001	-3.295797	2.645335	F	-7.763131	-0.071597	3.629526
C	-5.076207	-1.526555	0.565636	F	-4.148286	3.207171	-5.792530
C	-6.005724	-0.707100	1.204828	F	-5.774029	3.189157	-4.370035
C	-5.379108	-2.880372	0.393233	F	-4.821476	5.061257	-4.905778
C	-7.216261	-1.226707	1.653651	F	-0.470304	6.202298	-2.931932
H	-5.782437	0.343089	1.362724	F	-1.258554	6.248859	-0.921845
C	-6.586721	-3.391776	0.848323	F	0.381181	4.920338	-1.407143
H	-4.675505	-3.533489	-0.108243	F	-0.385945	4.904727	1.463223
C	-7.515255	-2.570574	1.480510	F	1.253662	6.238434	0.992097
H	-8.460511	-2.971863	1.824138	F	0.468967	6.166539	3.002847
C	-2.646475	2.141563	-1.838697	F	4.824740	4.996731	4.952778
C	-3.584452	2.364750	-2.845643	F	5.769674	3.126123	4.398640
C	-1.735171	3.154124	-1.529973	F	4.143964	3.136856	5.821308
C	-3.607079	3.575460	-3.531309	F	7.850995	-0.244804	-3.736096
H	-4.292470	1.585537	-3.108106	F	8.123238	0.944651	-1.952821
C	-1.751235	4.351956	-2.230840	F	9.424602	-0.745045	-2.340341
H	-1.016213	3.006786	-0.734257	F	7.735484	-5.314037	-1.600263
C	-2.687402	4.572755	-3.235760	F	7.553511	-4.986898	0.528598
H	-2.696647	5.507914	-3.782113	F	5.825338	-5.598496	-0.616146
C	2.641408	2.119331	1.852738				
C	3.580390	2.328468	2.861853				

Table S16. Computed Cartesian coordinates (x, y, z) for the optimized structure of OC–B^{1}.

E [a.u.] = −2320.74
G [a.u.] = −2320.80

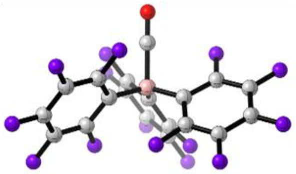

F	-1.940766	-1.712763	1.888726
F	-4.539692	-1.974999	1.237411
F	-5.588633	-0.484397	-0.790338
F	-4.002762	1.254826	-2.146818
F	-1.429184	1.514270	-1.522674
F	-0.530893	2.524591	1.899992
F	0.550461	4.908106	1.268859
F	2.381078	5.078298	-0.744534
F	3.099405	2.841851	-2.108432
F	2.027239	0.481242	-1.504565
F	2.449174	-0.765093	1.895105
F	3.996076	-2.882101	1.285445
F	3.237616	-4.591481	-0.698610
F	0.926521	-4.144921	-2.054709
F	-0.605053	-2.047974	-1.472387
C	-1.584621	-0.071923	0.235457
C	-2.158800	0.662226	-0.797517
C	-3.497530	0.534472	-1.150374
C	-4.309765	-0.356384	-0.462055
C	-3.774908	-1.115744	0.570854
C	-2.435363	-0.957117	0.887465
C	0.721806	1.402664	0.249168
C	1.652354	1.536442	-0.776422
C	2.215848	2.760335	-1.118587
C	1.847661	3.906144	-0.426472
C	0.914872	3.819030	0.599025
C	0.377759	2.578860	0.905047
C	0.849003	-1.331482	0.260725
C	0.503835	-2.224229	-0.749023
C	1.294009	-3.319489	-1.079649
C	2.477858	-3.548385	-0.391571
C	2.864077	-2.677180	0.619086
C	2.046897	-1.597971	0.914097
B	-0.008765	0.000502	0.623538
C	-0.018013	0.001993	2.263008
O	-0.019989	-0.002475	3.389376

Table S17. Computed Cartesian coordinates (x, y, z) for the optimized structure of OC–B[2].

E [a.u.] = −2023.14
G [a.u.] = −2023.20

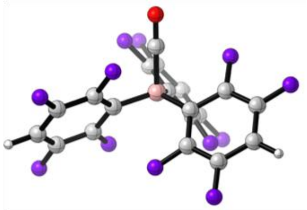

F	-1.936683	-1.695286	1.909755
F	-4.528737	-1.951900	1.269372
F	-3.984176	1.236192	-2.156222
F	-1.417178	1.488419	-1.544811
F	-0.537390	2.515376	1.901756
F	0.537724	4.892657	1.279358
F	3.093197	2.836461	-2.102558
F	2.026257	0.482955	-1.509499
F	2.438297	-0.774194	1.908744
F	3.974493	-2.888581	1.305186
F	0.926904	-4.121559	-2.069359
F	-0.592874	-2.027455	-1.494576
C	-1.582905	-0.076439	0.232039
C	-2.153366	0.649118	-0.808671
C	-3.495963	0.513883	-1.146482
C	-4.322158	-0.356354	-0.456988
C	-3.774425	-1.096167	0.577287
C	-2.434251	-0.949918	0.899076
C	0.719676	1.404534	0.244400
C	1.651038	1.539019	-0.780014
C	2.206328	2.771411	-1.108112
C	1.849418	3.920819	-0.424728
C	0.917494	3.813002	0.593541
C	0.373175	2.577172	0.905909
C	0.853304	-1.330884	0.254104
C	0.510552	-2.215654	-0.763063
C	1.308066	-3.310173	-1.081418
C	2.483105	-3.562279	-0.395124
C	2.848939	-2.690635	0.616595
C	2.043287	-1.604341	0.919247
B	-0.006534	-0.000528	0.619688
C	-0.013432	0.003025	2.257084
O	-0.013208	0.003643	3.383590
H	-5.366204	-0.459245	-0.723145
H	2.283304	4.878061	-0.683467
H	3.100993	-4.414632	-0.646488

Table S18. Computed Cartesian coordinates (x, y, z) for the optimized structure of OC–B3.

E [a.u.] = −4122.60
G [a.u.] = −4122.67

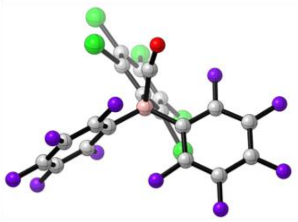

F	2.556811	1.049644	1.373906
F	0.399425	-2.297098	-1.178688
F	-1.106466	-2.069531	1.871824
F	-1.809483	0.757962	-1.845294
C	-0.087241	1.819928	0.087065
C	0.601637	2.405270	-0.986821
C	0.581076	3.784757	-1.227256
C	-0.179148	4.624139	-0.406990
C	-0.944257	4.069622	0.620357
C	-0.890893	2.689239	0.836838
C	1.375451	-0.563295	0.124103
C	1.477842	-1.722147	-0.642863
C	2.699319	-2.321410	-0.928793
C	3.873023	-1.771804	-0.430761
C	3.816331	-0.625495	0.351689
C	2.579662	-0.058401	0.611298
C	-1.380943	-0.579904	0.065221
C	-2.153693	-0.254060	-1.044465
C	-3.300719	-0.958839	-1.392725
C	-3.706236	-2.037906	-0.620544
C	-2.956938	-2.405284	0.490178
C	-1.820862	-1.675496	0.798052
B	-0.024164	0.218554	0.449312
C	0.119253	0.155440	2.088083
O	0.397490	0.044919	3.176063
Cl	-1.927639	2.039159	2.089480
Cl	1.446205	1.417838	-2.142220
F	-4.006935	-0.607975	-2.462920
F	-3.328255	-3.441187	1.236691
F	2.750868	-3.417922	-1.678283
F	4.930825	-0.091679	0.840262
Cl	-1.937463	5.086143	1.601305
Cl	-0.198103	6.327321	-0.679642
Cl	1.476801	4.465945	-2.537184
F	-4.799318	-2.717683	-0.942058
F	5.040796	-2.339992	-0.696952

Table S19. Computed Cartesian coordinates (x, y, z) for the optimized structure of OC–B^{4}.

E [a.u.] = −1744.13
G [a.u.] = −1744.19

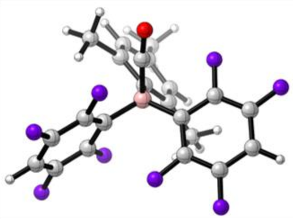

F	2.580665	0.909259	1.545486	C	0.123941	0.210964	2.090473
F	0.423507	-2.095997	-1.410305	O	0.318986	0.250686	3.202040
F	-1.185040	-1.958321	2.042814	H	-4.563362	-2.677822	-0.831942
F	-1.653808	0.541616	-1.942633	H	4.847476	-2.207027	-0.757358
C	-0.101188	1.823968	0.121143	H	1.123901	4.199822	-1.995140
C	0.630839	2.404080	-0.943555	H	-1.680435	4.661139	1.182803
C	0.557410	3.776409	-1.168485	F	-3.844680	-0.829368	-2.537952
C	-0.233801	4.621318	-0.394258	F	-3.383918	-3.345428	1.418660
C	-1.008455	4.037842	0.596508	F	2.751387	-3.208190	-1.960585
C	-0.963005	2.665681	0.856689	F	4.925634	-0.222334	0.961180
C	1.392811	-0.549373	0.115511	C	-1.902323	2.165526	1.937344
C	1.498286	-1.616092	-0.773997	H	-2.236947	1.137425	1.772777
C	2.721900	-2.199676	-1.087120	H	-1.459091	2.230223	2.937824
C	3.897521	-1.749201	-0.513574	H	-2.804040	2.782344	1.957901
C	3.821063	-0.694567	0.379944	C	1.440694	1.601273	-1.938434
C	2.594215	-0.120363	0.677368	H	2.413021	1.293158	-1.544270
C	-1.353174	-0.621519	0.101421	H	0.906757	0.703753	-2.256365
C	-2.067168	-0.382078	-1.069136	H	1.627955	2.202331	-2.831482
C	-3.205632	-1.108013	-1.399471	C	-0.256894	6.106528	-0.643658
C	-3.677389	-2.113091	-0.572260	H	-1.137708	6.573426	-0.195417
C	-2.975779	-2.381032	0.589966	H	0.628821	6.586928	-0.213679
C	-1.839736	-1.648287	0.901011	H	-0.261095	6.329198	-1.714584
B	-0.013069	0.225569	0.468698				

Table S20. Computed Cartesian coordinates (x, y, z) for the optimized structure of OC–B[5].

E [a.u.] = −2545.46
G [a.u.] = −2545.52

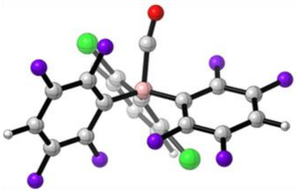

F	2.623355	0.988361	1.376749
F	0.384181	-2.282023	-1.212720
F	-1.277967	-1.896023	2.051598
F	-1.553971	0.572458	-1.966926
C	-0.061440	1.802832	0.123288
C	0.631857	2.447150	-0.916419
C	0.535087	3.811967	-1.167549
C	-0.293722	4.602603	-0.386964
C	-1.041291	4.019097	0.623253
C	-0.914990	2.653968	0.844484
C	1.405370	-0.585718	0.108882
C	1.479799	-1.727435	-0.685878
C	2.697096	-2.327110	-0.994515
C	3.889303	-1.820640	-0.508847
C	3.840486	-0.695248	0.296292
C	2.621986	-0.106411	0.592037
C	-1.348478	-0.583811	0.089798
C	-2.024635	-0.329195	-1.098658
C	-3.187193	-1.008089	-1.444829
C	-3.719021	-1.979745	-0.614969
C	-3.055499	-2.260594	0.566972
C	-1.896271	-1.574356	0.893754
B	0.017325	0.202789	0.470575
C	0.182030	0.151382	2.103736
O	0.484823	0.088622	3.189135
Cl	-1.950266	2.000591	2.117576
Cl	1.630274	1.563966	-2.055116
H	-4.623210	-2.509175	-0.885948
H	4.832904	-2.292523	-0.750753
H	1.101748	4.240946	-1.985072
H	-0.371209	5.666982	-0.578659
H	-1.719272	4.605123	1.231673
F	-3.789340	-0.715176	-2.599541
F	-3.523438	-3.194445	1.398651
F	2.702931	-3.410899	-1.772984
F	4.963044	-0.171300	0.792011

Table S21. Computed Cartesian coordinates (x, y, z) for the optimized structure of OC–B[6].

E [a.u.] = −2148.63
G [a.u.] = −2148.69

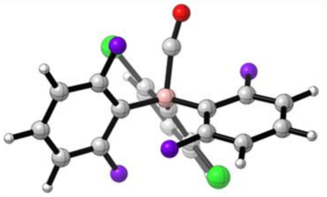

F	2.618388	0.997744	1.343901
F	0.402218	-2.298748	-1.178696
F	-1.312945	-1.862456	2.054548
F	-1.515739	0.535707	-1.976610
C	-0.063074	1.799138	0.126067
C	0.618253	2.449426	-0.918048
C	0.514747	3.814465	-1.166513
C	-0.308898	4.601896	-0.377351
C	-1.043125	4.013761	0.639669
C	-0.910273	2.648213	0.857054
C	1.412036	-0.587450	0.105686
C	1.511724	-1.741032	-0.673702
C	2.715506	-2.356345	-0.989860
C	3.897943	-1.815820	-0.501743
C	3.870907	-0.675742	0.293935
C	2.639474	-0.109921	0.567036
C	-1.348147	-0.583521	0.086503
C	-2.024627	-0.354544	-1.109324
C	-3.192577	-1.004786	-1.478592
C	-3.728729	-1.955499	-0.618782
C	-3.094437	-2.246823	0.583517
C	-1.931997	-1.559144	0.884428
B	0.020992	0.194153	0.464306
C	0.197321	0.131939	2.092755
O	0.514111	0.055475	3.174422
Cl	-1.929572	1.995047	2.143988
Cl	1.616326	1.574901	-2.064376
H	-4.640851	-2.476160	-0.888820
H	4.845617	-2.285922	-0.740666
H	1.073256	4.246152	-1.988323
H	-0.391671	5.666453	-0.566486
H	-1.715961	4.596296	1.257200
H	-3.654238	-0.764403	-2.428561
H	-3.476799	-2.987386	1.275106
H	2.704967	-3.242403	-1.612875
H	4.771138	-0.225412	0.693279

Table S22. Computed Cartesian coordinates (x, y, z) for the optimized structure of OC–B[7].

E [a.u.] = −3986.97
G [a.u.] = −3987.03

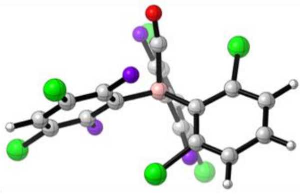

F	-2.721638	1.077459	-1.288382
F	-0.120050	-2.217686	0.827139
F	1.469796	-1.200548	-2.320800
F	1.438862	0.672327	1.979467
C	-0.155826	2.017252	0.059959
C	-0.913557	2.442036	1.165674
C	-0.966622	3.765203	1.591539
C	-0.231527	4.735866	0.929102
C	0.572416	4.373425	-0.139576
C	0.596267	3.042937	-0.536636
C	-1.344280	-0.505346	-0.245574
C	-1.285812	-1.745079	0.388595
C	-2.420774	-2.523208	0.613004
C	-3.664082	-2.077072	0.189471
C	-3.763804	-0.853835	-0.459383
C	-2.613143	-0.105356	-0.661525
C	1.396327	-0.189242	-0.206924
C	2.038606	-0.028716	1.017132
C	3.281983	-0.591818	1.292057
C	3.921523	-1.358726	0.328247
C	3.304398	-1.559459	-0.898432
C	2.063275	-0.981216	-1.131026
B	-0.056047	0.476529	-0.492107
C	-0.227222	0.625440	-2.119099
O	-0.533342	0.675587	-3.204088
Cl	1.690957	2.678177	-1.873907
Cl	-1.808312	1.315681	2.167706
Cl	4.021052	-0.340833	2.841320
Cl	4.065740	-2.521776	-2.124728
Cl	-2.273035	-4.048868	1.424989
Cl	-5.299852	-0.263703	-1.005174
H	4.888020	-1.801548	0.533589
H	-4.547949	-2.678587	0.361422
H	-1.575115	4.020383	2.450646
H	-0.270796	5.768471	1.257385
H	1.179840	5.103929	-0.659748

Table S23. Computed Cartesian coordinates (x, y, z) for the optimized structure of OC–B$^{8\text{-}I}$.

E [a.u.] = −3067.80
G [a.u.] = −3067.86

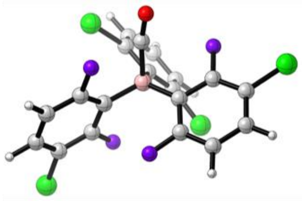

F	2.795136	0.697258	0.853891
F	0.049945	-2.538344	-1.176059
F	-0.966346	-2.080787	2.364075
F	-1.893225	0.304203	-1.568394
C	-0.058382	1.552234	0.220764
C	0.417084	2.205157	-0.930277
C	0.283972	3.573632	-1.142475
C	-0.362480	4.360778	-0.202483
C	-0.893490	3.770499	0.932934
C	-0.737415	2.401924	1.109841
C	1.338418	-0.858872	-0.119271
C	1.248984	-2.003719	-0.910759
C	2.349483	-2.632048	-1.476483
C	3.617930	-2.121263	-1.245220
C	2.638184	-0.396208	0.087792
C	-1.362830	-0.812243	0.429821
C	-2.244886	-0.564850	-0.618508
C	-3.478125	-1.201600	-0.734778
C	-3.860556	-2.137636	0.217277
C	-1.794845	-1.770489	1.336418
B	0.067077	-0.055268	0.526269
C	0.567280	-0.128545	2.087113
O	1.097722	-0.207111	3.080716
Cl	-1.492377	1.744389	2.565316
Cl	1.164227	1.331762	-2.254126
Cl	-4.519563	-0.826230	-2.074472
H	-4.819564	-2.633074	0.124688
H	4.492651	-2.594320	-1.675493
H	0.678541	4.008307	-2.052889
H	-0.467631	5.427855	-0.363650
H	-1.427108	4.353937	1.673276
C	3.770605	-0.991502	-0.450636
C	-3.007086	-2.433140	1.271630
H	-3.267573	-3.162965	2.027845
H	2.198651	-3.510061	-2.092293
Cl	5.342546	-0.324765	-0.134496

Table S24. Computed Cartesian coordinates (x, y, z) for the optimized structure of OC–B$^{8\text{-II}}$.

E [a.u.] = −3067.80
G [a.u.] = −3067.86

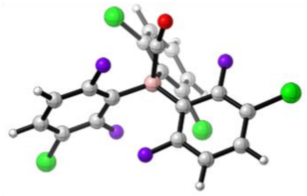

F	2.777359	0.759603	0.802307
F	-0.042116	-2.705287	-0.671008
F	-1.122094	-1.564162	2.695626
F	-1.864284	0.074845	-1.637376
C	-0.028338	1.575203	-0.052679
C	0.494749	1.989781	-1.290346
C	0.416113	3.299455	-1.753017
C	-0.220706	4.266347	-0.991021
C	-0.796442	3.912282	0.218417
C	-0.694203	2.595287	0.647041
C	1.284979	-0.901692	0.092796
C	1.169978	-2.169587	-0.476697
C	2.258126	-2.926661	-0.887463
C	3.539385	-2.422958	-0.720149
C	2.596282	-0.450822	0.246230
C	-1.423988	-0.666283	0.548363
C	-2.271201	-0.589461	-0.553708
C	-3.524977	-1.195072	-0.586902
C	-3.965293	-1.923680	0.510398
C	-1.913665	-1.423498	1.603666
B	0.030944	0.047935	0.544980
C	0.495067	0.246028	2.105966
O	1.001833	0.333634	3.111115
Cl	-1.502739	2.241324	2.177304
Cl	1.237503	0.864598	-2.411010
Cl	-4.519585	-1.041692	-2.003760
H	-4.940229	-2.395326	0.482576
H	4.404853	-2.995200	-1.032497
H	0.845264	3.546413	-2.716512
H	-0.283480	5.288386	-1.347643
H	-1.323686	4.638735	0.824661
C	3.716963	-1.171378	-0.143029
C	-3.148694	-2.045618	1.626480
H	-3.454678	-2.612341	2.496862
H	2.087949	-3.898040	-1.335063
Cl	5.305410	-0.508488	0.086983

Table S25. Computed Cartesian coordinates (x, y, z) for the optimized structure of OC–B$^{8\text{-III}}$.

E [a.u.] = −3067.80
G [a.u.] = −3067.86

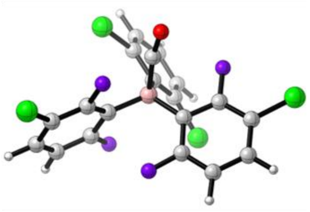

F	2.546408	-0.042914	1.377826
F	-0.364320	-2.209391	-1.615429
F	-2.141362	-1.448575	1.483384
F	-1.118871	1.168664	-2.275266
C	0.442500	1.701841	0.040805
C	1.417728	2.142231	-0.871214
C	1.810231	3.471665	-0.988149
C	1.215068	4.441446	-0.196429
C	0.212466	4.079132	0.688523
C	-0.149009	2.741012	0.777840
C	1.022693	-1.040065	-0.096972
C	0.807407	-2.101605	-0.975556
C	1.761656	-3.069234	-1.256525
C	2.998753	-3.006090	-0.632674
C	2.279557	-1.032902	0.508336
C	-1.554454	-0.089462	-0.334907
C	-1.981506	0.444117	-1.547923
C	-3.252038	0.255246	-2.069127
C	-4.163575	-0.516697	-1.362576
C	-2.498998	-0.870084	0.318411
B	-0.053579	0.149217	0.229020
C	-0.088285	-0.066248	1.855567
O	0.063926	-0.307199	2.947935
Cl	-1.471409	2.395043	1.896393
Cl	2.182310	1.052260	-2.012144
H	-5.163987	-0.682456	-1.744074
H	3.759841	-3.750325	-0.834982
H	2.572601	3.736545	-1.710744
H	1.519219	5.478642	-0.282722
H	-0.291047	4.817083	1.301068
C	3.265533	-1.979051	0.264519
C	-3.786353	-1.090800	-0.155392
H	1.524674	-3.857153	-1.960715
Cl	4.800760	-1.868594	1.068550
Cl	-4.894480	-2.074758	0.752463
H	-3.509819	0.705232	-3.019854

Table S26. Computed Cartesian coordinates (x, y, z) for the optimized structure of OC–B[9].

E [a.u.] = −12433.17
G [a.u.] = −12433.24

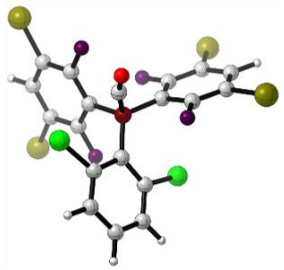

F	2.827250	1.566303	1.215262
F	0.168867	-1.613052	-0.996076
F	-1.089444	-1.089870	2.398026
F	-1.651994	1.289434	-1.600208
C	0.080978	2.491350	0.291962
C	0.685161	3.097150	-0.823864
C	0.621681	4.463259	-1.078145
C	-0.081841	5.295244	-0.221210
C	-0.739184	4.752640	0.871140
C	-0.649517	3.384714	1.093052
C	1.422600	0.032625	0.140177
C	1.354908	-1.124154	-0.634504
C	2.492155	-1.795231	-1.077439
C	3.751119	-1.322145	-0.739782
C	3.864913	-0.180972	0.041802
C	2.709998	0.460786	0.460432
C	-1.315535	0.172680	0.437965
C	-2.103282	0.437689	-0.678281
C	-3.338353	-0.166708	-0.887453
C	-3.820837	-1.084941	0.033764
C	-3.057317	-1.393840	1.150179
C	-1.830634	-0.768193	1.318555
B	0.123666	0.891520	0.648011
C	0.474094	0.850261	2.252072
O	0.901677	0.787694	3.294595
Cl	-1.564158	2.787668	2.480872
Cl	1.516041	2.163209	-2.052940
H	-4.780788	-1.561207	-0.120584
H	4.638185	-1.840546	-1.081588
H	1.114534	4.861166	-1.956905
H	-0.133074	6.360710	-0.415534
H	-1.320020	5.372536	1.543141
Br	-3.670097	-2.644025	2.415337
Br	-4.342821	0.255726	-2.421418
Br	2.315558	-3.340817	-2.135990
Br	5.554191	0.490347	0.522001

Table S27. Computed Cartesian coordinates (x, y, z) for the optimized structure of OC–B[10].

E [a.u.] = −5767.83
G [a.u.] = −5767.96

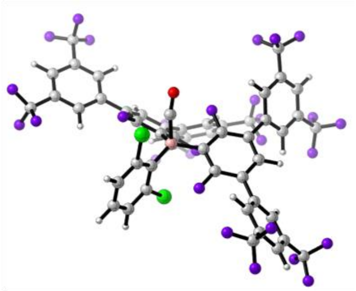

F	-3.015274	-2.606159	0.994386
F	-0.040297	0.456602	-0.930470
F	0.235871	0.472588	1.765116
F	2.383615	-3.278563	-0.057338
C	-0.336194	-3.572042	-0.078209
C	-0.237010	-3.682485	-1.475410
C	-0.366126	-4.879766	-2.167212
C	-0.592244	-6.053180	-1.462812
C	-0.660508	-6.017790	-0.079164
C	-0.524475	-4.797303	0.571699
C	-1.461616	-1.123551	0.076015
C	-1.283318	0.081975	-0.595919
C	-2.320584	0.940445	-0.956918
C	-3.622780	0.514595	-0.702294
C	-3.893873	-0.705100	-0.082850
C	-2.790384	-1.465058	0.301630
C	1.211510	-1.422265	0.792428
C	2.406507	-2.016845	0.392956
C	3.645399	-1.369292	0.408387
C	3.676960	-0.053438	0.864184
C	2.533820	0.601122	1.322174
C	1.352687	-0.130686	1.296914
B	-0.268115	-2.109590	0.626014
C	-0.703119	-2.130026	2.200897
O	-0.951065	-1.842153	3.265851
Cl	-0.562923	-4.874701	2.331286
Cl	0.130229	-2.271872	-2.447489
H	4.628596	0.468328	0.898597
H	-4.451579	1.154908	-0.989219
H	-0.279386	-4.884880	-3.247049
H	-0.698583	-6.994668	-1.989775
H	-0.811646	-6.922668	0.496693
C	-5.285507	-1.137437	0.192198
C	-5.698051	-2.447557	-0.063129
C	-6.216546	-0.224644	0.689279
C	-7.013396	-2.827279	0.171431
H	-4.991380	-3.172987	-0.447631
C	-7.532715	-0.611661	0.917360
H	-5.911373	0.792347	0.911489
C	-7.940809	-1.914226	0.661174
H	-8.965530	-2.214857	0.842510
C	-2.031903	2.263799	-1.560736
C	-2.760602	2.732787	-2.653693
C	-1.032009	3.075051	-1.020076
C	-2.491517	3.986544	-3.192618
H	-3.526717	2.108732	-3.101604
C	-0.758548	4.318631	-1.574540
H	-0.464249	2.733911	-0.161980
C	-1.485237	4.786122	-2.662527
H	-1.267991	5.755363	-3.093664
C	2.570765	2.020242	1.753836
C	1.941024	2.449142	2.921655
C	3.246701	2.959742	0.969078
C	1.987972	3.788984	3.294079
H	1.416154	1.738444	3.548256
C	3.295706	4.291861	1.352819
H	3.714717	2.652709	0.040920
C	2.664608	4.717471	2.516778
H	2.693637	5.760633	2.807944
C	4.885015	-2.035211	-0.063606
C	5.760568	-1.351470	-0.906308
C	5.213270	-3.334312	0.334213
C	6.943244	-1.947889	-1.333625
H	5.507889	-0.354478	-1.252387
C	6.392299	-3.924319	-0.100487
H	4.544117	-3.888203	0.980874
C	7.268093	-3.236462	-0.934868
H	8.182091	-3.704279	-1.278849
C	-7.455328	-4.225873	-0.173859
C	-8.501788	0.374768	1.515565
C	-3.333212	4.496672	-4.333182
C	0.381099	5.119436	-1.001691
C	1.239437	4.230642	4.524177
C	4.038082	5.307978	0.524844
C	6.756531	-5.302775	0.386873
C	7.878962	-1.157483	-2.210525
F	8.537733	-0.226800	-1.498823
F	7.211515	-0.509268	-3.179086
F	8.796938	-1.937196	-2.798880
F	7.517812	-5.958061	-0.502947
F	5.666812	-6.050496	0.620693
F	7.450731	-5.244317	1.536208
F	5.128516	5.751029	1.175468
F	4.447066	4.806622	-0.648171
F	3.274803	6.382757	0.269598
F	1.701838	5.395078	5.001671
F	-0.068166	4.395666	4.262229
F	1.330803	3.320661	5.508409
F	0.332291	5.146519	0.340491
F	1.563488	4.571397	-1.340036
F	0.384297	6.384862	-1.437190
F	-3.721210	3.499810	-5.144874
F	-4.449139	5.095216	-3.882649
F	-2.672606	5.395584	-5.077616
F	-9.768818	0.093703	1.176901
F	-8.436709	0.368619	2.857286
F	-8.239630	1.628729	1.112830
F	-7.877403	-4.301848	-1.447292
F	-6.455007	-5.109849	-0.034956
F	-8.469709	-4.631424	0.605266

Table S28. Computed Cartesian coordinates (x, y, z) for the optimized structure of TS-B[1].

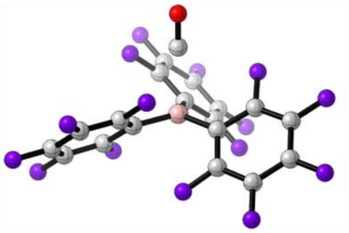

E [a.u.] = −2320.73
G [a.u.] = −2320.79

F	-1.738682	-1.912247	1.503777
F	-4.393932	-2.086695	1.195058
F	-5.690217	-0.362253	-0.462434
F	-4.289515	1.541790	-1.803239
F	-1.649515	1.733335	-1.508194
F	-0.783152	2.460104	1.507547
F	0.391472	4.847612	1.196160
F	2.529378	5.109190	-0.465689
F	3.475169	2.944774	-1.810063
F	2.322466	0.562339	-1.512882
F	2.520692	-0.553888	1.510833
F	4.000109	-2.765731	1.201234
F	3.160492	-4.745802	-0.464609
F	0.816459	-4.480406	-1.813885
F	-0.670235	-2.290435	-1.517948
C	-1.576274	-0.072755	0.027705
C	-2.284148	0.788136	-0.809300
C	-3.659672	0.702595	-0.987191
C	-4.377069	-0.271292	-0.306922
C	-3.713115	-1.152597	0.538419
C	-2.339286	-1.042342	0.677747
C	0.725442	1.400955	0.027373
C	1.822813	1.584098	-0.812194
C	2.435662	2.818379	-0.991282
C	1.952337	3.926216	-0.309396
C	0.859099	3.791211	0.538473
C	0.268587	2.546075	0.679084
C	0.851419	-1.329017	0.026923
C	0.462804	-2.369886	-0.814690
C	1.225221	-3.517894	-0.993248
C	2.424705	-3.654395	-0.308800
C	2.853060	-2.641160	0.540939
C	2.070306	-1.506827	0.680756
B	0.000129	-0.000397	0.197466
C	-0.001104	-0.000868	2.480761
O	-0.003610	-0.001008	3.610687

Table S29. Computed Cartesian coordinates (x, y, z) for the optimized structure of TS-B[5].

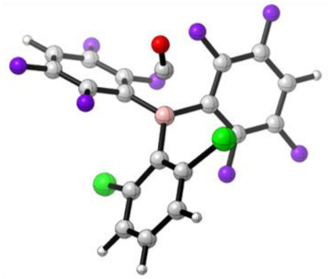

E [a.u.] = −2545.45
G [a.u.] = −2545.51

F	2.468591	1.001789	1.382010
F	0.486643	-2.264375	-1.415594
F	-0.711953	-2.253768	1.510890
F	-2.192811	0.899606	-1.694231
C	-0.038143	1.790684	-0.093352
C	0.745574	2.437212	-1.066738
C	0.779154	3.815600	-1.237295
C	-0.005861	4.621392	-0.427291
C	-0.844650	4.041716	0.511774
C	-0.862153	2.658834	0.645298
C	1.369520	-0.582182	0.008565
C	1.518524	-1.750486	-0.737896
C	2.738296	-2.407903	-0.845661
C	3.863362	-1.925964	-0.200632
C	3.742853	-0.768767	0.550350
C	2.523273	-0.115593	0.639733
C	-1.365072	-0.603585	-0.056627
C	-2.356199	-0.197999	-0.949253
C	-3.534494	-0.916202	-1.120173
C	-3.770377	-2.071351	-0.397143
C	-2.798384	-2.499239	0.492406
C	-1.621699	-1.782613	0.642013
B	-0.010588	0.205451	0.106521
C	0.119366	0.143122	2.453592
O	0.296157	0.003389	3.562548
Cl	-2.028164	2.041704	1.808718
Cl	1.687543	1.517975	-2.228917
H	-4.688569	-2.629803	-0.526907
H	4.812786	-2.439500	-0.281931
H	1.408898	4.243394	-2.007827
H	0.017313	5.699135	-0.544606
H	-1.493760	4.648587	1.131093
F	-4.442334	-0.476703	-1.993755
F	-2.985049	-3.611404	1.206348
F	2.815328	-3.516600	-1.584102
F	4.802621	-0.269794	1.189724

Table S30. Computed Cartesian coordinates (x, y, z) for the optimized structure of TS-B[7].

E [a.u.] = −3986.96
G [a.u.] = −3987.02

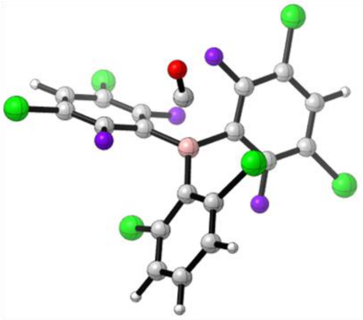

F	2.565960	1.187068	1.243730
F	0.319868	-2.018253	-1.360791
F	-0.678906	-1.937790	1.597952
F	-2.236505	1.215703	-1.519822
C	0.028109	2.046630	-0.086920
C	0.777224	2.647110	-1.115665
C	0.852743	4.020389	-1.311729
C	0.147478	4.868209	-0.471767
C	-0.656397	4.336017	0.524236
C	-0.718096	2.957147	0.682859
C	1.353115	-0.374236	-0.026346
C	1.418733	-1.556818	-0.763081
C	2.601044	-2.271056	-0.936545
C	3.774451	-1.809059	-0.358040
C	3.754996	-0.635339	0.383215
C	2.559408	0.057165	0.526095
C	-1.382729	-0.296494	0.070064
C	-2.415803	0.135057	-0.761472
C	-3.635518	-0.531494	-0.852650
C	-3.853351	-1.671277	-0.093152
C	-2.846955	-2.140910	0.741044
C	-1.638658	-1.459268	0.795507
B	0.008746	0.465102	0.140421
C	0.266134	0.437873	2.472677
O	0.497987	0.314593	3.573193
Cl	-1.837789	2.402498	1.920694
Cl	1.614083	1.674305	-2.314086
Cl	-4.871834	0.065185	-1.912293
Cl	-3.089102	-3.567979	1.696099
Cl	2.603074	-3.729181	-1.875281
Cl	5.205826	-0.032935	1.117633
H	-4.799318	-2.194987	-0.154112
H	4.698768	-2.358845	-0.485175
H	1.452000	4.411729	-2.124766
H	0.204404	5.942296	-0.609171
H	-1.245197	4.976963	1.168945

Table S31. Computed Cartesian coordinates (x, y, z) for the optimized structure of TS-B[9].

E [a.u.] = −12433.16
G [a.u.] = −12433.23

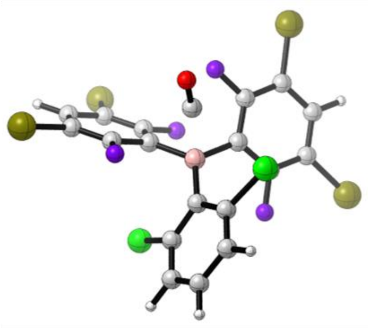

F	2.649250	1.562342	1.158157
F	0.245440	-1.632403	-1.308326
F	-0.609492	-1.523645	1.683768
F	-2.268680	1.636025	-1.368177
C	0.063432	2.443676	-0.044608
C	0.773456	3.034028	-1.106247
C	0.853575	4.406012	-1.309667
C	0.193143	5.262998	-0.442949
C	-0.571942	4.741307	0.588529
C	-0.639695	3.363518	0.754033
C	1.361546	0.006541	-0.039048
C	1.378922	-1.183334	-0.767073
C	2.541013	-1.914441	-0.988863
C	3.744978	-1.462057	-0.469813
C	3.776624	-0.281750	0.259658
C	2.598441	0.425232	0.452466
C	-1.364881	0.119014	0.185699
C	-2.430653	0.559736	-0.598375
C	-3.660725	-0.089850	-0.630498
C	-3.855836	-1.224245	0.142304
C	-2.818381	-1.705456	0.929795
C	-1.602515	-1.038213	0.926032
B	0.037316	0.862874	0.188164
C	0.404100	0.840245	2.501560
O	0.689091	0.716639	3.589492
Cl	-1.709606	2.823312	2.041553
Cl	1.549150	2.049980	-2.336086
H	-4.810188	-1.735768	0.127715
H	4.655124	-2.025162	-0.634666
H	1.421134	4.789269	-2.148879
H	0.254244	6.336093	-0.586230
H	-1.126289	5.389624	1.256056
Br	-3.051859	-3.247251	1.981130
Br	-5.046678	0.571415	-1.716816
Br	2.476180	-3.505726	-1.989823
Br	5.394261	0.357185	0.975083

Table S32. Computed Cartesian coordinates (x, y, z) for the optimized structure of TS-B[10].

E [a.u.] = −5767.82
G [a.u.] = −5767.95

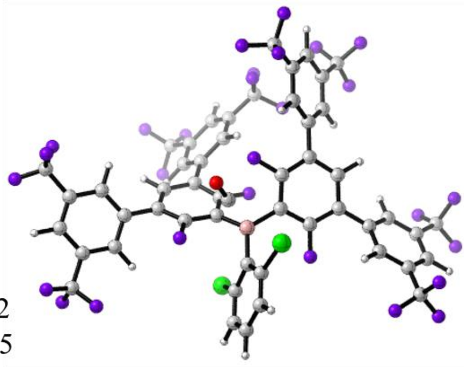

F	-3.037814	-2.613284	0.740986
F	-0.009473	0.370454	-1.231231
F	0.161738	0.471779	1.634924
F	2.324194	-3.100153	-0.504450
C	-0.313202	-3.660173	0.187602
C	-0.841287	-4.279377	-0.959487
C	-0.957240	-5.656101	-1.107988
C	-0.523446	-6.489885	-0.089078
C	0.056114	-5.943571	1.045848
C	0.167276	-4.563295	1.152627
C	-1.447997	-1.161019	-0.178536
C	-1.254920	0.041373	-0.853443
C	-2.269811	0.942219	-1.168637
C	-3.576315	0.550864	-0.887390
C	-3.867657	-0.672009	-0.281513
C	-2.783213	-1.472554	0.070529
C	1.163891	-1.371807	0.583719
C	2.359117	-1.910510	0.112612
C	3.599177	-1.274533	0.206749
C	3.622399	-0.007900	0.784472
C	2.469946	0.610906	1.271729
C	1.287822	-0.111476	1.167645
B	-0.244926	-2.071174	0.344166
C	-1.037465	-1.875875	2.496924
O	-1.507423	-1.623318	3.494608
Cl	0.996362	-3.994319	2.597209
Cl	-1.323973	-3.333914	-2.359279
H	4.573153	0.507790	0.881192
H	-4.392756	1.220506	-1.141519
H	-1.375128	-6.062182	-2.021128
H	-0.615454	-7.565609	-0.189068
H	0.433296	-6.576218	1.840013
C	-5.266216	-1.074127	0.004781
C	-5.717884	-2.362100	-0.293817
C	-6.162974	-0.156266	0.551583
C	-7.038729	-2.715475	-0.050649
H	-5.036617	-3.088936	-0.719754
C	-7.485404	-0.517036	0.788386
H	-5.826583	0.843479	0.805249
C	-7.932524	-1.797490	0.490477
H	-8.961805	-2.077732	0.678555
C	-1.948962	2.277532	-1.727337
C	-2.654181	2.803705	-2.809061
C	-0.943106	3.046413	-1.138720
C	-2.354050	4.073590	-3.291172
H	-3.426982	2.213527	-3.290198
C	-0.641786	4.308572	-1.633467
H	-0.398217	2.659208	-0.284835
C	-1.343550	4.833017	-2.711775
H	-1.105268	5.816654	-3.096705
C	2.502591	1.994904	1.805693
C	1.851973	2.341438	2.988943
C	3.197367	2.984589	1.103302
C	1.898582	3.650555	3.458615
H	1.312267	1.589872	3.552324
C	3.245329	4.284915	1.583022
H	3.682441	2.742461	0.164947
C	2.594515	4.628629	2.763436
H	2.626801	5.646685	3.133054
C	4.843063	-1.904384	-0.300010
C	5.745382	-1.157766	-1.057185
C	5.145866	-3.238208	-0.014007
C	6.929134	-1.729931	-1.512231
H	5.513191	-0.130005	-1.315887
C	6.326990	-3.803362	-0.476151
H	4.455494	-3.837379	0.567338
C	7.228405	-3.054956	-1.226160
H	8.143091	-3.503339	-1.593995
C	-7.525814	-4.086792	-0.441388
C	-8.416529	0.472492	1.439341
C	-3.169113	4.647909	-4.420674
C	0.488693	5.072404	-0.995103
C	1.133308	4.004660	4.706782
C	3.992625	5.358916	0.836522
C	6.662630	-5.225830	-0.109574
C	7.905559	-0.881949	-2.285008
F	8.672844	-0.151388	-1.457998
F	7.272982	-0.020455	-3.097920
F	8.727437	-1.626472	-3.038631
F	7.434939	-5.808318	-1.039560
F	5.558305	-5.975322	0.031811
F	7.331524	-5.282829	1.054578
F	4.989626	5.858663	1.587619
F	4.534288	4.904500	-0.301352
F	3.187930	6.388089	0.525591
F	1.586207	5.134430	5.269193
F	-0.171437	4.184046	4.440521
F	1.215809	3.029022	5.626537
F	0.379761	5.071379	0.343723
F	1.674438	4.506895	-1.291573
F	0.536368	6.345933	-1.402392
F	-3.572926	3.692982	-5.273803
F	-4.274214	5.258073	-3.959411
F	-2.476707	5.556727	-5.122951
F	-9.698276	0.226661	1.130183
F	-8.313415	0.430278	2.777976
F	-8.139086	1.730988	1.061317
F	-7.984184	-4.098222	-1.704367
F	-6.545782	-5.000726	-0.367316
F	-8.529223	-4.499306	0.348415

19-3. Discussion on the effects of *meta*-substituents on B^n (n = 5, 7, 9-10)

In the $\mathbf{B^n}$-catalyzed hydrogenation of **Qin** in the co-presence of CO and CO_2 under the applied neat conditions (n= 1, 5, 7, 9-10; Fig S18), the higher catalyst turnover number (TON) was confirmed in the following order; $\mathbf{B^9}$ (1480) > $\mathbf{B^7}$ (1400) > $\mathbf{B^{10}}$ (1340) > $\mathbf{B^5}$ (1000) >> $\mathbf{B^1}$ (11). These results demonstrated the importance of the introduction of four F and two Cl atoms at the *ortho*-positions with respect to the boron center. Then, to clarify an impact of *meta*-substituents, we analyzed these results based on the parameters obtained by the Gutmann-Beckett method and the energy levels of their LUMO (Fig. S44a). It should be noted that the Gutmann-Beckett acceptor numbers (GB-ANs) should be influenced by both an electron-accepting ability on the boron atom, i.e. the energy level of LUMO, and steric repulsions that occurred between $Et_3P{=}O$ (i.e. a front strain) and/or between other Ar groups (i.e. a back strain) (Fig. S44b).[43] For $\mathbf{B^5}$, $\mathbf{B^7}$, and $\mathbf{B^9}$–$\mathbf{B^{10}}$, TON shows a good correlation with GB-ANs. In contrast, a profile of TON with respect to the energy levels of LUMO shows a rather large variation. Based on these results and the size of substituents such as F, Cl, Br,[56] and $(CF_3)_2C_6H_3$, the *meta*-substituents play a key role to establish the suitable Lewis acidity not only by regulating of the electron-accepting ability on the boron atom but also by inducing the proper front/back strains. In this context, the $(CF_3)_2C_6H_3$ groups in $\mathbf{B^{10}}$ can reduce their steric demanding via the rotation of C_{Ar}–C_{Ar} bond, which was indeed observed in **OC–$\mathbf{B^{10}}$**.

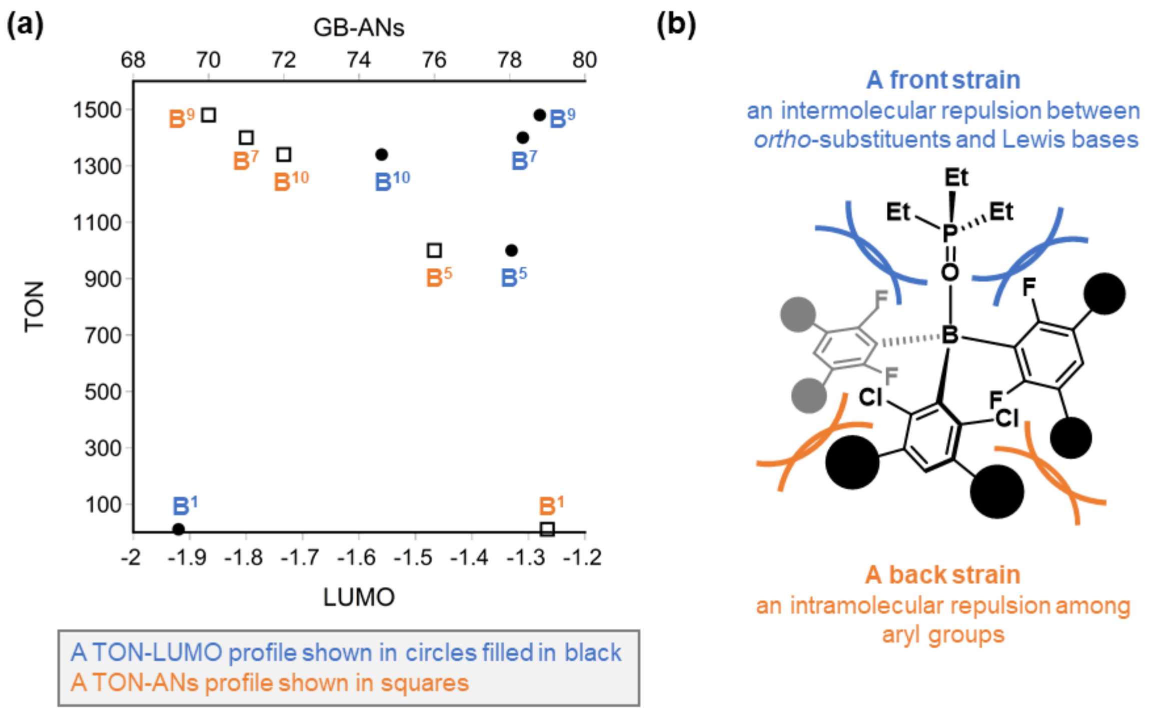

Fig S44. Discussion on the effects of *meta*-substituents on B^n (n = 5, 7, 9-10). (a) Profiles of TON vs the LUMO energies in eV (shown in black-filled circles) and vs Gutmann-Beckett acceptor numbers (GB-ANs; shown in squares). (b) A schematic representation for a concept of front and back strains generated via the complexation of $Et_3P{=}O{-}B^n$ (n = 5, 7, 9-10).

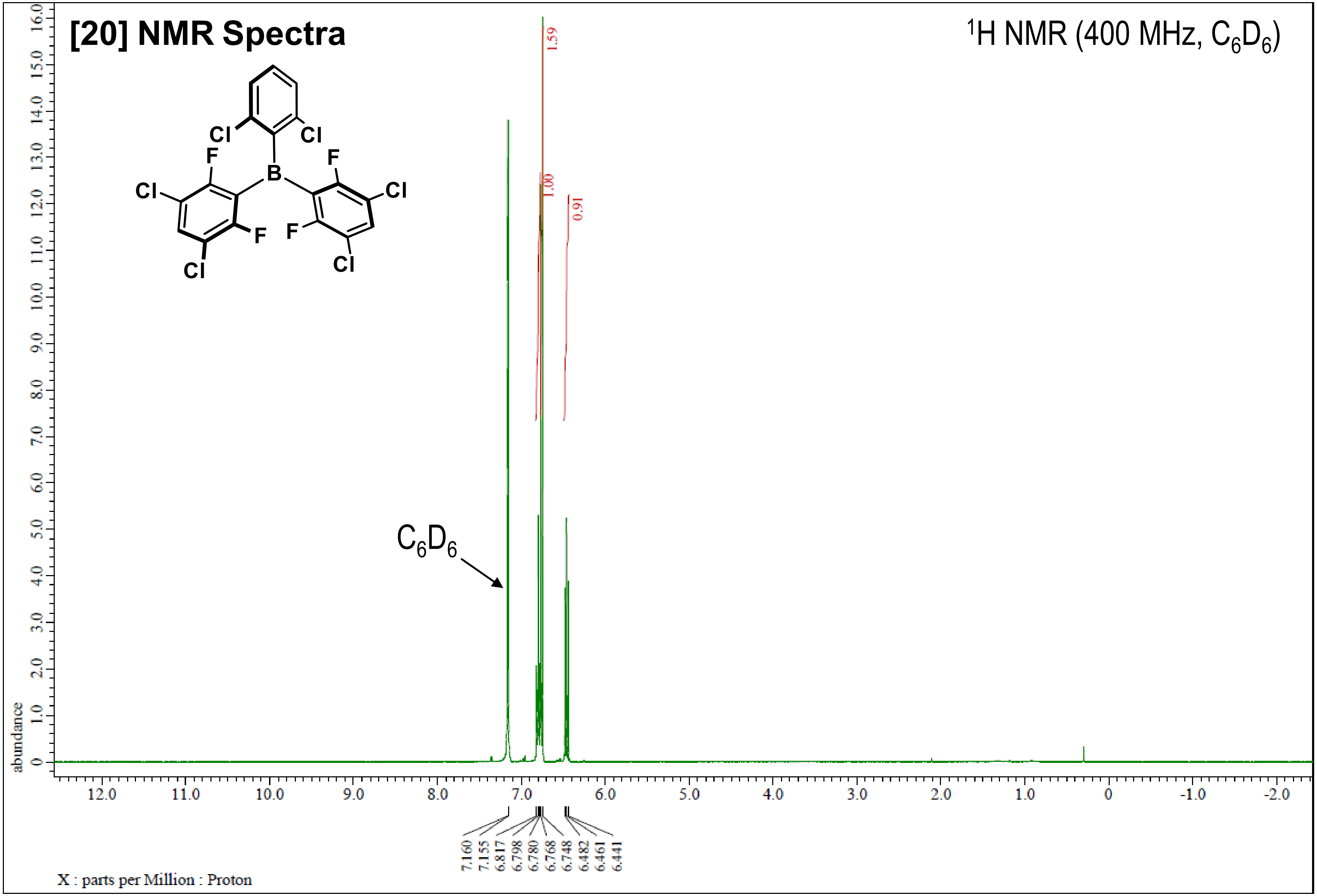
[20] NMR Spectra
^{1}H NMR (400 MHz, C_6D_6)
Cl
Cl
F
B
F
Cl
Cl
F
F
Cl
Cl
C_6D_6
1.59
1.00
0.91
7.160
7.155
6.817
6.798
6.780
6.768
6.748
6.482
6.461
6.441
abundance
X : parts per Million : Proton

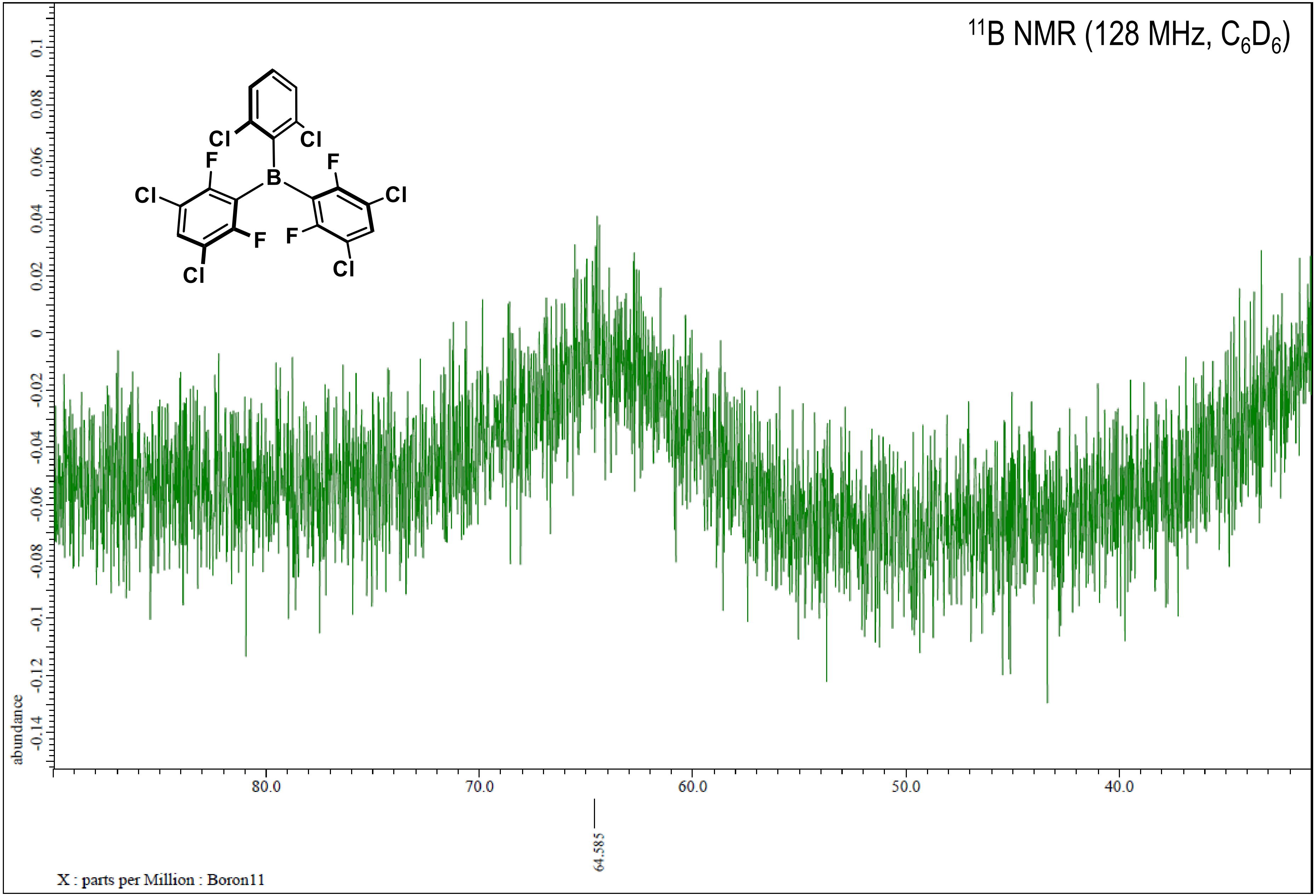

^{11}B NMR (128 MHz, C_6D_6)
Cl
Cl
F
B
F
Cl
Cl
F
F
Cl
Cl
0.1
0.08
0.06
0.04
0.02
0
-0.02
-0.04
-0.06
-0.08
-0.1
-0.12
-0.14
abundance
80.0
70.0
60.0
50.0
40.0
64.585
X : parts per Million : Boron11

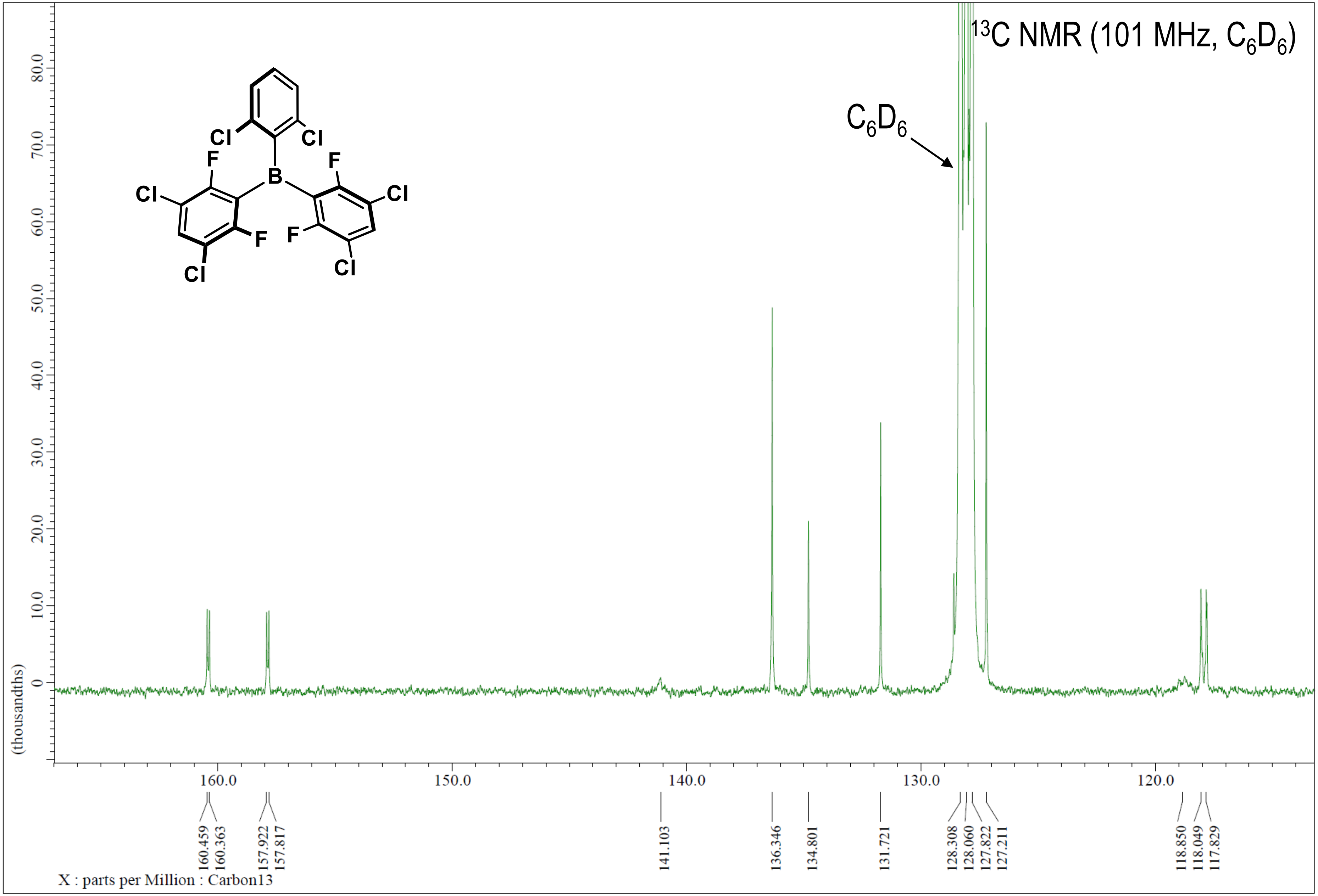

13C NMR (101 MHz, C6D6)
C6D6
Cl
Cl
F
B
Cl
F
Cl
F
Cl
F
Cl
Cl
(thousandths)
80.0
70.0
60.0
50.0
40.0
30.0
20.0
10.0
0
160.0
150.0
140.0
130.0
120.0
160.459
160.363
157.922
157.817
141.103
136.346
134.801
131.721
128.308
128.060
127.822
127.211
118.850
118.049
117.829
X : parts per Million : Carbon13

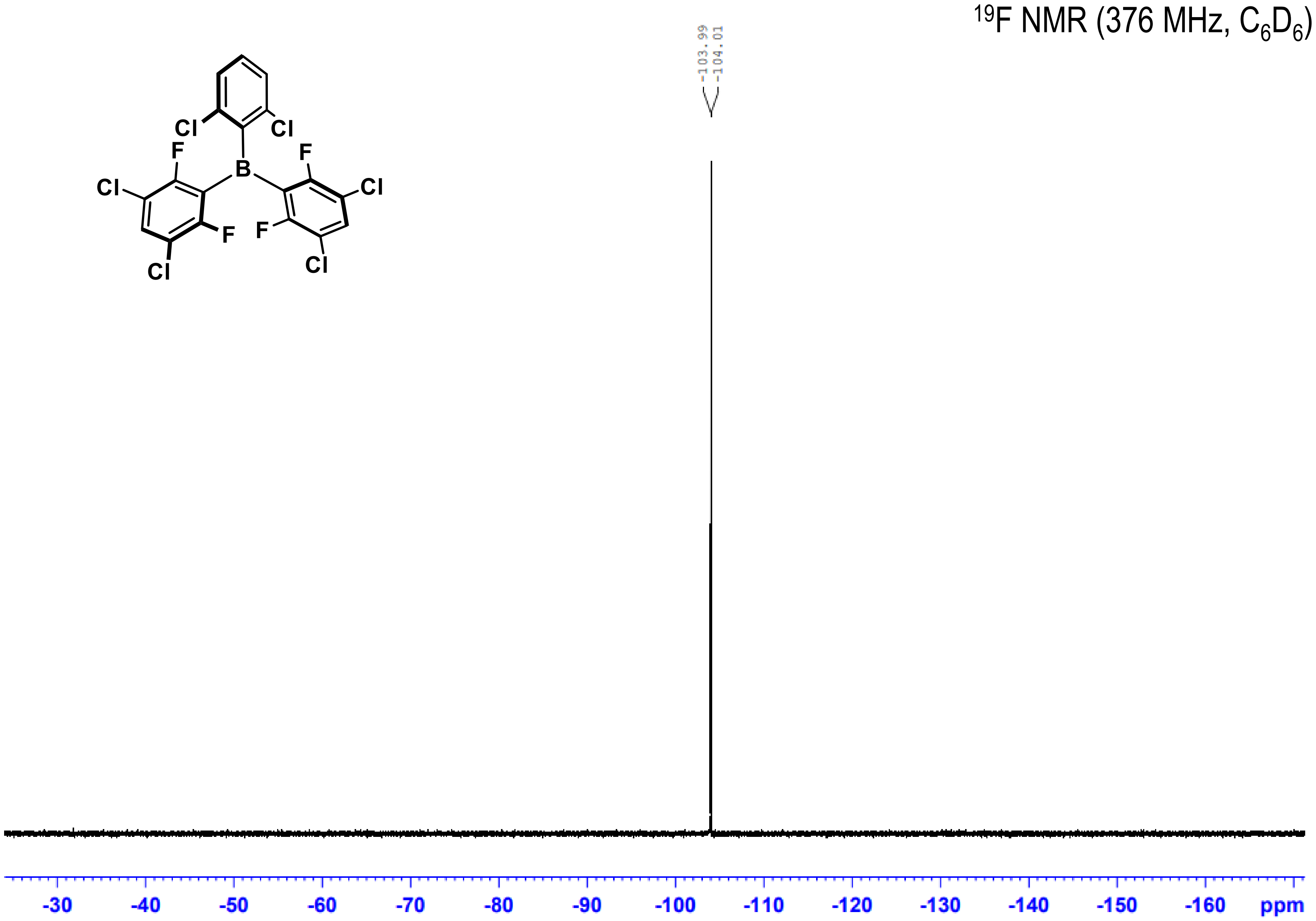

19F NMR (376 MHz, C6D6)
-103.99
-104.01
Cl
Cl
F
F
B
Cl
Cl
F
F
Cl
Cl
-30
-40
-50
-60
-70
-80
-90
-100
-110
-120
-130
-140
-150
-160
ppm

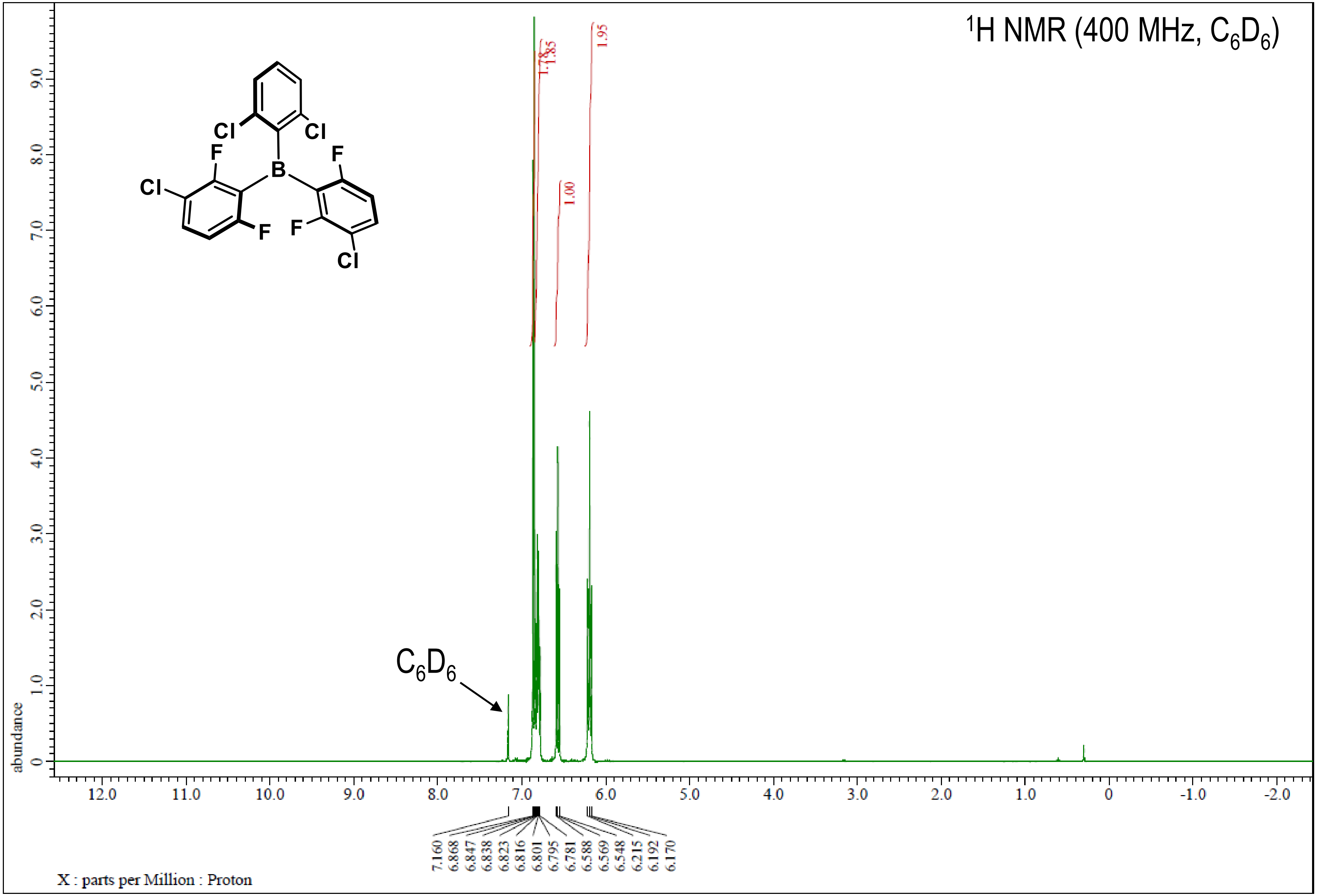

^{1}H NMR (400 MHz, C_6D_6)
C_6D_6
1.78
1.85
1.00
1.95
7.160
6.868
6.847
6.838
6.823
6.816
6.801
6.795
6.781
6.588
6.569
6.548
6.215
6.192
6.170
abundance
X : parts per Million : Proton

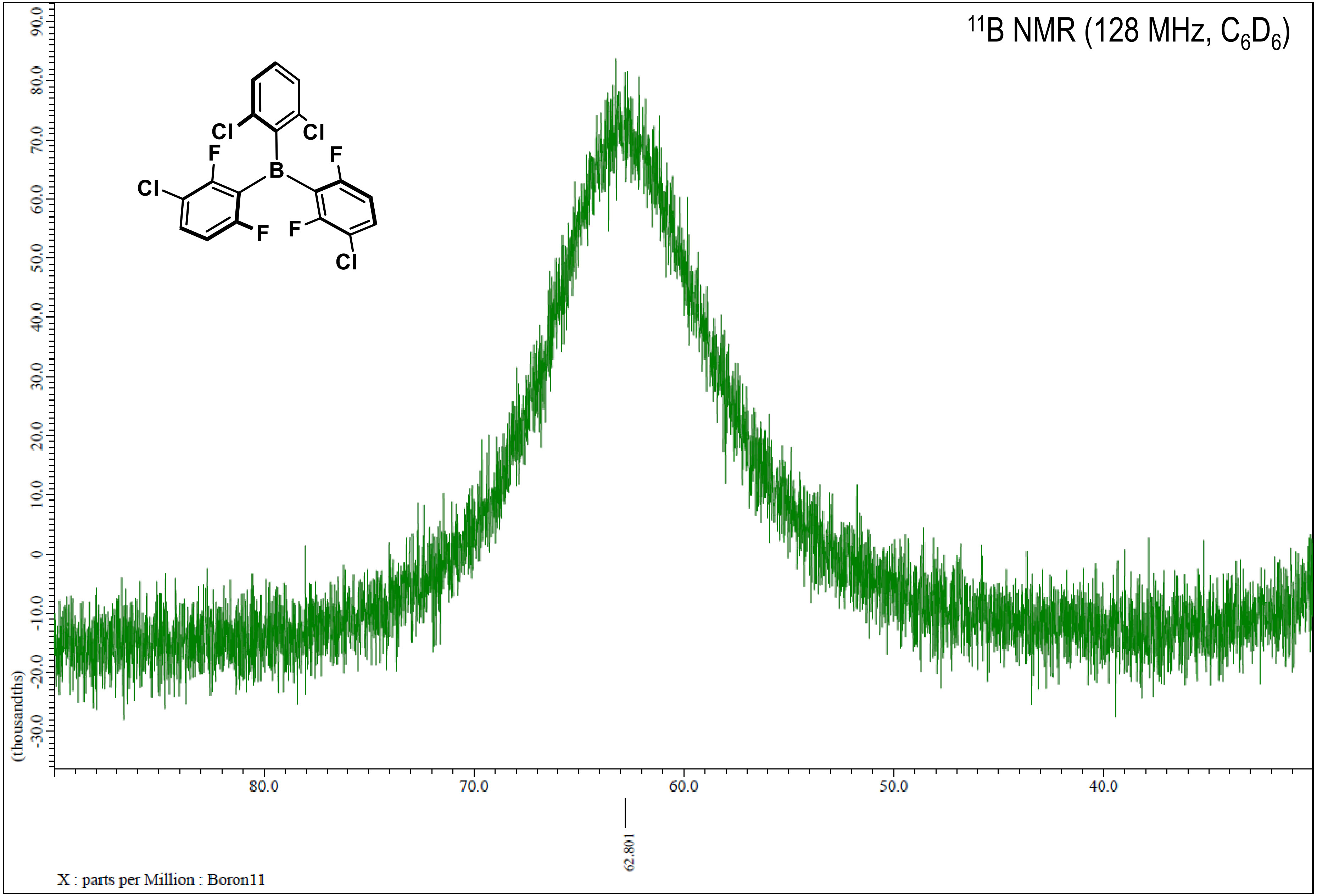

11B NMR (128 MHz, C6D6)
Cl
Cl
F
F
B
Cl
F
F
Cl
90.0
80.0
70.0
60.0
50.0
40.0
30.0
20.0
10.0
0
-10.0
-20.0
-30.0
(thousandths)
80.0
70.0
60.0
50.0
40.0
62.801
X : parts per Million : Boron11

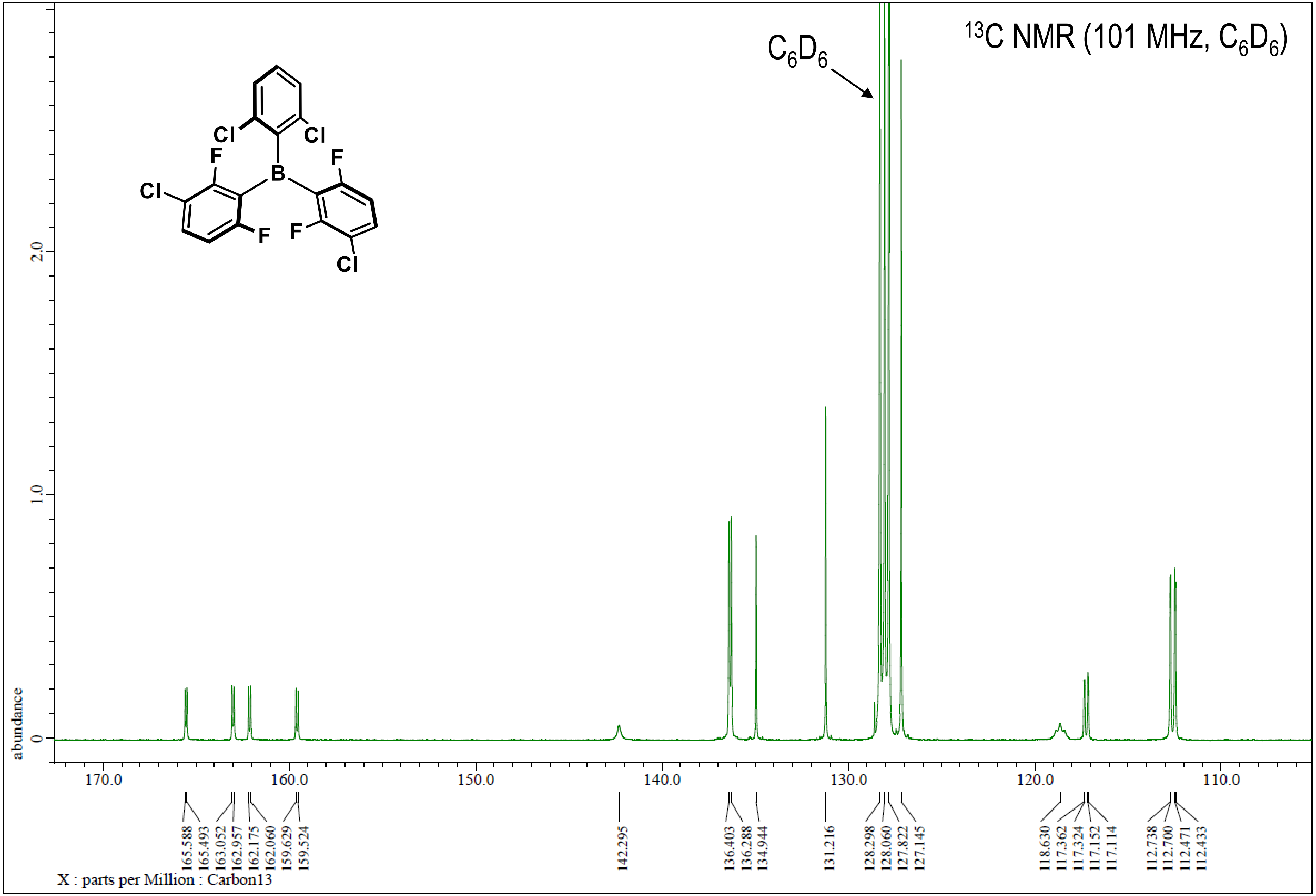

13C NMR (101 MHz, C6D6)
C6D6
Cl
Cl
F
Cl
B
F
F
F
Cl
Cl
abundance
2.0
1.0
0
170.0
160.0
150.0
140.0
130.0
120.0
110.0
165.588
165.493
163.052
162.957
162.175
162.060
159.629
159.524
142.295
136.403
136.288
134.944
131.216
128.298
128.060
127.822
127.145
118.630
117.362
117.324
117.152
117.114
112.738
112.700
112.471
112.433
X : parts per Million : Carbon13

^{19}F NMR (376 MHz, C_6D_6)

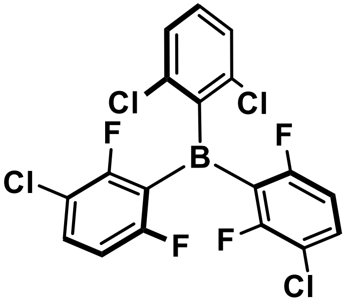

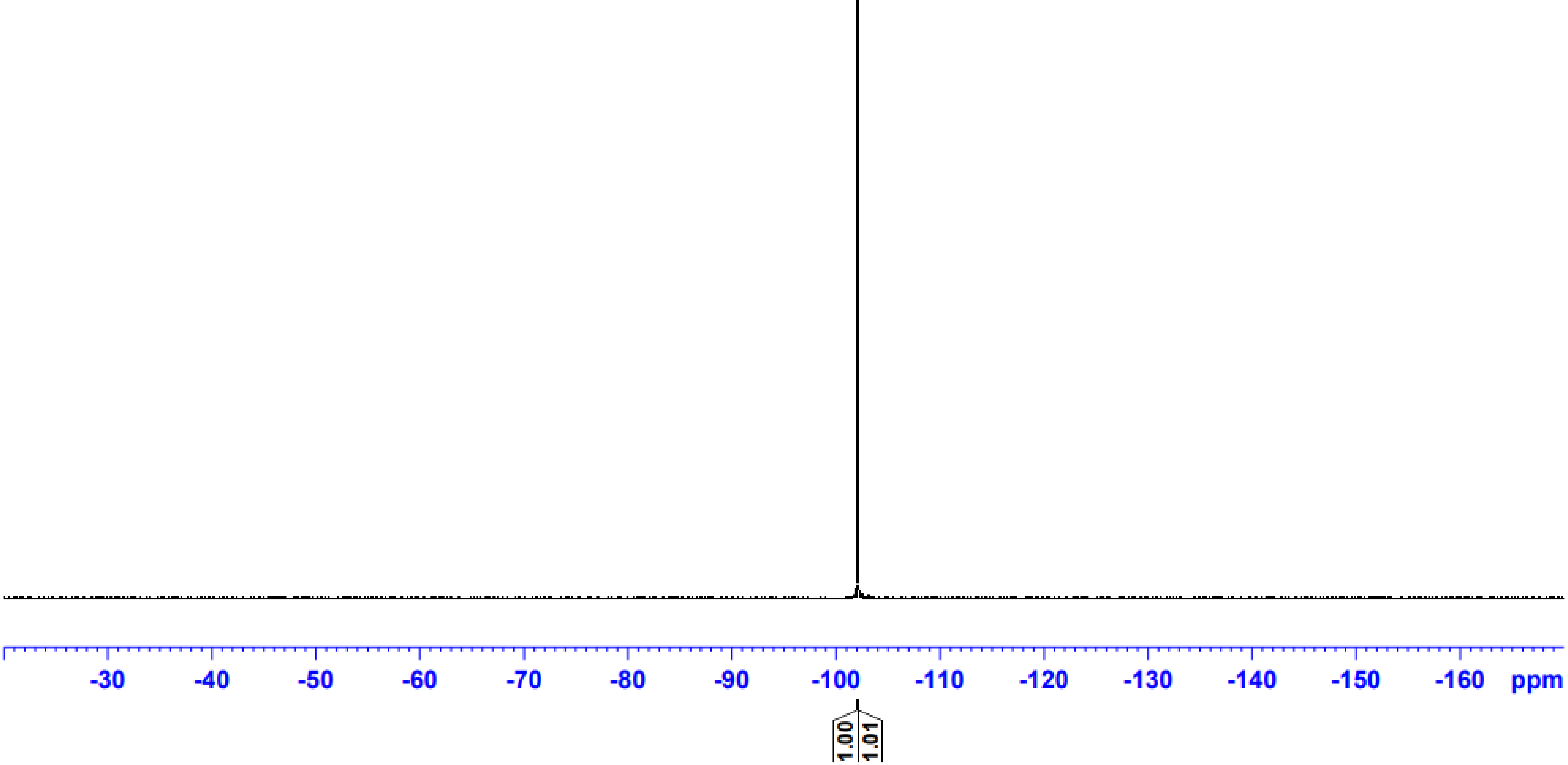

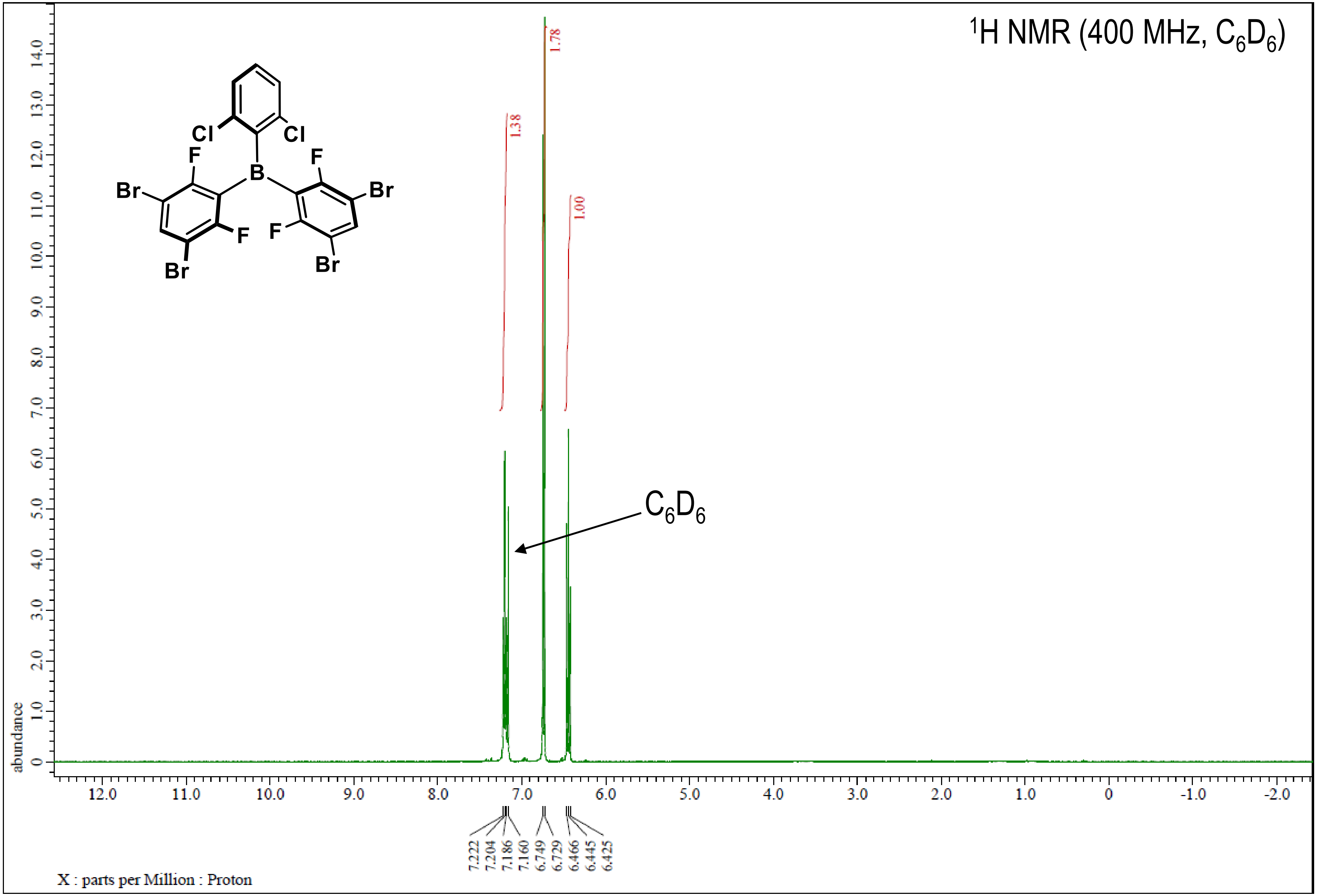

1H NMR (400 MHz, C6D6)
Cl
Cl
F
F
Br
B
Br
F
F
Br
Br
C6D6
1.38
1.78
1.00
7.222
7.204
7.186
7.160
6.749
6.729
6.466
6.445
6.425
abundance
X : parts per Million : Proton

^{11}B NMR (128 MHz, C_6D_6)

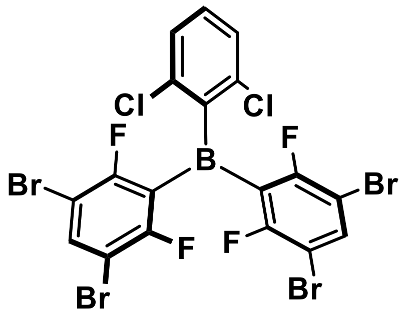

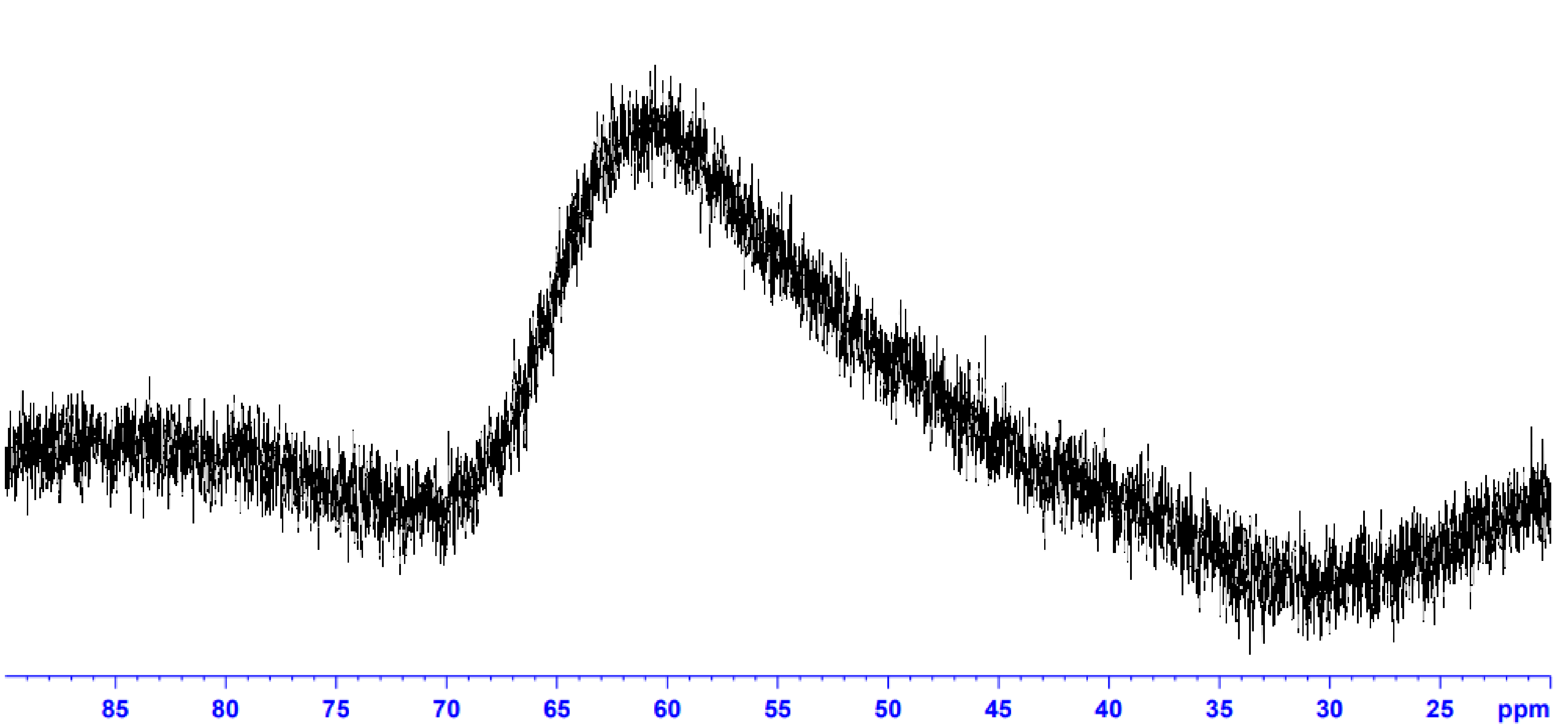

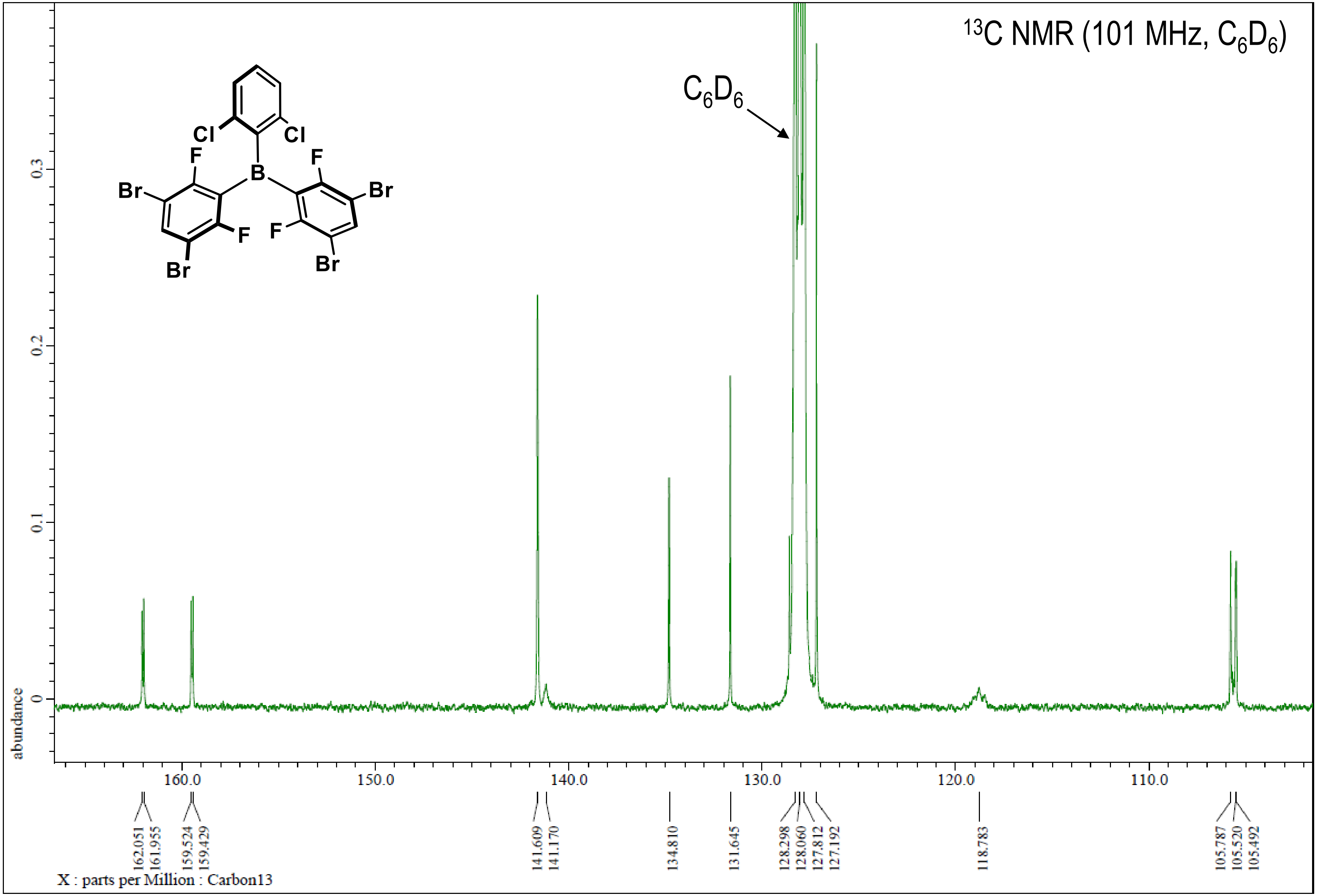

^{13}C NMR (101 MHz, C_6D_6)
C_6D_6
Cl
Cl
F
F
B
Br
Br
F
F
Br
Br
0.3
0.2
0.1
0
abundance
160.0
150.0
140.0
130.0
120.0
110.0
162.051
161.955
159.524
159.429
141.609
141.170
134.810
131.645
128.298
128.060
127.812
127.192
118.783
105.787
105.520
105.492
X : parts per Million : Carbon13

^{19}F NMR (376 MHz, C_6D_6)

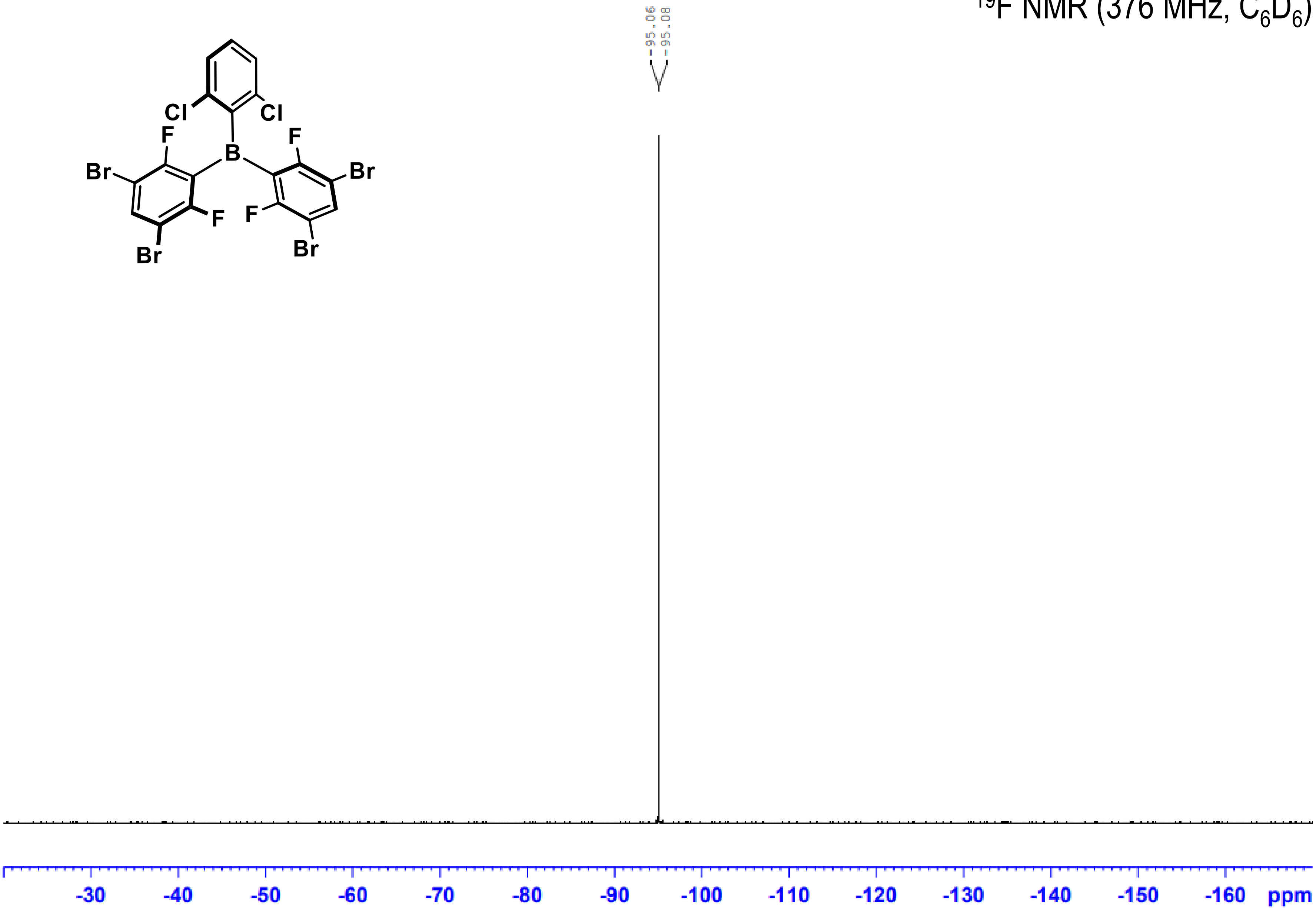

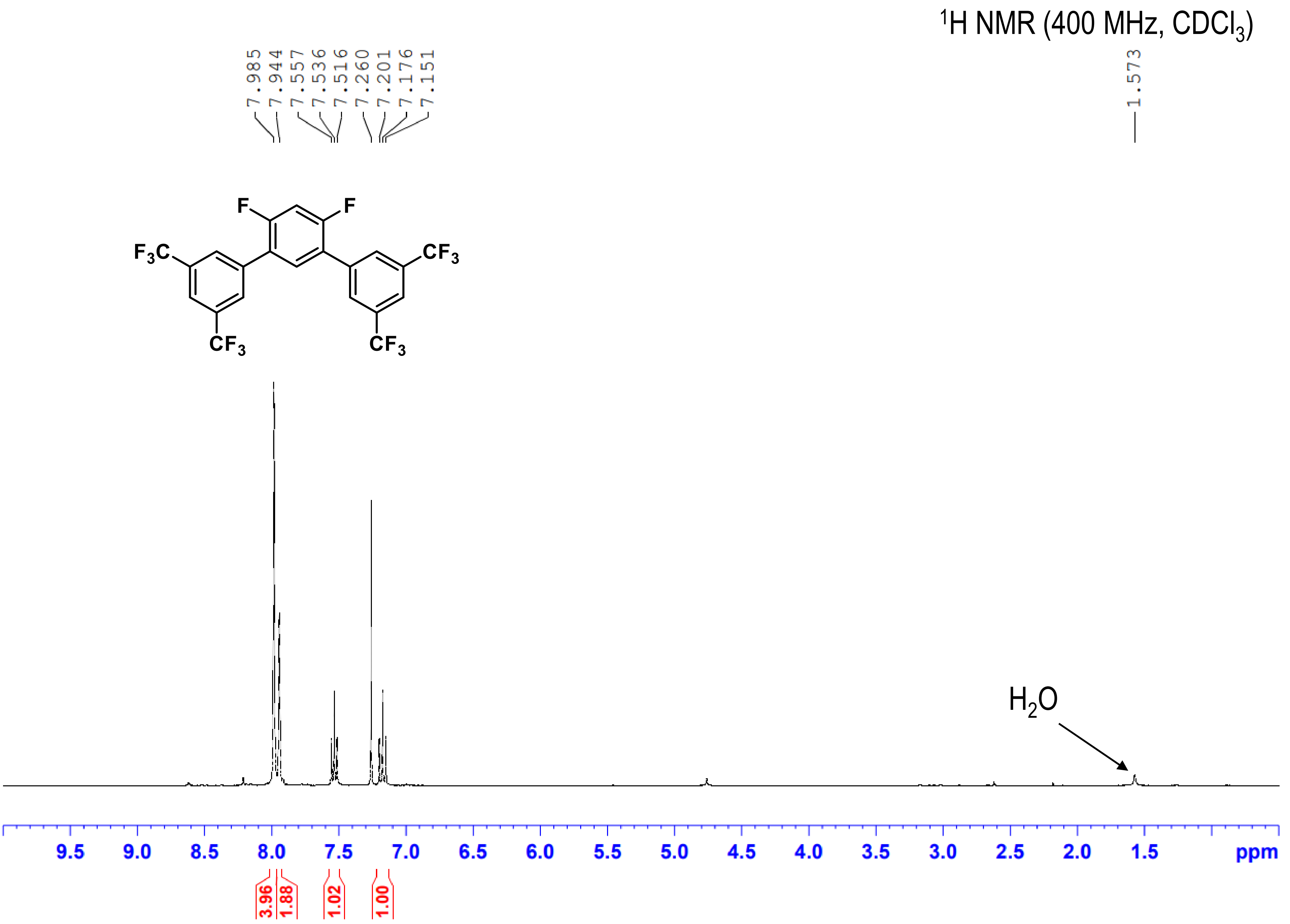

1H NMR (400 MHz, CDCl3)
7.985
7.944
7.557
7.536
7.516
7.260
7.201
7.176
7.151
1.573
F
F
F3C
CF3
CF3
CF3
H2O
9.5
9.0
8.5
8.0
7.5
7.0
6.5
6.0
5.5
5.0
4.5
4.0
3.5
3.0
2.5
2.0
1.5
ppm
3.96
1.88
1.02
1.00

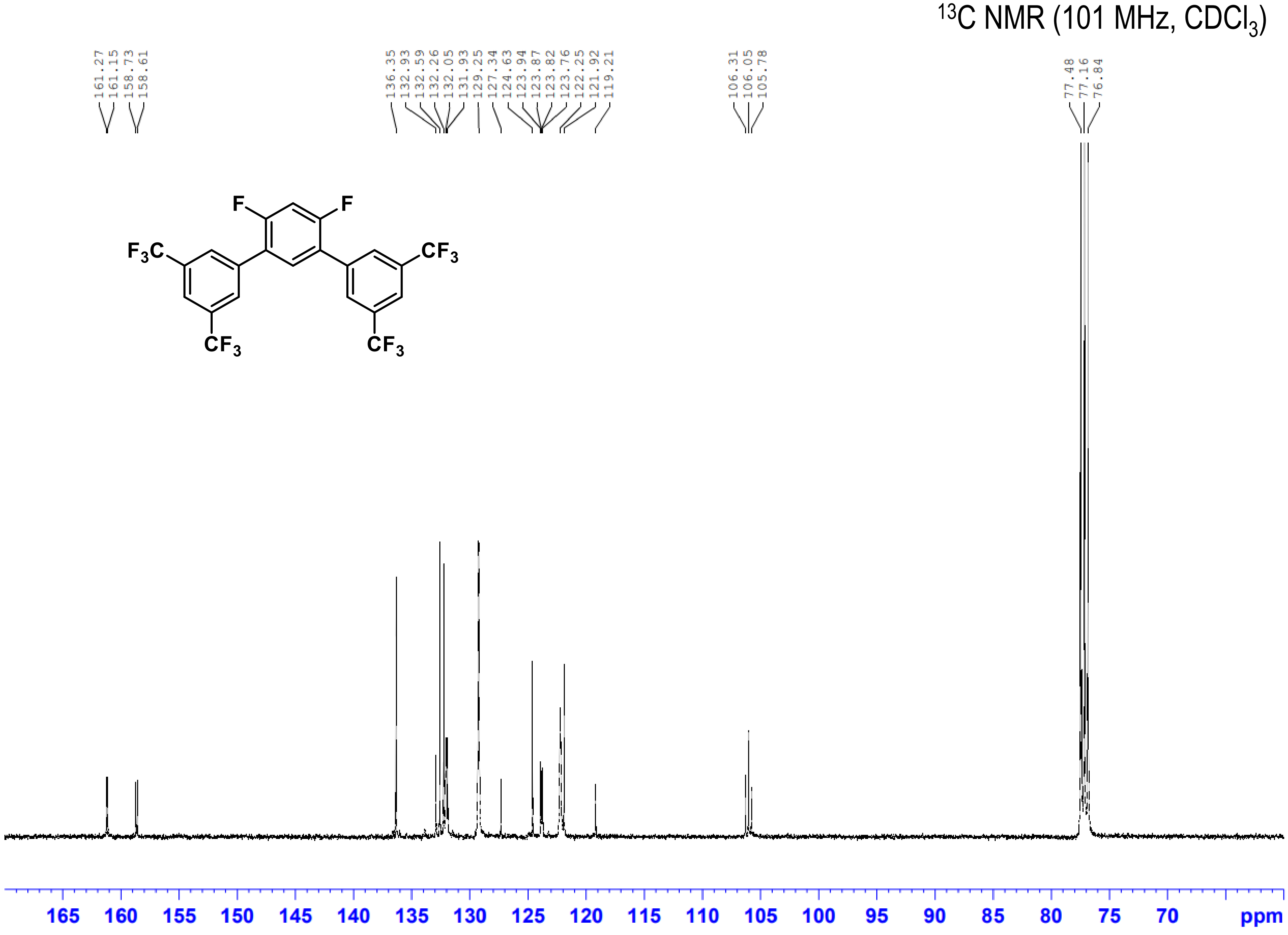

13C NMR (101 MHz, CDCl3)
161.27
161.15
158.73
158.61
136.35
132.93
132.59
132.26
132.05
131.93
129.25
127.34
124.63
123.94
123.87
123.82
123.76
122.25
121.92
119.21
106.31
106.05
105.78
77.48
77.16
76.84
F
F
F3C
CF3
CF3
CF3
165
160
155
150
145
140
135
130
125
120
115
110
105
100
95
90
85
80
75
70
ppm

^{19}F NMR (376 MHz, $CDCl_3$)

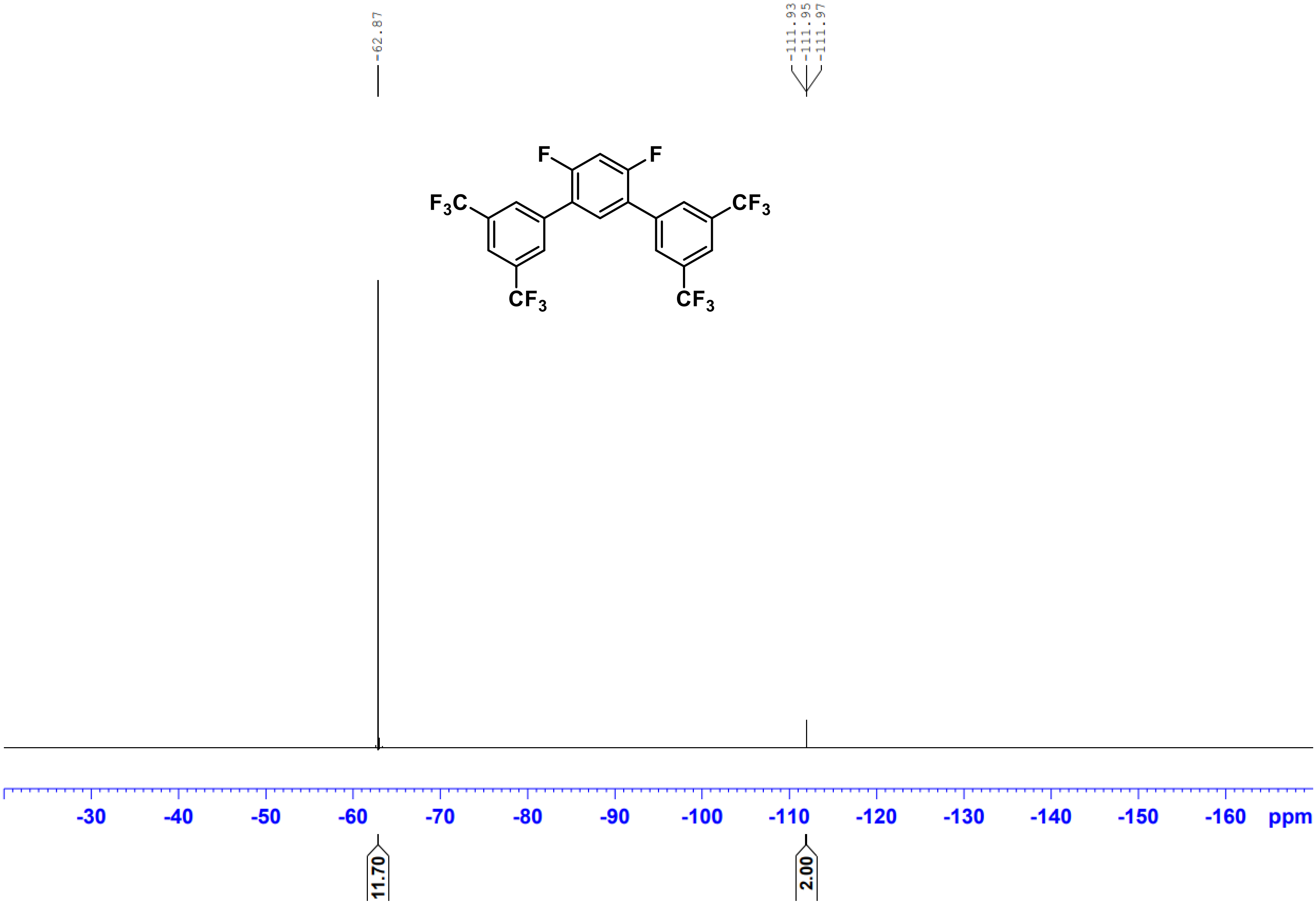

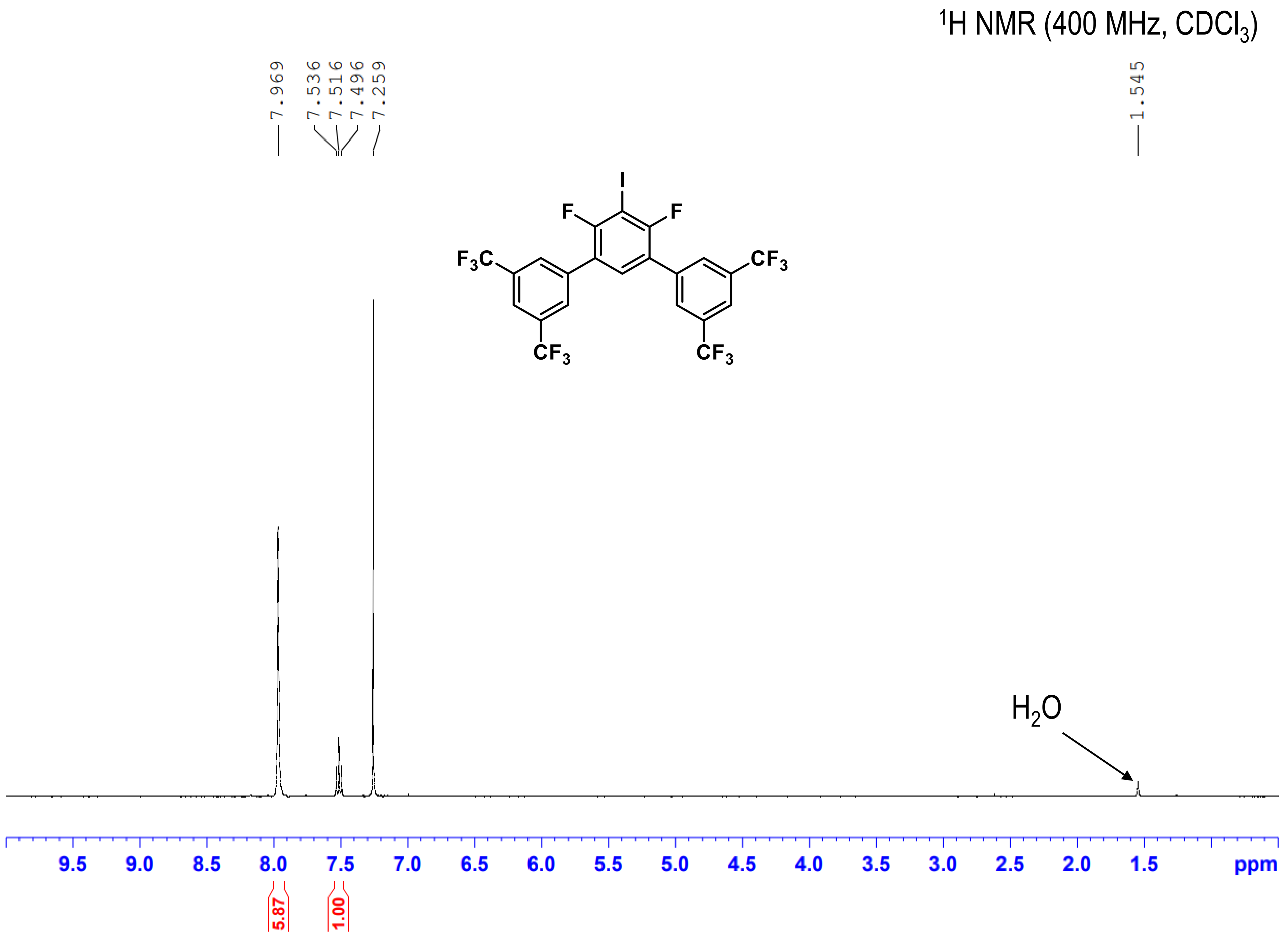

^1H NMR (400 MHz, $CDCl_3$)
7.969
7.536
7.516
7.496
7.259
1.545
I
F
F
F_3C
CF_3
CF_3
CF_3
H_2O
9.5
9.0
8.5
8.0
7.5
7.0
6.5
6.0
5.5
5.0
4.5
4.0
3.5
3.0
2.5
2.0
1.5
ppm
5.87
1.00

^{13}C NMR (101 MHz, $CDCl_3$)

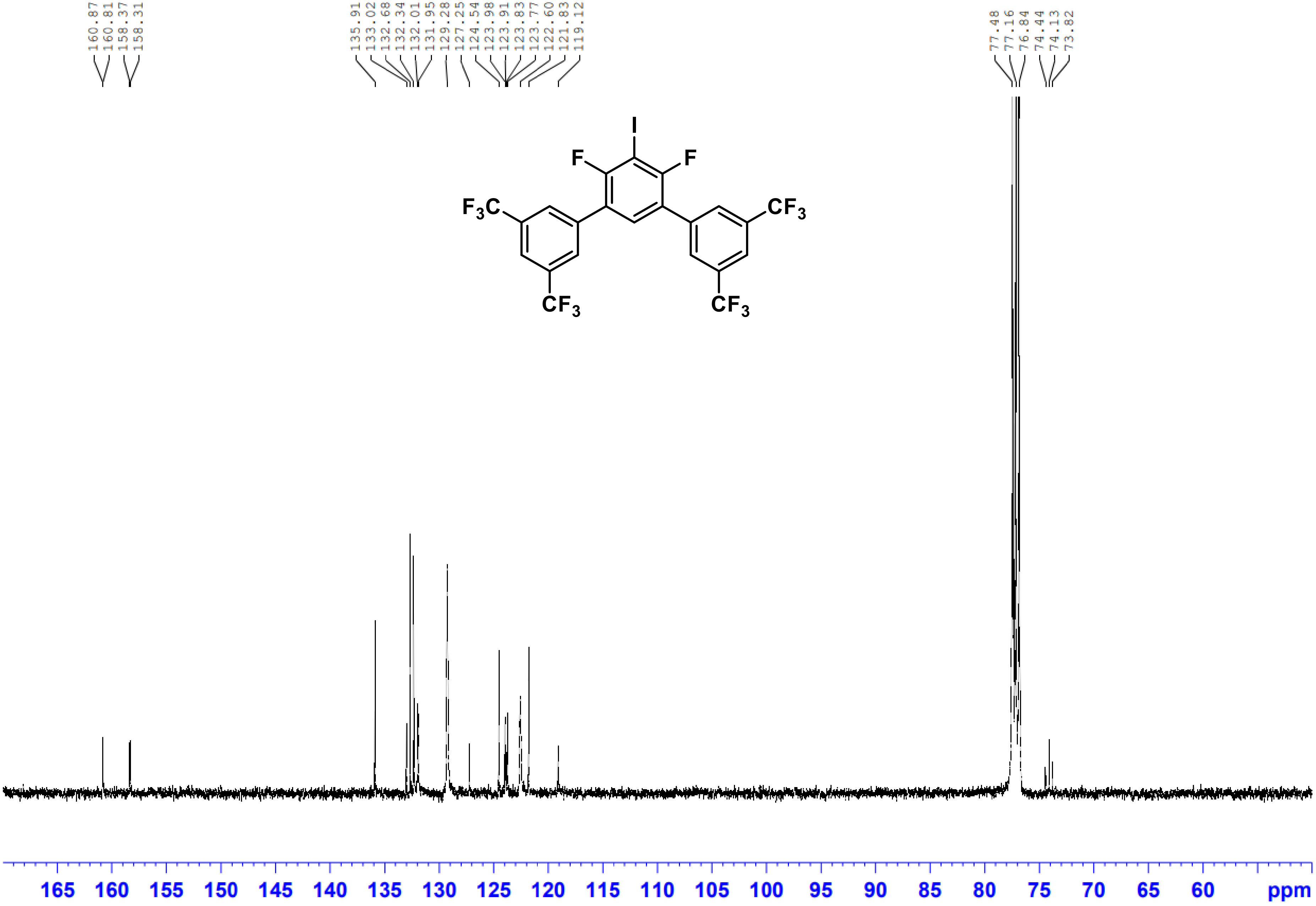

^{19}F NMR (376 MHz, $CDCl_3$)

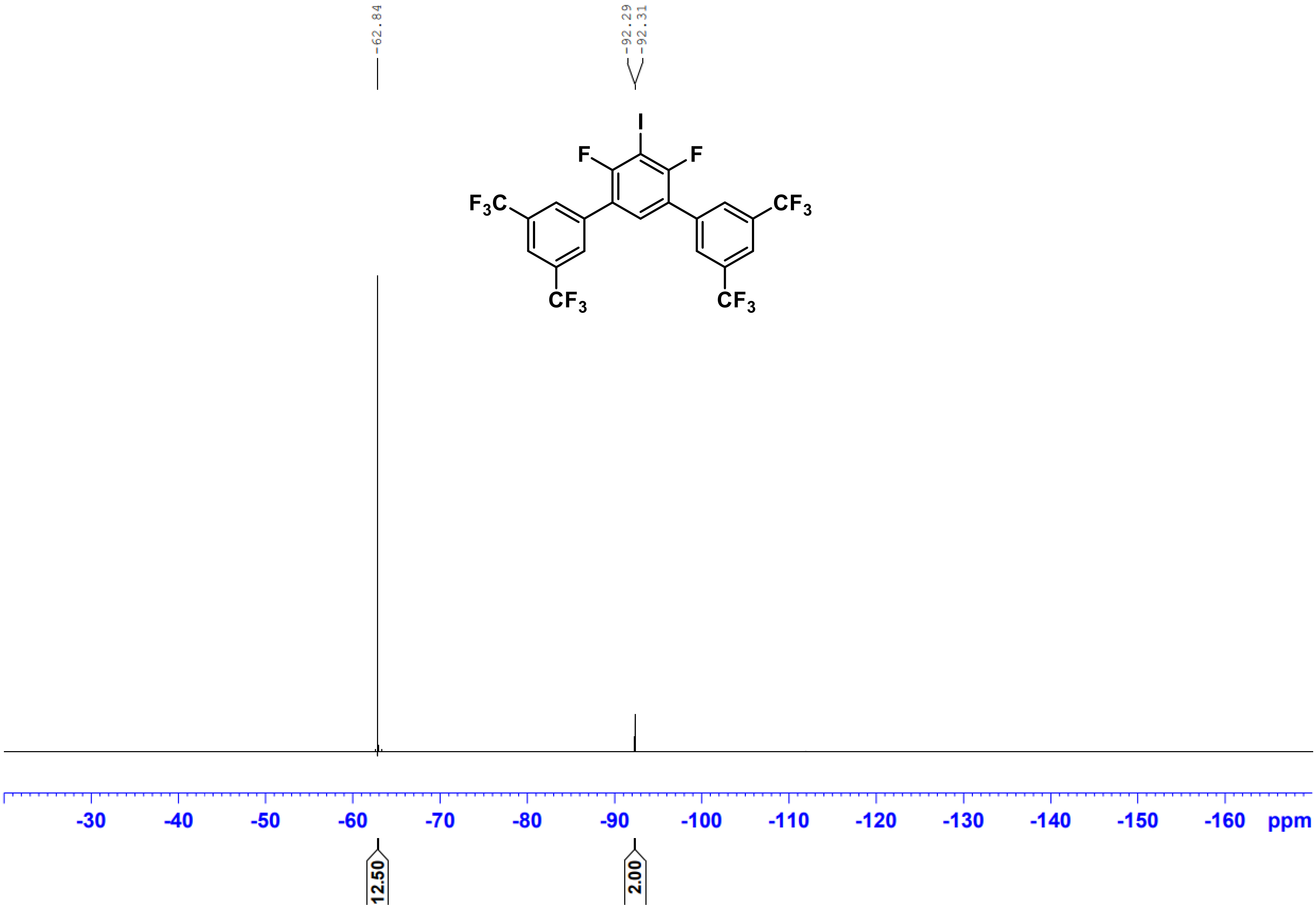

^{1}H NMR (400 MHz, $CDCl_3$)

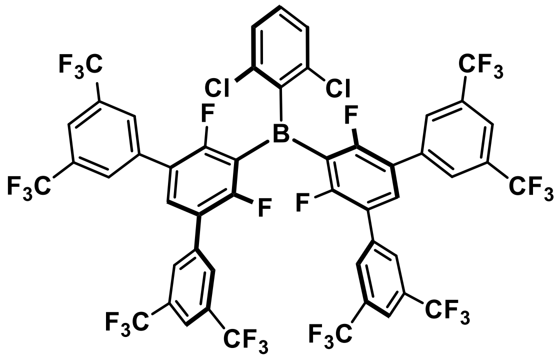

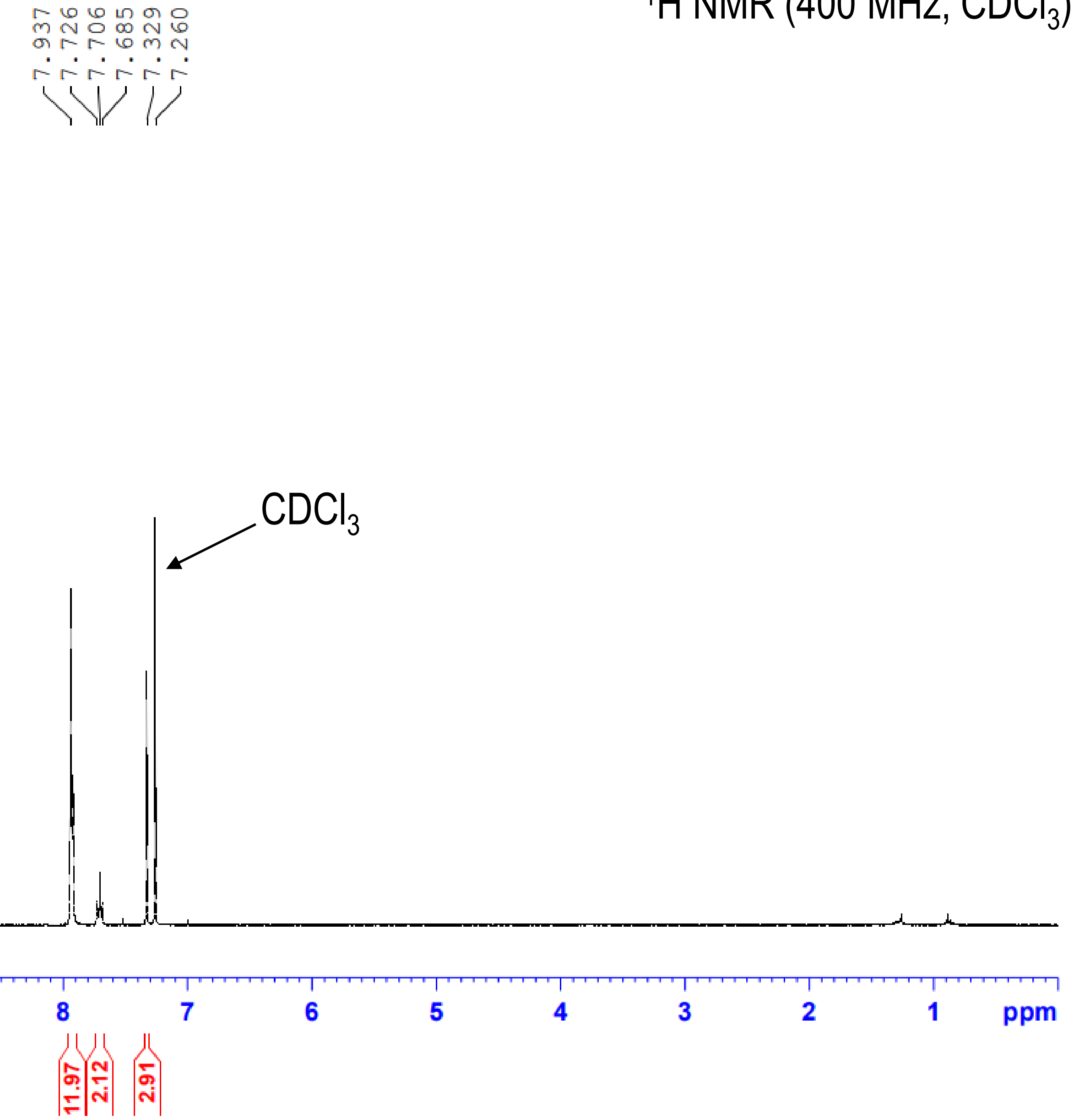

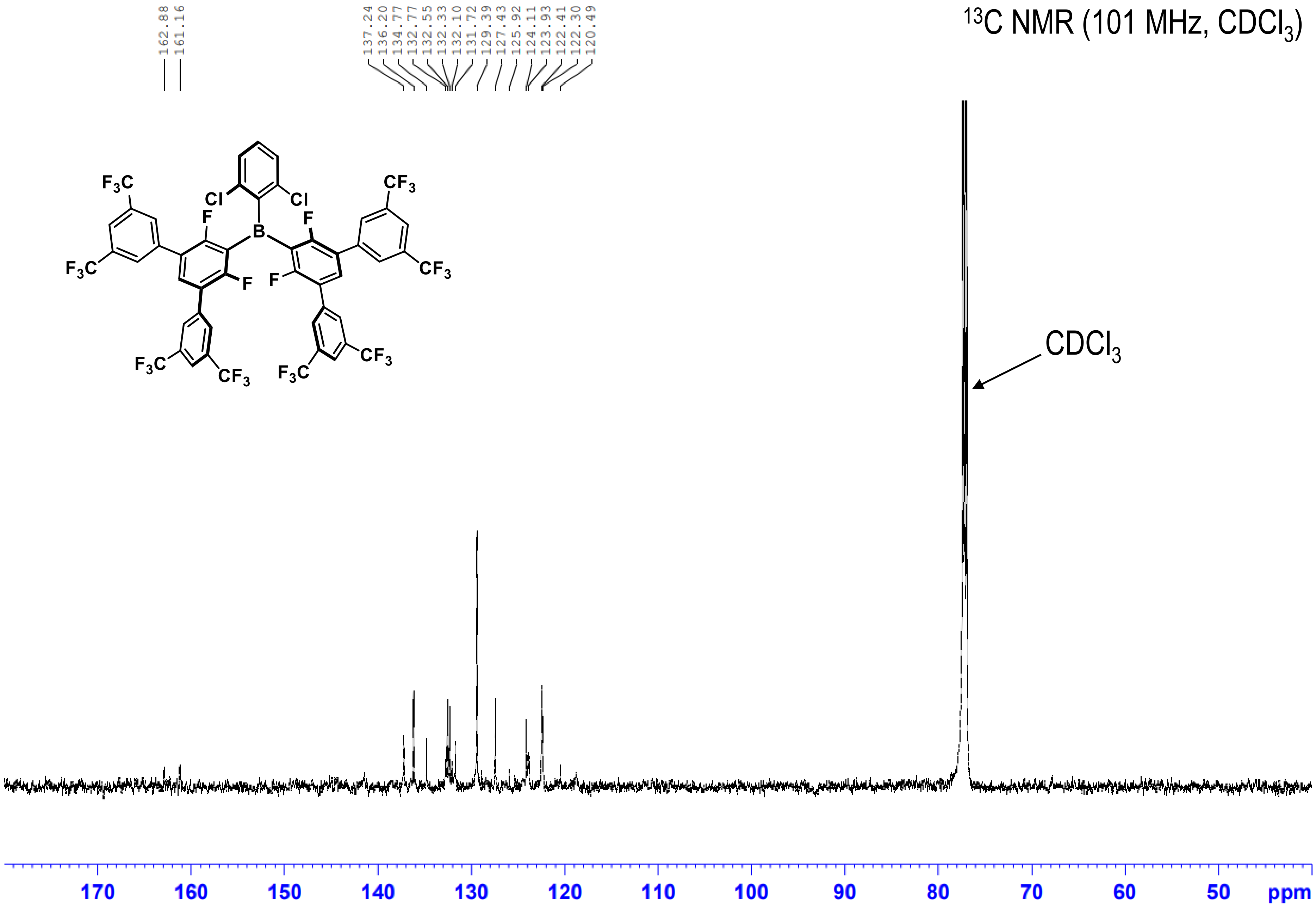

13C NMR (101 MHz, CDCl3)
162.88
161.16
137.24
136.20
134.77
132.77
132.55
132.33
132.10
131.72
129.39
127.43
125.92
124.11
123.93
122.41
122.30
120.49
CDCl3
F3C
CF3
Cl
Cl
F
F
B
F
F
F3C
CF3
F3C
CF3
F3C
CF3
170
160
150
140
130
120
110
100
90
80
70
60
50
ppm

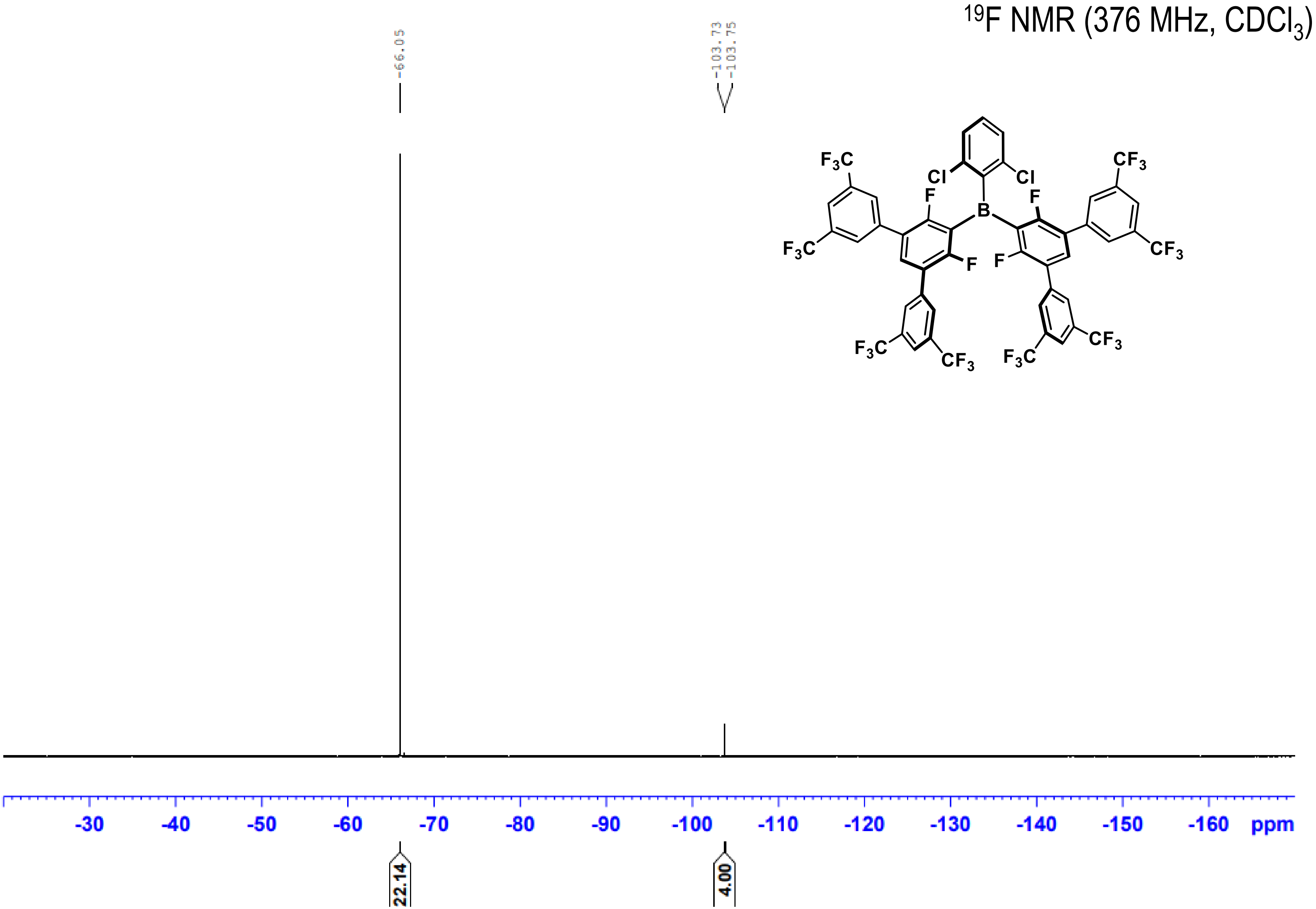

19F NMR (376 MHz, CDCl3)
-66.05
-103.73
-103.75
F3C
CF3
Cl
Cl
F
F
B
F
F
F3C
CF3
F3C
CF3
F3C
CF3
-30
-40
-50
-60
-70
-80
-90
-100
-110
-120
-130
-140
-150
-160
ppm
22.14
4.00

^{1}H NMR (400 MHz, CD_2Cl_2)

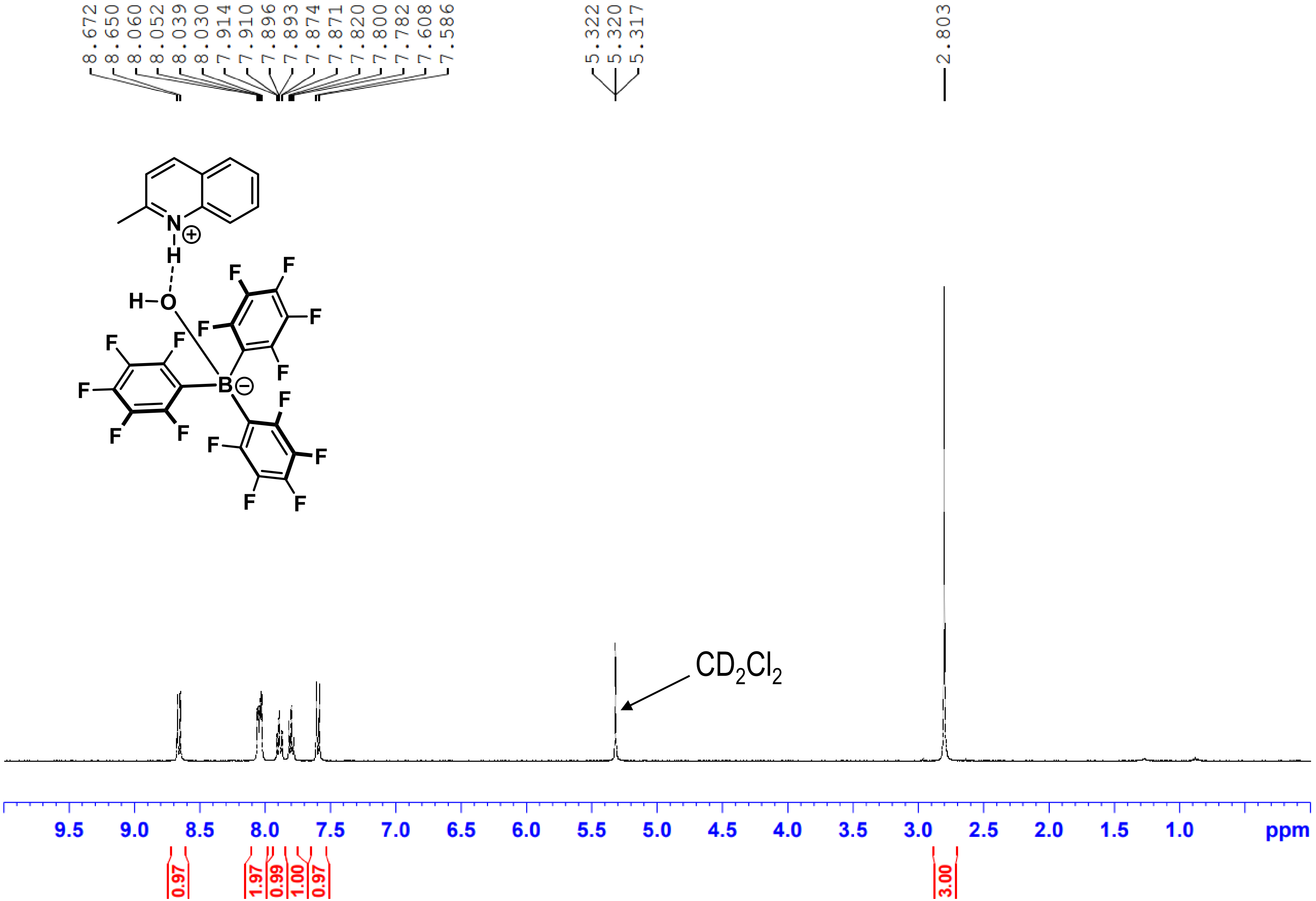

^{11}B NMR (128 MHz, CD_2Cl_2)

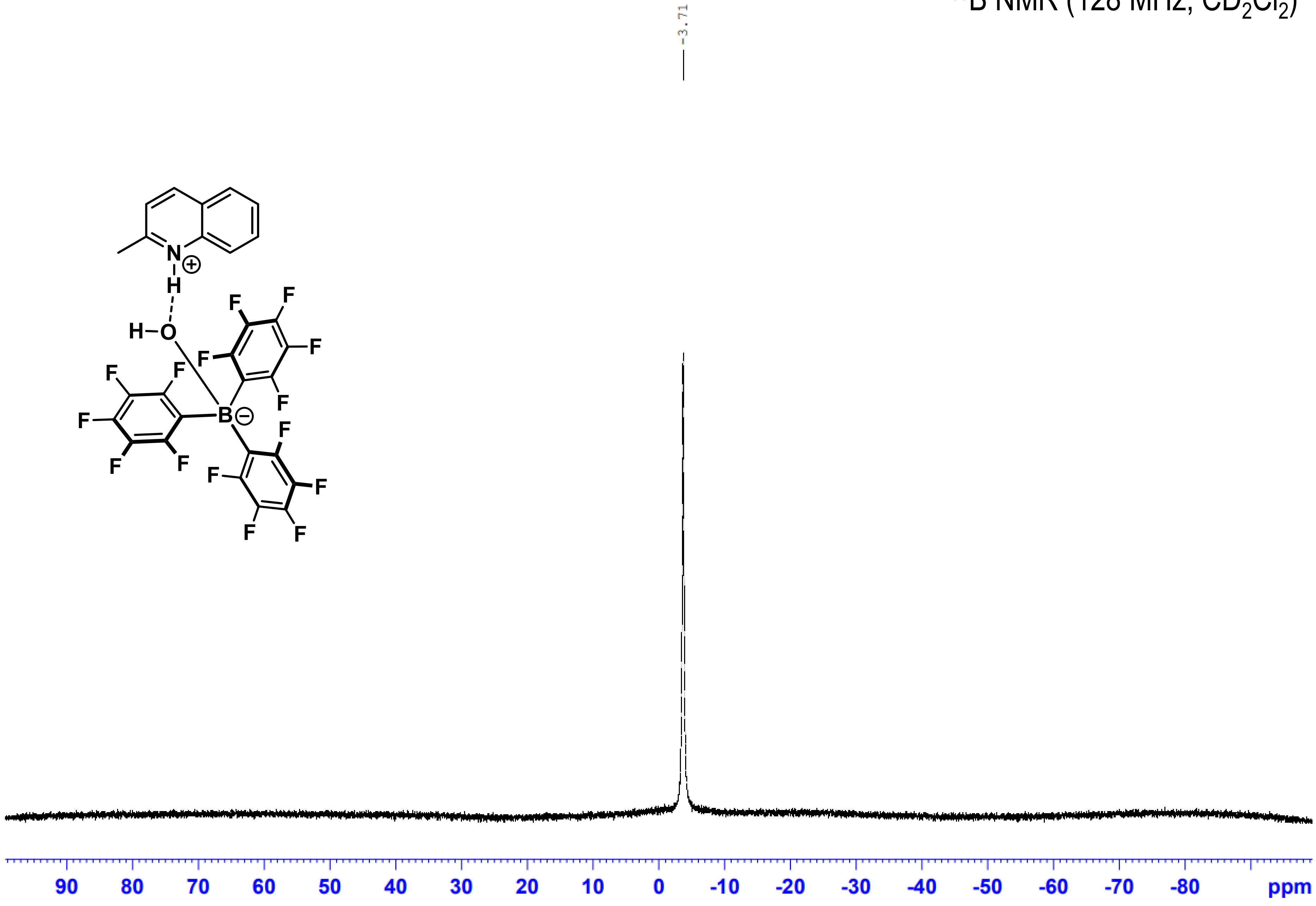

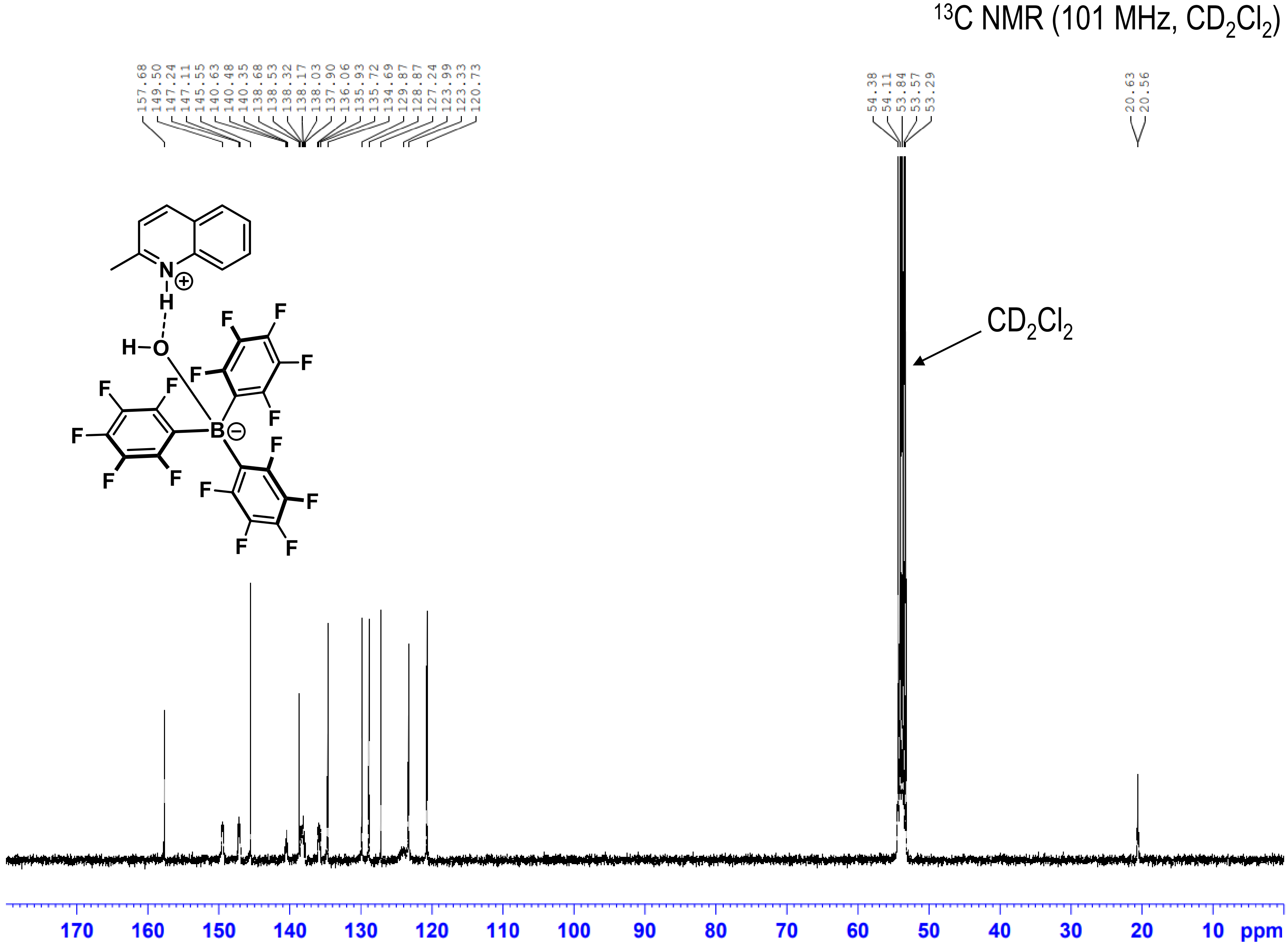

13C NMR (101 MHz, CD2Cl2)
157.68
149.50
147.24
147.11
145.55
140.63
140.48
140.35
138.68
138.53
138.32
138.17
138.03
137.90
136.06
135.93
135.72
134.69
129.87
128.87
127.24
123.99
123.33
120.73
54.38
54.11
53.84
53.57
53.29
20.63
20.56
CD2Cl2
170
160
150
140
130
120
110
100
90
80
70
60
50
40
30
20
10
ppm

^{19}F NMR (376 MHz, CD_2Cl_2)

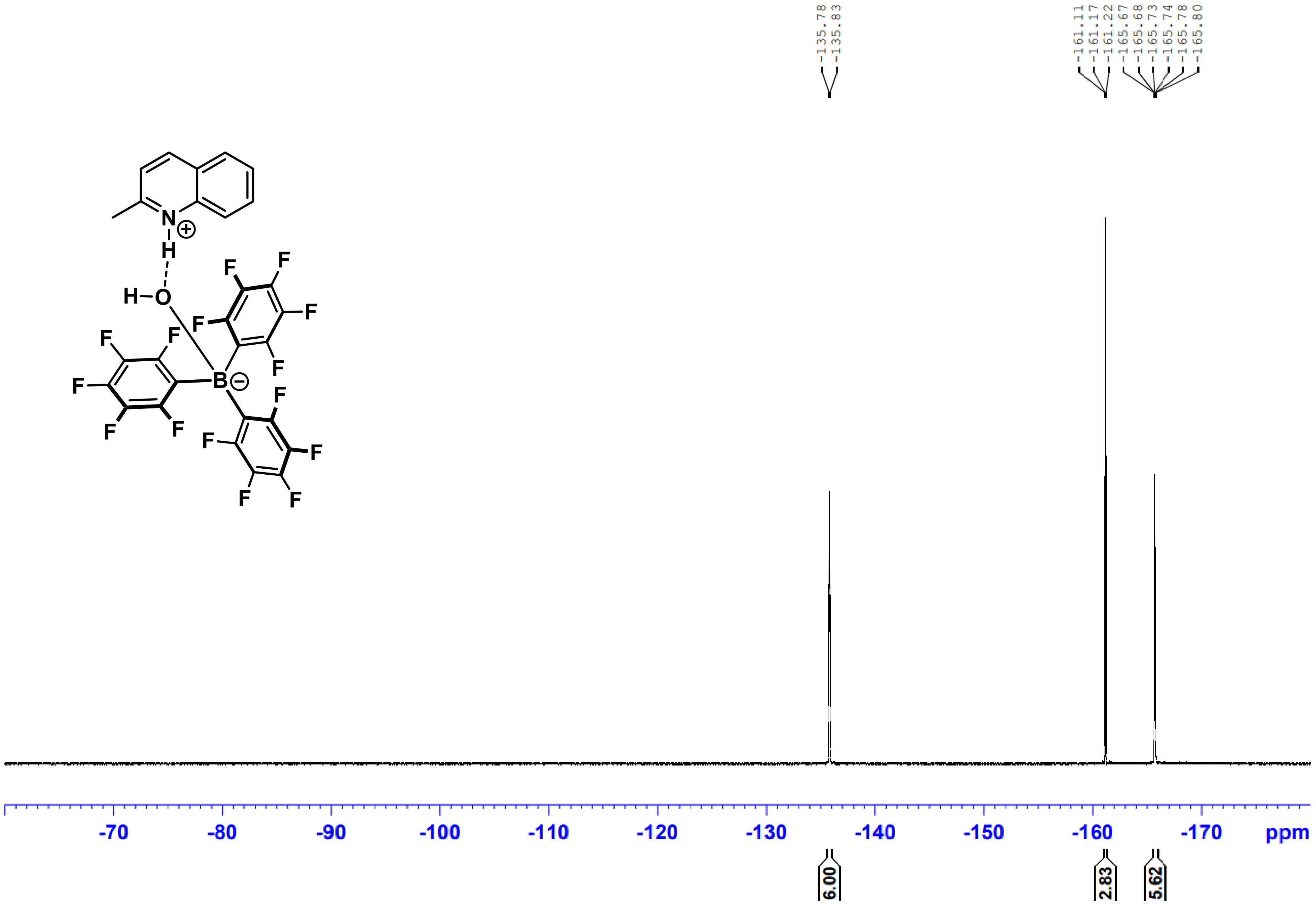

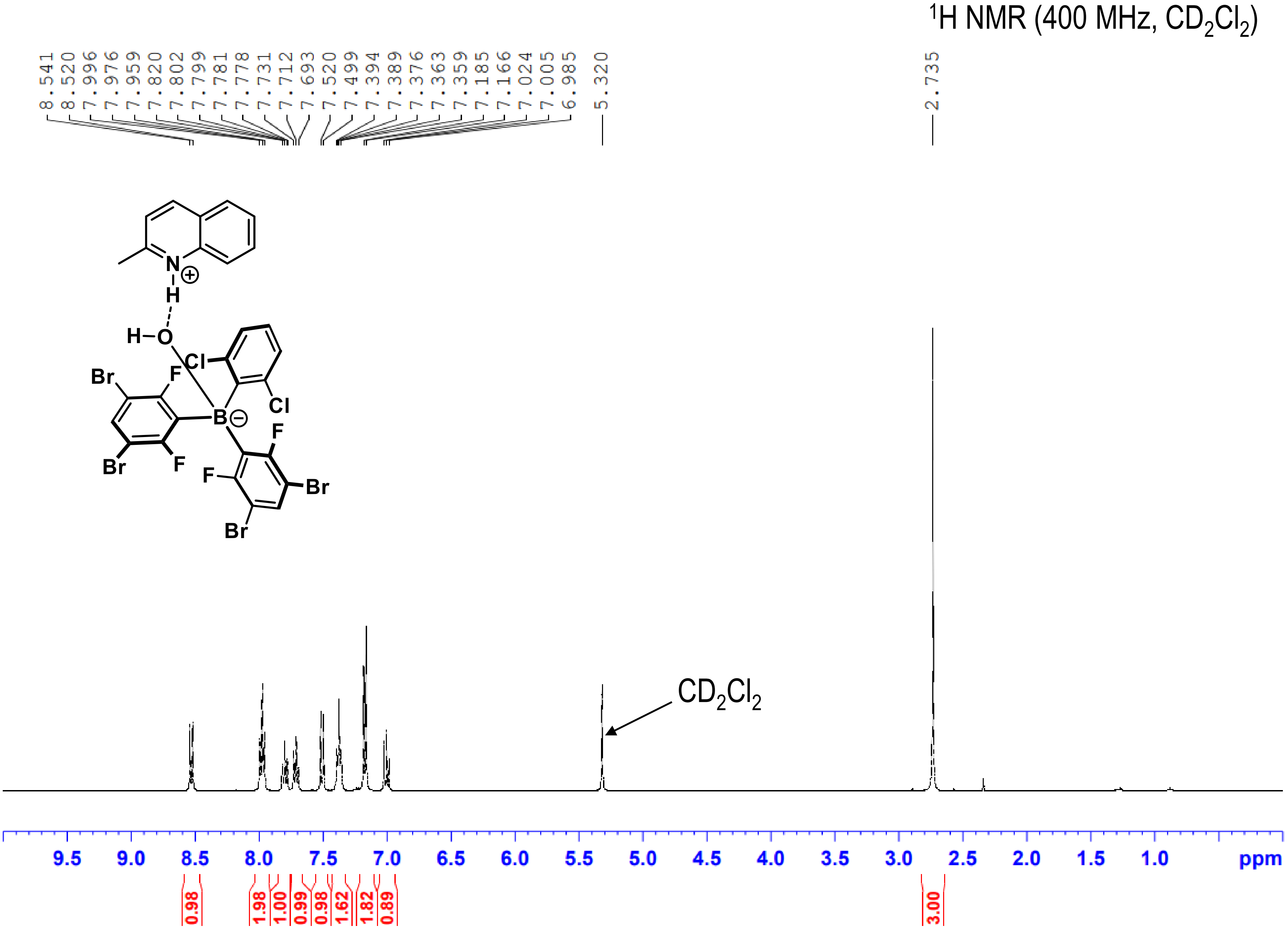

1H NMR (400 MHz, CD2Cl2)
8.541
8.520
7.996
7.976
7.959
7.820
7.802
7.799
7.781
7.778
7.731
7.712
7.693
7.520
7.499
7.394
7.389
7.376
7.363
7.359
7.185
7.166
7.024
7.005
6.985
5.320
2.735
N
H
H
O
Cl
Cl
Br
F
B
F
Br
F
F
Br
Br
CD2Cl2
9.5
9.0
8.5
8.0
7.5
7.0
6.5
6.0
5.5
5.0
4.5
4.0
3.5
3.0
2.5
2.0
1.5
1.0
ppm
0.98
1.98
1.00
0.99
0.98
1.62
1.82
0.89
3.00

^{11}B NMR (128 MHz, CD_2Cl_2)

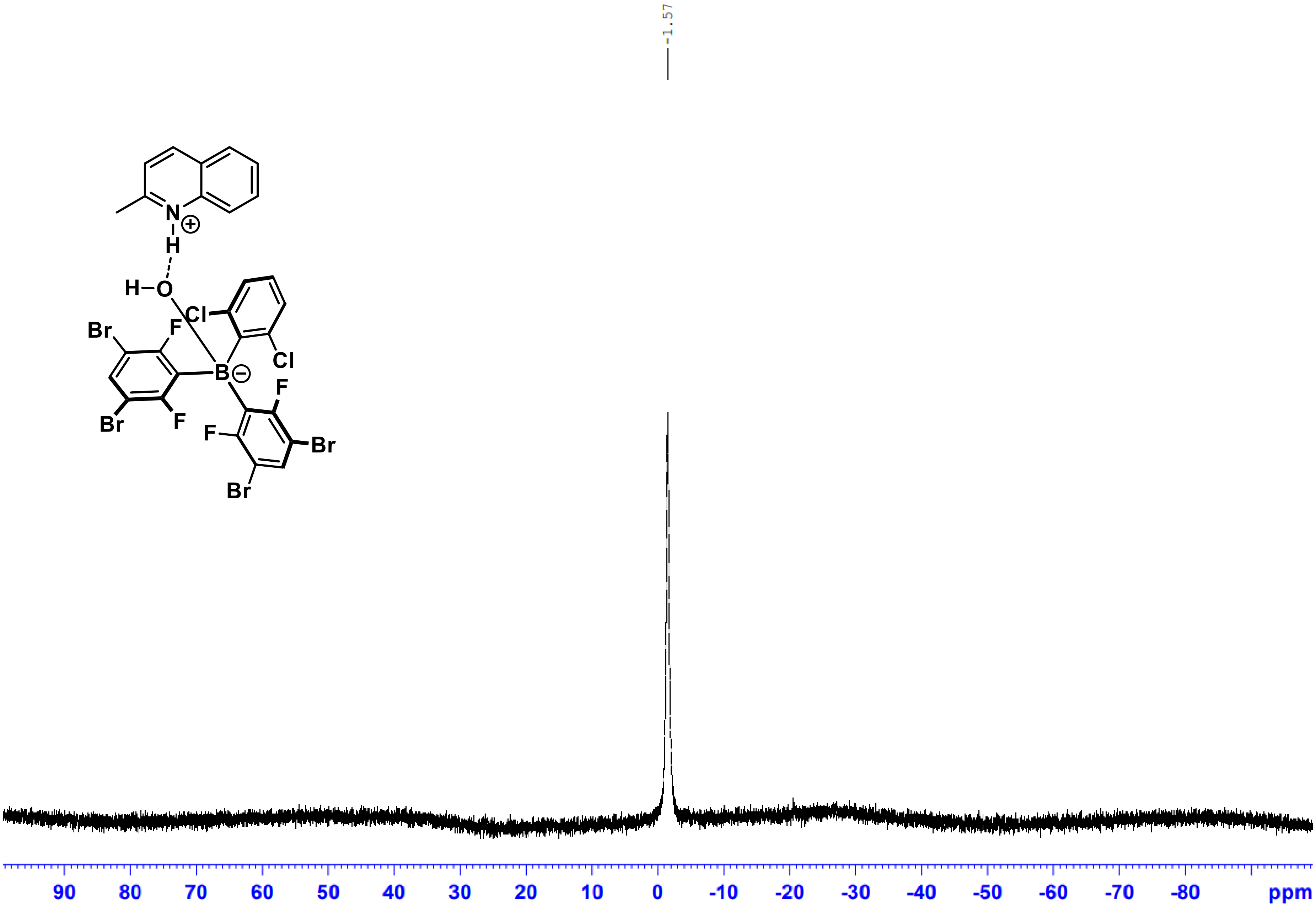

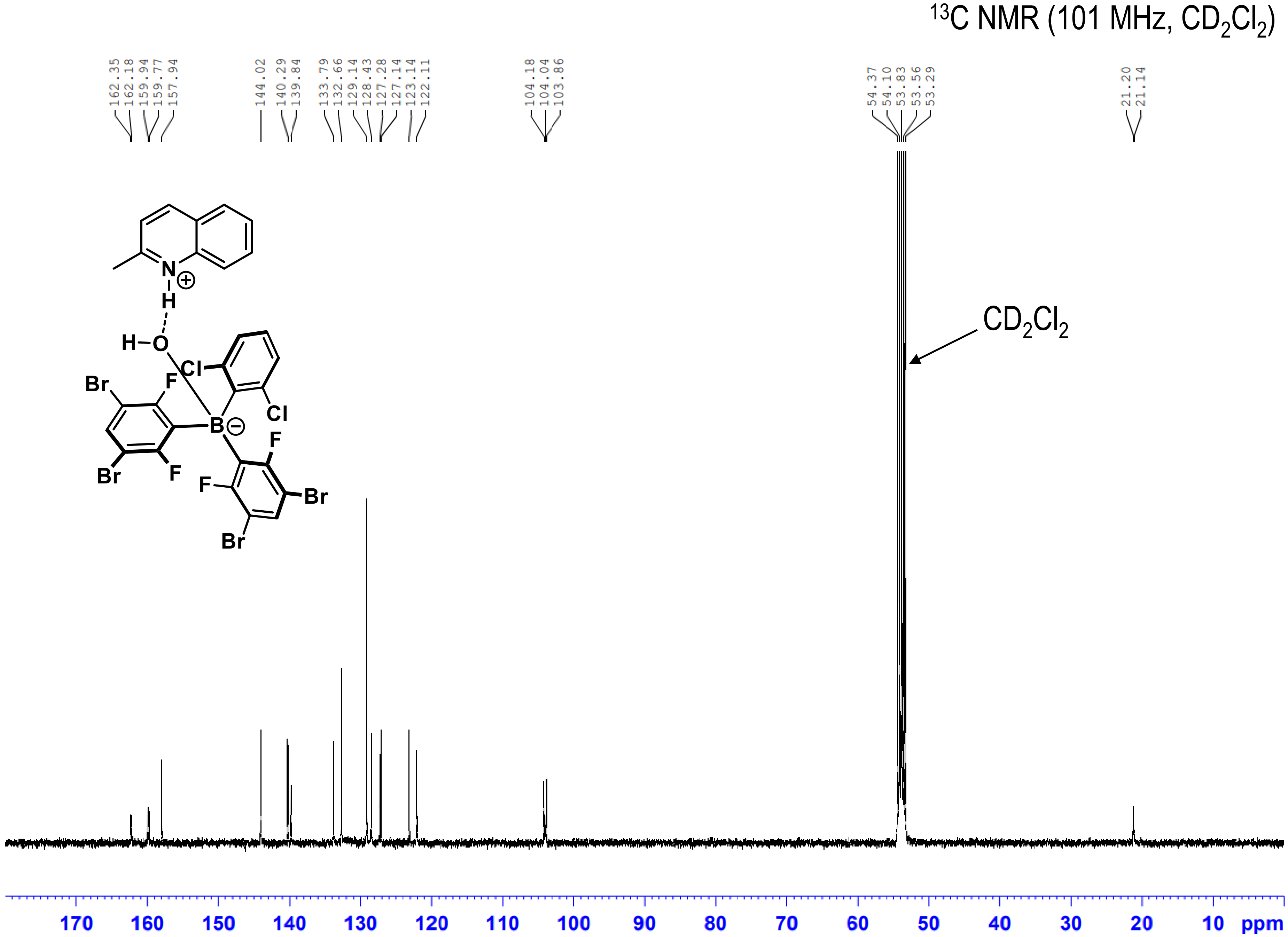

^{13}C NMR (101 MHz, CD_2Cl_2)
162.35
162.18
159.94
159.77
157.94
144.02
140.29
139.84
133.79
132.66
129.14
128.43
127.28
127.14
123.14
122.11
104.18
104.04
103.86
54.37
54.10
53.83
53.56
53.29
21.20
21.14
CD_2Cl_2
170 160 150 140 130 120 110 100 90 80 70 60 50 40 30 20 10 ppm

^{19}F NMR (376 MHz, CD_2Cl_2)

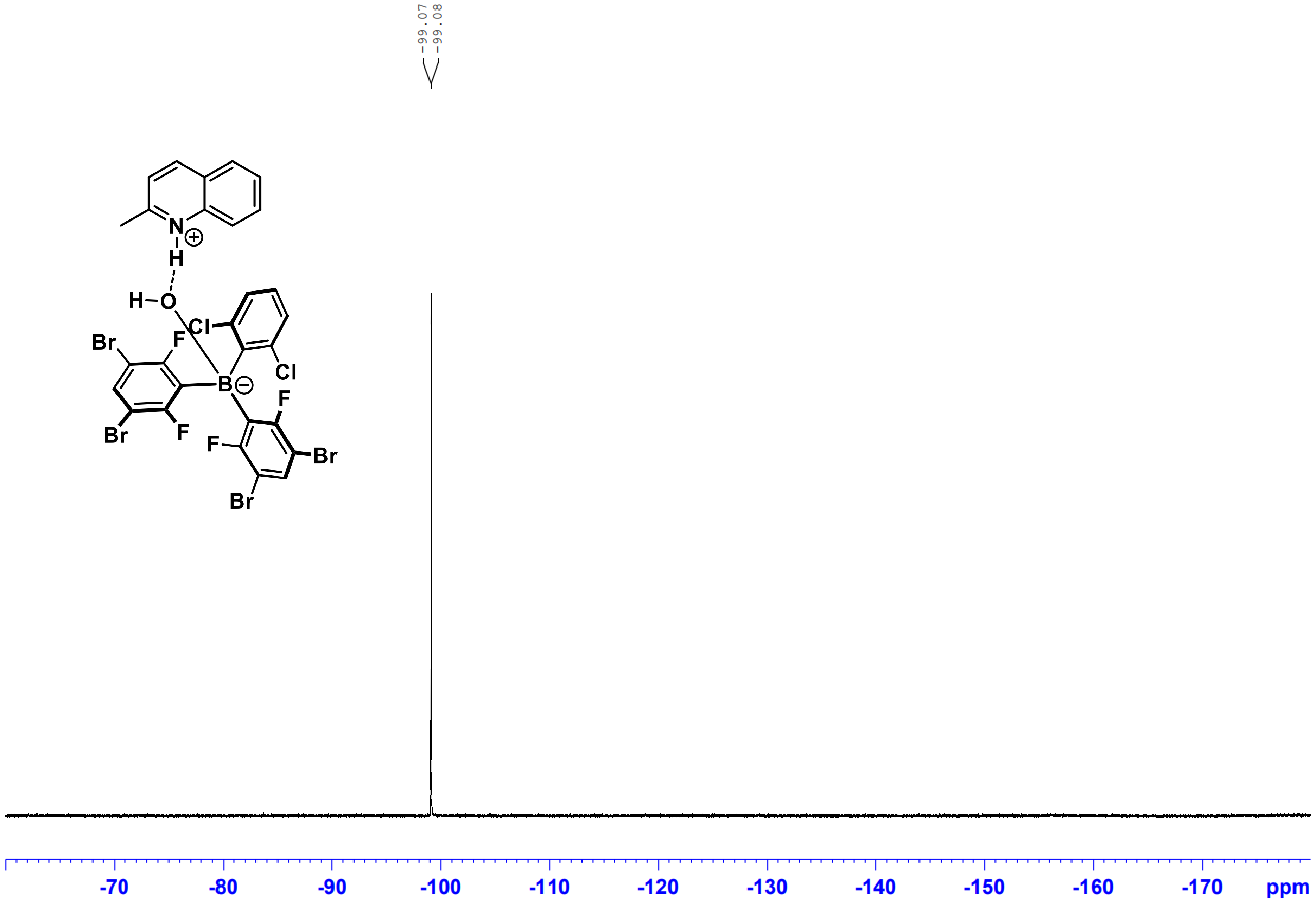

REFERENCES AND NOTES

1. K. Liu, C. Song, V. Subramani, Eds., *Hydrogen and Syngas Production and Purification Technologies* (Wiley, 2010).

2. F. Dawood, M. Anda, G. M. Shafiullah, Hydrogen production for energy: An overview. *Int. J. Hydrogen Energy* **45**, 3847–3869 (2020).

3. M. Voldsund, K. Jordal, R. Anantharaman, Hydrogen production with CO_2 capture. *Int. J. Hydrogen Energy* **41**, 4969–4992 (2016).

4. J. D. Holladay, J. Hu, D. L. King, Y. Wang, An overview of hydrogen production technologies. *Catal. Today* **139**, 244–260 (2009).

5. H. Nishiyama, T. Yamada, M. Nakabayashi, Y. Maehara, M. Yamaguchi, Y. Kuromiya, Y. Nagatsuma, H. Tokudome, S. Akiyama, T. Watanabe, R. Narushima, S. Okunaka, N. Shibata, T. Takata, T. Hisatomi, K. Domen, Photocatalytic solar hydrogen production from water on a 100-m^2 scale. *Nature* **598**, 304–307 (2021).

6. Y. Okada, M. Saito, S. Wakayama, M. Shimura, Method for producing hydrogen aimed at storage and transportation, Patent US8758722 (2014); https://patents.google.com/patent/JP5737853B2/en.

7. Y. Hoshimoto, S. Ogoshi, T. Tanaka, N. Kawamoto, Method for hydrogenating unsaturated compound, Patent JP2017206474A (2017); https://patents.google.com/patent/JP2017206474A/en.

8. E. Gianotti, M. Taillades-Jacquin, J. Rozière, D. J. Jones, High-purity hydrogen generation via dehydrogenation of organic carriers: A review on the catalytic process. *ACS Catal.* **8**, 4660–4680 (2018).

9. H. Jorschick, P. Preuster, A. Bösmann, P. Wasserscheid, Hydrogenation of aromatic and heteroaromatic compounds–A key process for future logistics of green hydrogen using liquid organic hydrogen carrier systems. *Sustain. Energy Fuels* **5**, 1311–1346 (2021).

10. Y.-Q. Zou, N. von Wolff, A. Anaby, Y. Xie, D. Milstein, Ethylene glycol as an efficient and reversible liquid-organic hydrogen carrier. *Nat. Catal.* **2**, 415–422 (2019).

11. T. Shimbayashi, K.-i. Fujita, Metal-catalyzed hydrogenation and dehydrogenation reactions for efficient hydrogen storage. *Tetrahedron* **76**, 130946 (2020).

12. T. He, Q. Pei, P. Chen, Liquid organic hydrogen carriers. *J. Energy Chem.* **24**, 587–594 (2015).

13. K.-i. Fujita, T. Wada, T. Shiraishi, Reversible interconversion between 2,5-dimethylpyrazine and 2,5-dimethylpiperazine by iridium-catalyzed hydrogenation/dehydrogenation for efficient hydrogen storage. *Angew. Chem. Int. Ed.* **56**, 10886–10889 (2017).

14. P. Hu, E. Fogler, Y. Diskin-Posner, M. A. Iron, D. Milstein, A novel liquid organic hydrogen carrier system based on catalytic peptide formation and hydrogenation. *Nat. Commun.* **6**, 6859 (2015).

15. P. Hu, Y. Ben-David, D. Milstein, Rechargeable hydrogen storage system based on the dehydrogenative coupling of ethylenediamine with ethanol. *Angew. Chem. Int. Ed.* **55**, 1061–1064 (2016).

16. J. Kothandaraman, S. Kar, R. Sen, A. Goeppert, G. A. Olah, G. K. Surya Prakash, Efficient reversible hydrogen carrier system based on amine reforming of methanol. *J. Am. Chem. Soc.* **139**, 2549–2552 (2017).

17. Y. Xie, P. Hu, Y. Ben-David, D. Milstein, A reversible liquid organic hydrogen carrier system based on methanol-ethylenediamine and ethylene urea. *Angew. Chem. Int. Ed.* **58**, 5105–5109 (2019).

18. C. Breakman-Danheux, A. Fontana, P. Laurent, P. Lolivier, Catalytic hydrogenation of polycyclic aromatic hydrocarbons with coke oven gas. *Fuel* **75**, 579–584 (1996).

19. H. Jorschick, M. Vogl, P. Preuster, A. Bösmann, P. Wasserscheid, Hydrogenation of liquid organic hydrogen carrier systems using multicomponent gas mixtures. *Int. J. Hydrogen Energy* **44**, 31172–31182 (2019).

20. K. Revunova, G. I. Nikonov, Main group catalysed reduction of unsaturated bonds. *Dalton Trans.* **44**, 840–866 (2015).

21. M. Oestreich, J. Hermeke, J. Mohr, A unified survey of Si–H and H–H bond activation catalysed by electron-deficient boranes. *Chem. Soc. Rev.* **44**, 2202–2220 (2015).

22. G. C. Welch, R. R. S. Juan, J. D. Masuda, D. W. Stephan, Reversible, metal-free hydrogen activation. *Science* **314**, 1124–1126 (2006).

23. A. R. Jupp, D. W. Stephan, New directions for frustrated Lewis pair chemistry. *Trends Chem.* **1**, 35–48 (2019).

24. D. W. Stephan, Diverse uses of the reaction of frustrated Lewis pair (FLP) with hydrogen. *J. Am. Chem. Soc.* **143**, 20002–20014 (2021).

25. S. J. Geier, P. A. Chase, D. W. Stephan, Metal-free reductions of N-heterocycles via Lewis acid catalyzed hydrogenation. *Chem. Commun.* **46**, 4884–4886 (2010).

26. G. Erős, K. Nagy, H. Mehdi, I. Pápai, P. Nagy, P. Király, G. Tárkányi, T. Soós, Catalytic hydrogenation with frustrated Lewis pairs: Selectivity achieved by size-exclusion design of Lewis acids. *Chem. A Eur. J.* **18**, 574–585 (2012).

27. D. J. Scott, M. J. Fuchter, A. E. Ashley, Metal-free hydrogenation catalyzed by an air-stable borane: Use of solvent as a frustrated Lewis base. *Angew. Chem. Int. Ed.* **53**, 10218–10222 (2014).

28. C. M. Mömming, E. Otten, G. Kehr, R. Fröhlich, S. Grimme, D. W. Stephan, G. Erker, Reversible metal-free carbon dioxide binding by frustrated Lewis pairs. *Angew. Chem. Int. Ed.* **48**, 6643–6646 (2009).

29. D. W. Stephan, G. Erker, Frustrated Lewis pair chemistry of carbon, nitrogen and sulfur oxides. *Chem. Sci.* **5**, 2625–2641 (2014).

30. T. Voss, T. Mahdi, E. Otten, R. Fröhlich, G. Kehr, D. W. Stephan, G. Erker, Frustrated Lewis pair behavior of intermolecular amine/$B(C_6F_5)_3$ pairs. *Organometallics* **31**, 2367–2378 (2012).

31. A. E. Ashley, A. L. Thompson, D. O'Hare, Non-metal-mediated homogeneous hydrogenation of CO_2 to CH_3OH. *Angew. Chem. Int. Ed.* **48**, 9839–9843 (2009).

32. S. D. Tran, T. A. Tronic, W. Kaminsky, D. M. Heinekey, J. M. Mayer, Metal-free carbon dioxide reduction and acidic C–H activations using a frustrated Lewis pair. *Inorg. Chim. Acta* **369**, 126–132 (2011).

33. M. Finze, E. Bernhardt, A. Terheiden, M. Berkei, H. Willner, D. Christen, H. Oberhammer, F. Aubke, Tris(trifluoromethyl)borane carbonyl, $(CF_3)_3BCO$–synthesis, physical, chemical and spectroscopic properties, gas phase, and solid state structure. *J. Am. Chem. Soc.* **124**, 15385–15398 (2002).

34. V. Fasano, M. J. Ingleson, Recent advances in water-tolerance in frustrated Lewis pair chemistry. *Synthesis* **50**, 1783–1795 (2018).

35. Y. Hoshimoto, S. Ogoshi, Triarylborane-catalyzed reductive *N*-alkylation of amines: A perspective. *ACS Catal.* **9**, 5439–5444 (2019).

36. D. Voicu, D. W. Stephan, E. Kumacheva, Microfluidic separation of ethylene and ethane using frustrated Lewis pairs. *ChemSusChem* **8**, 4202–4208 (2015).

37. W.-B. Wang, S.-M. Lu, P.-Y. Yang, X.-W. Han, Y.-G. Zhou, Highly enantioselective iridium-catalyzed hydrogenation of heteroaromatic compounds, quinolines. *J. Am. Chem. Soc.* **125**, 10536–10537 (2003).

38. We also confirmed that the hydrogenation of **Qin** under otherwise identical mixed gas conditions did not proceed effectively and that **H_4-Qin** was obtained only in ~1% when Ru-MACHO and Pd/C were used as catalysts. For experimental details, see the Supplementary Materials.

39. M. Ullrich, A. J. Lough, D. W. Stephan, Reversible, metal-free, heterolytic activation of H_2 at room temperature. *J. Am. Chem. Soc.* **131**, 52–53 (2009).

40. A. E. Ashley, T. J. Herrington, G. G. Wildgoose, H. Zaher, A. L. Thompson, N. H. Rees, T. Krämer, D. O'Hare, Separating electrophilicity and Lewis acidity: The synthesis, characterization, and electrochemistry of the electron deficient *tris*(aryl)boranes $B(C_6F_5)_{3-n}(C_6Cl_5)_n$($n$= 1–3). *J. Am. Chem. Soc.* **133**, 14727–14740 (2011).

41. Á. Gyömöre, M. Bakos, T. Földes, I. Pápai, A. Domján, T. Soós, Moisture-tolerant frustrated Lewis pair catalyst for hydrogenation of aldehydes and ketones. *ACS Catal.* **5**, 5366–5372 (2015).

42. É. Dorkó, B. Kótai, T. Földes, Á. Gyömöre, I. Pápai, T. Soós, Correlating electronic and catalytic properties of frustrated Lewis pairs for imine hydrogenation. *J. Organomet. Chem.* **847**, 258–262 (2017).

43. P. A. Chase, L. D. Henderson, W. E. Piers, M. Parvez, W. Clegg, M. R. J. Elsegood, Bifunctional perfluoroaryl boranes: Synthesis and coordination chemistry with neutral lewis base donors. *Organometallics* **25**, 349–357 (2006).

44. M. Kojima, M. Kanai, Tris(pentafluorophenyl)borane-catalyzed acceptorless dehydrogenation of N-heterocycles. *Angew. Chem. Int. Ed.* **55**, 12224–12227 (2016).

45. A. F. G. Maier, S. Tussing, T. Schneider, U. Flörke, Z.-W. Qu, S. Grimme, J. Paradies, Frustrated Lewis pair catalyzed dehydrogenative oxidation of indolines and other heterocycles. *Angew. Chem. Int. Ed.* **55**, 12219–12223 (2016).

46. Y. Liu, H. Du, Metal-free borane-catalyzed highly stereoselective hydrogenation of pyridines. *J. Am. Chem. Soc.* **135**, 12968–12971 (2013).

47. P. Eisenberger, B. P. Bestvater, E. C. Keske, C. M. Crudden, Hydrogenations at room temperature and atmospheric pressure with mesoionic carbene-stabilized borenium catalysts. *Angew. Chem. Int. Ed.* **54**, 2467–2471 (2015).

48. The effect of using quinoline as the reaction medium was also simulated using the polarizable continuum model (PCM), which provided virtually identical results. For details, see the Supplementary Materials.

49. M. H. Reineke, M. D. Sampson, A. L. Rheingold, C. P. Kubiak, Synthesis and structural studies of nickel(0) tetracarbene complexes with the introduction of a new four-coordinate geometric index, τ_δ. *Inorg. Chem.* **54**, 3211–3217 (2015).

50. J. T. Manka, P. Kaszynski, Synthesis and thiolation of 1,3-difluoro-2,4,6-trihaloanilines and benzenes. *J. Fluor. Chem.* **124**, 39–43 (2003).

51. P. Sánchez, M. Hernández-Juárez, N. Rendón, J. López-Serrano, L. L. Santos, E. Álvarez, M. Paneque, A. Suárez, Hydrogenation/dehydrogenation of N-heterocycles catalyzed by ruthenium complexes based on multimodal proton-responsive CNN(H) pincer ligands. *Dalton Trans.* **49**, 9583–9587 (2020).

52. M. A. Beckett, G. C. Strickland, J. R. Holland, K. S. Varma, A convenient n.m.r. method for the measurement of Lewis acidity at boron centres: Correlation of reaction rates of Lewis acid initiated epoxide polymerizations with Lewis acidity. *Polymer* **37**, 4629–4631 (1996).

53. Gaussian 16, Revision C.01, M. J. Frisch, G. W. Trucks, H. B. Schlegel, G. E. Scuseria, M. A. Robb, J. R. Cheeseman, G. Scalmani, V. Barone, G. A. Petersson, H. Nakatsuji, X. Li, M. Caricato, A. V. Marenich, J. Bloino, B. G. Janesko, R. Gomperts, B. Mennucci, H. P. Hratchian, J. V. Ortiz, A. F. Izmaylov, J. L. Sonnenberg, D. Williams-Young, F. Ding, F. Lipparini, F. Egidi, J. Goings, B. Peng, A. Petrone, T. Henderson, D. Ranasinghe, V. G. Zakrzewski, J. Gao, N. Rega, G. Zheng, W. Liang, M. Hada, M. Ehara, K. Toyota, R. Fukuda, J. Hasegawa, M. Ishida, T. Nakajima, Y. Honda, O. Kitao, H. Nakai, T. Vreven, K. Throssell, J. A. Montgomery Jr., J. E. Peralta, F. Ogliaro, M. J. Bearpark, J. J. Heyd, E. N. Brothers, K. N. Kudin, V. N. Staroverov, T. A. Keith, R. Kobayashi, J. Normand, K. Raghavachari, A. P. Rendell, J. C. Burant, S. S. Iyengar, J. Tomasi, M. Cossi, J. M. Millam, M. Klene, C. Adamo, R. Cammi, J. W. Ochterski, R. L. Martin, K. Morokuma, O. Farkas, J. B. Foresman, D. J. Fox, Gaussian, Inc., Wallingford CT, 2019.

54. J. D. Chai, M. H. Gordon, Long-range corrected hybrid density functionals with damped atom–Atom dispersion corrections. *Phys. Chem. Chem. Phys.* **10**, 6615–6620 (2008).

55. J. Tomasi, B. Mennucci, R. Cammi, Quantum mechanical continuum solvation models. *Chem. Rev.* **105**, 2999–3094 (2005).

56. B. Cordero, V. Gómes, A. E. Platero-Prats, M. Revés, J. Echeverría, E. Cremades, F. Barragán, S. Alvarez, Covalent radii revisited. *Dalton Trans.* 2832–2838 (2008).
